# Health Benefits and Cost-Effectiveness From Promoting Smartphone Apps for Weight Loss: Multistate Life Table Modeling

**DOI:** 10.2196/11118

**Published:** 2019-01-15

**Authors:** Christine Cleghorn, Nick Wilson, Nisha Nair, Giorgi Kvizhinadze, Nhung Nghiem, Melissa McLeod, Tony Blakely

**Affiliations:** 1 BODE^3^ Programme Department of Public Health University of Otago Wellington New Zealand

**Keywords:** weight loss diet, telemedicine, smartphone, cost-utility analysis, life tables, quality-adjusted life years

## Abstract

**Background:**

Obesity is an important risk factor for many chronic diseases. Mobile health interventions such as smartphone apps can potentially provide a convenient low-cost addition to other obesity reduction strategies.

**Objective:**

This study aimed to estimate the impacts on quality-adjusted life-years (QALYs) gained and health system costs over the remainder of the life span of the New Zealand population (N=4.4 million) for a smartphone app promotion intervention in 1 calendar year (2011) using currently available apps for weight loss.

**Methods:**

The intervention was a national mass media promotion of selected smartphone apps for weight loss compared with no dedicated promotion. A multistate life table model including 14 body mass index–related diseases was used to estimate QALYs gained and health systems costs. A lifetime horizon, 3% discount rate, and health system perspective were used. The proportion of the target population receiving the intervention (1.36%) was calculated using the best evidence for the proportion who have access to smartphones, are likely to see the mass media campaign promoting the app, are likely to download a weight loss app, and are likely to continue using this app.

**Results:**

In the base-case model, the smartphone app promotion intervention generated 29 QALYs (95% uncertainty interval, UI: 14-52) and cost the health system US $1.6 million (95% UI: 1.1-2.0 million) with the standard download rate. Under plausible assumptions, QALYs increased to 59 (95% UI: 27-107) and costs decreased to US $1.2 million (95% UI: 0.5-1.8) when standard download rates were doubled. Costs per QALY gained were US $53,600 for the standard download rate and US $20,100 when download rates were doubled. On the basis of a threshold of US $30,000 per QALY, this intervention was cost-effective for Māori when the standard download rates were increased by 50% and also for the total population when download rates were doubled.

**Conclusions:**

In this modeling study, the mass media promotion of a smartphone app for weight loss produced relatively small health gains on a population level and was of borderline cost-effectiveness for the total population. Nevertheless, the scope for this type of intervention may expand with increasing smartphone use, more easy-to-use and effective apps becoming available, and with recommendations to use such apps being integrated into dietary counseling by health workers.

## Introduction

Obesity is an important risk factor for many chronic diseases that impact people’s quality of life and incur substantial health system costs. Obesity is an established risk factor for cardiovascular disease (CVD), diabetes, osteoarthritis, and various cancers [1].

Mobile health (mHealth) has been defined as “the application of mobile technologies, including phones, tablets, telemonitoring, and tracking devices, to support and enhance the performance of health care and public health practice” [2]. In the modeling study presented here, mHealth refers to using smartphone apps to deliver diet, exercise and health information, and behavior change support to participants to help them lose weight. mHealth tools can be accessed at people’s convenience, from their homes or using their phones on the go. These interventions, therefore, have the potential to provide more regular information than face-to-face weight loss programs and may therefore be an important low-cost addition to current obesity reduction strategies.

mHealth technologies have the potential to improve public health in the future despite the current absence of strong evidence of effectiveness [3]. The evidence base for the effectiveness of smartphone apps for weight loss is, however, growing. A systematic review including interventions that utilized smartphone apps, text messaging, and Web resources was published in 2014 [4]. It included 12 primary studies of randomized controlled trials (RCTs) investigating mHealth weight loss interventions through diet and physical activity. The meta-analysis of these 12 studies estimated an additional 0.43 kg weight loss (95% CI: 0.25-0.61) for the intervention groups compared with controls. However, evidence on how long and at what magnitude this weight loss persists is poor, an important source of uncertainty we explore in this paper as it will have an impact on future health gains and costs or cost savings.

Another key determinant of overall population impact is the uptake of smartphone apps. Uptake will depend on marketing, placement, and word-of-mouth. Governments or health system providers could also promote use of apps, particularly if such promotion increases health benefits cost-effectively. Therefore, the aim of this study was to estimate the likely future health impacts, costs, and cost-effectiveness of mass media promotion of mHealth programs that use smartphone apps to deliver health information and behavior change support to participants for weight loss, compared with the existing levels of promotion and use of mHealth in a fairly typical developed country setting: New Zealand. A secondary aim was to identify targets for future research that would improve the precision of cost-effectiveness modeling for these types of interventions.

## Methods

### Overview

Outputs from this modeling include incremental quality-adjusted life-years (QALYs) gained and costs or cost savings in New Zealand dollars (NZ $). Outputs were discounted at 3%, with 0% and 6% used in scenario analyses. A health system perspective was used, and benefits and costs were modeled using a lifetime horizon. The intervention was modeled as a one-off intervention implemented in 1 year in overweight and obese New Zealand adults.

To estimate the difference in QALYs and health system costs between the model’s intervention and business-as-usual (BAU) comparator, the entire New Zealand population, alive in 2011, was simulated out until death using a dietary multistate life table (MSLT) model built in Excel. The structure and BAU inputs for this generic model are described in detail in the model’s technical report (see Multimedia Appendix 1 [5]). The remainder of this Methods section provides a summary of general structure and BAU inputs and more details on specific intervention parameters for this mHealth intervention.

The BAU comparator was assumed to include the existing level of mHealth promotion—which is negligible in New Zealand (ie, we are aware of no health agencies in the country that promote specific smartphone apps for weight loss). Therefore, we did not strip the baseline component of the model back to a hypothetical “no mHealth” comparator. Briefly, the BAU model uses projected all-cause mortality and morbidity rates by sex and age and separately for Māori (indigenous population) and non-Māori ethnic groups. Running alongside this main life table were 14 body mass index (BMI)–related disease life tables, where proportions of the population simultaneously resided: coronary heart disease (CHD), stroke, type 2 diabetes, osteoarthritis, and multiple cancers (ie, endometrial, kidney, liver, esophageal, pancreatic, thyroid, colorectal, breast, ovarian, and gallbladder). The proportion of the New Zealand population in each disease life table was a function of the disease incidence, case fatality, and remission (the latter in cancers only).

The intervention was modeled as a one-off smartphone app promotion that occurred in year one, 2011. The intervention effect was captured through changes in BMI resulting from the mHealth intervention. The change in BMI was then combined with relative risks for the associations between BMI and diseases through population impact fractions (PIFs; percentage reductions in future BMI-related disease incidence) that alter the inflow to the BMI-related disease life tables. Time lags from change in BMI to change in disease incidence were allowed for by using the average BMI change over a previous window of time of 0 to 5 years for CVD, diabetes, and osteoarthritis and 10 to 30 years for cancers. Probabilistic uncertainty about the boundaries (5, 10, and 30 years) was also specified (see Multimedia Appendix 1 [5]).

### Input Parameters

#### Business-As-Usual Parameters

All input parameters (specified by sex, age, and ethnicity unless stated differently) are shown in Table 1 and described in more detail in Multimedia Appendix 1 [5]. Briefly, each BMI-related disease had incidence, prevalence, and case fatality in 2011. Remission rates were specified for cancers but set to 0 for chronic diseases of CHD, stroke, type 2 diabetes, and osteoarthritis (ie, lifetime diagnoses). These parameters were calculated using DISMOD II (World Health Organization 2001-2009, created by Jan J Barendregt) [6], which is a program used to calculate epidemiologically and mathematically coherent sets of parameters for each disease. Future trends in cancer incidence, case fatality, and remission were specified using regression estimates of trends from historic data. Trends in other diseases were obtained from the New Zealand Burden of Disease Study (NZBDS) [7].

Morbidity was quantified (separately by sex, age, and ethnic groups) for each disease using the years of life lived with disability (YLDs) from the NZBDS, divided by the population count to give prevalent YLDs. Disability weights from the Global Burden of Disease Study 2010 were used to estimate the health status valuation of these YLDs [8].

Health system costs (sex- and age-specific) were calculated in 2011 in NZ $ using individually linked data for publicly funded (and some privately funded) health events occurring in 2006 to 2010, including hospitalizations, inpatient procedures, outpatients, pharmaceuticals, laboratories, and expected primary care usage. Building on an existing framework [9] for calculating the timing of health system costs, the whole cohort was assigned an (sex- and age-specific) annual health system cost of a citizen without a BMI-related disease and not in the last 6 months of their life. Additional disease-specific excess costs were assigned to people (1) in the first year of a BMI-related disease diagnosis, (2) in the last 6 months of life if dying of the given disease, and (3) otherwise prevalent cases of each disease. Costs were modeled over the lifetime of the cohort, including costs both related and unrelated to the BMI-related diseases modeled (meaning increased longevity because of weight loss interventions contributes to increased health system costs for some cohort members). Organisation for Economic Cooperation and Development purchasing power parity for 2011 was used when costs were converted to US dollars (US $1.486 to NZ $1).

### Intervention Parameters

In this study, mHealth programs are those that use smartphone apps to deliver health information and behavior change support to participants for weight loss. It was assumed that smartphone apps would be promoted nationally through 1 main medium. First, weblinks to the best 5 iOS and 5 Android weight loss apps (all under NZ $4 to download), as recently identified through a New Zealand study [12], would be displayed on the Ministry of Health (MoH) and other health promotion websites. These apps largely work through their calorie counting and exercise tracking features with extra tips and support features. Promotion of these apps would be through a government-funded mass media campaign.

**Figure 1:**
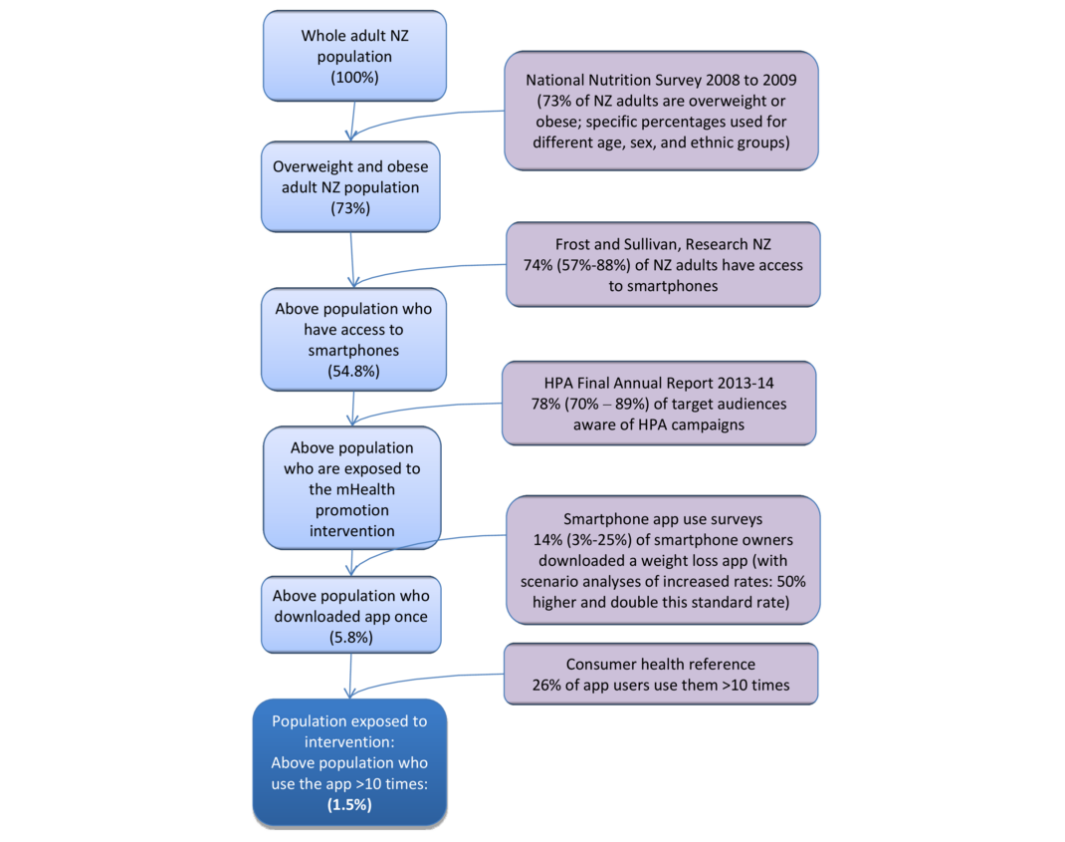
Flow diagram illustrating the targeting of the smartphone weight loss app promotion intervention in the New Zealand adult population. HPA: Health Promotion Agency; mHealth: mobile health; NZ: New Zealand.

The proportion of the population that would receive this intervention and how this is calculated is presented in Figure 1. The target population was overweight or obese adults (the target population was those older than 18 years, but relative risks for the association between BMI and disease apply from age 25 years onward) living in New Zealand, who have access to smartphones and who want to try and lose weight. The proportion of New Zealand adults (aged >18 years) who were overweight or obese was taken from the National Nutrition Survey (2008-2009) and was estimated by sex, ethnic, and age groups. The proportion of this population who take up this intervention was calculated as follows.

First, it was estimated that 74.42% (with an uncertainty interval [UI] of 57.49%-88.19%; see Table 2) of the population have access to smartphones apps. A total of 2 estimates of smartphone usage were used: a survey carried out by Research New Zealand [13] reporting 59% smartphone ownership or access by New Zealand adults in 2013 and a forecast of 90% smartphone access by New Zealanders by 2018 [14]. (We use 2011 baseline data but have used more current smartphone usage in New Zealand to give more relevant outputs).

Second, the number of people that would be reached through a mass media campaign was estimated based on the reach of a number of previous national-level Health Promotion Agency (HPA) campaigns listed below. The HPA is a state-funded organization that leads programs to promote health in New Zealand.

- For the HPA Heart and Diabetes Checks campaign in 2013, 70% of the core audience was reached at least once each month, with 50% of the target audience seeing the commercials 3 times [15].
- A total of 2 alcohol awareness campaigns achieved 89% total awareness of the key marketing messages, and 75% of adults were made aware of key elements of an alcohol law change [15].
- A television campaign on rheumatic fever prevention reached 76% of the target audience (parents and caregivers of at-risk children and young people) [16].

Our estimate of the likely reach of the mass media campaign promoting the smartphone apps for weight loss was based on an average of these mass media campaign figures with uncertainty spanning its range: a central estimate of 77.94% with a UI of 70.00% to 89.00%. Nearly half (46.8%) of all New Zealand adults (78% of the 60% of New Zealanders that have access to smartphones) were assumed to be exposed to the promotion of the intervention and have a smartphone. This is probably a relatively conservative estimate as there may be additional reach through mechanisms we did not model, that is, via Web-based activity as suggested in the HPA healthy eating program (the average total reach for the HPA healthy eating program for all Web-based activity for 2013-14 was 2,272,525 hits per month [15]), through referrals by health professionals, and through word-of-mouth.

**Table 1:** Baseline input parameters used in modeling the promotion of smartphone apps for weight loss.

Key parameter	Source and application to model	Uncertainty	Distribution and heterogeneity
Baseline population count	Statistics New Zealand (SNZ) population estimates for 2011	Nil uncertainty	Sex; age; ethnicity
All-cause mortality rates	SNZ mortality rates for 2011	Nil uncertainty	Sex; age; ethnicity
Disease-specific incidence, prevalence, case fatality rates, and remission rates	For each disease, coherent sets of incidence rates, prevalence, case fatality rates (CFR), and remission rates (zero for noncancers, the complement of the CFR for cancers to give the expected 5-year relative survival) were estimated using DISMOD II using data from New Zealand Burden of Disease Study (NZBDS), HealthTracker, and the Ministry of Health	Uncertainty: rates all ±5% SD	Log-normal; sex; age; ethnicity
Disease trends	Trends are applied to incidence, case fatality, and remission. These are switched on until 2026 and then kept constant for the remainder of the lifetimes of the modeled population	Uncertainty ±0.5% absolute change; diabetes: uncertainty ±1.5% absolute change	Normal; sex; ethnicity
Total morbidity per capita in 2011	The per capita rate of years of life lived with disability (YLD) from the NZBDS	Uncertainty ±10% SD	Log-normal; sex; age; ethnicity
Disease morbidity rate per capita	2006 NZBDS (projected to 2011); each disease was assigned a disability rate (DR; by sex and age) equal to YLDs for that disease (scaled down to adjust for comorbidities) from the 2006 NZBDS projected forward to 2011, divided by the disease prevalence. This DR was assigned to the proportion of the cohort in each disease state	Uncertainty: ±10% SD	Normal; sex; age
Health system costs	Linked health data (hospitalizations, inpatient procedures, outpatients, pharmaceuticals, laboratories, and expected primary care usage) for each individual in New Zealand for the period 2006 to 2010 had unit costs assigned to each event, and then health system costs (NZ $2011) were estimated	Estimated at SD ±10% of the point estimate	Gamma; sex; age
Time lags for intervention effect	It takes time for a change in body mass index (BMI) to impact on disease incidence. As there are no precise data on just how long these are, we have used wide windows of time lags. For cancers, the time lag is assumed to range between 10 and 30 years. For CHD, stroke, diabetes, and osteoarthritis (the noncancers), the time lag is assumed to be shorter and ranges between 0 and 5 years. Wide uncertainty is included around these estimates	Uncertainty: ±20% SD	Normal
BMI theoretical minimum risk exposure level (TMREL)	TMREL is the level of risk exposure that is theoretically possible and minimizes overall risk and is derived from the latest Global Burden of Disease 2013 study [10]. This allows us to estimate how much of the disease burden could be lowered by shifting the distribution of a risk factor to the level that would lead to the greatest improvement in population health	Uncertainty: uniform distribution between 0 and 1	Uniform
Height of the New Zealand adult population (for BMI calculations)	Mean and SD of height from the New Zealand Adult Nutrition Survey 2008 to 2009 [11]	Uncertainty using reported SD	Normal; sex; ethnicity

We used 2 US surveys which reported the percentage of smartphone owners who have downloaded a weight loss app to estimate the proportion of the above population that would download a weight loss app in this study. Fronstin [17] reports 25% of smartphone owners with private health insurance had used a weight management or diet app. In an internet report published by Pew Research Center, 12% of the 19% of smartphone owners that had reported downloading a health app downloaded a weight loss app [18]. This equates to 2.3% of all smartphone owners. This is a wide range of estimates and reflects baseline use of weight loss apps in the US population (not the impact of mass media campaigns) but is the best evidence we could identify as currently available. Wide uncertainty has been incorporated into the estimate to reflect this. The average (13.46% with a UI of 2.50%-25.00%; see Table 2) of these 2 figures was used as an estimate of the overweight and obese smartphone owners who have been reached by the mass media campaign, who are likely to download a weight loss app. Under this standard rate of app downloads, the mass media promotion does not increase the proportion of the target population downloading weight loss apps, but may still change the type of app downloaded to those promoted.

As this figure represents the usual download rate without a mass media campaign, we modeled a range of different but still plausible download rates: standard download rate (the base-case), 50% increase in downloading, and doubling of this download rate, with corresponding proportional UIs.

A Web survey in 2011 by the Consumer Health Information Corporation (N=395) in the United States found that 26% of downloaded smartphone apps are used repeatedly (ie, 10 times or more) [19]. Assuming this applies to overweight or obese people in New Zealand, this gave 1.36% of the target population who are likely to use the app more than 10 times (see Figure 1; note that in this figure, the final percentage of the population that is exposed to the intervention is 1.5% based on the average proportion of New Zealand adults that are overweight or obese, and when this number is calculated in the model using population weightings, it is 1.36%). Uncertainty around previously outlined parameters contributed to uncertainty around this final figure (1.36% [0.39%-2.99%]). See Figure 1 for a pictorial summary of this population selection process. All parameters used to target this intervention were the same for all age, sex, and ethnic groups except for the proportion of the population that is overweight or obese, which differs by age, sex, and ethnicity.

**Table 2:** Intervention input parameters used in modeling the promotion of smartphone apps for weight loss.

Parameters	Source and application to model	Expected value and uncertainty	Distribution and heterogeneity
Effect size	The meta-analysis generated an effect size of 0.43 kg (95% CI 0.25-0.61) of mobile device interventions compared with control groups [4]; effect size operational only for overweight and obese adults in the model. Effect size in kg was converted to body mass index (BMI) using average heights for the New Zealand population for the 4 demographic groups	0.43 kg (95% CI 0.25-0.61)	Normal
BMI decay	Meta-analysis evidence of weight loss decay [20]; at the end of intervention delivery, the modeled BMI reduction decays back to the preintervention BMI at a rate of 0.03 units per month	Uncertainty±20% SD	Log-normal
Proportion of New Zealanders with smartphones	Frost and Sullivan press release [14], Research New Zealand [13]	74.42% (57.49%-88.19%), CI based on the range of estimates available	Beta
Proportion of the above population who are likely to be exposed to the mobile health (mHealth) promotion intervention	Heath Promotion Agency final annual report 2013-14 [15]	77.94% (70.00%-89.00%), CI based on the range of estimates available	Beta
Above population who are likely to have downloaded a weight loss app once	Smartphone app use surveys [18]	13.46% (2.50%-25.00%), CI based on the range of estimates available	Beta
Above population who use the app >10 times	Consumer health information corporation [19]	26%; uncertainty±SD (SD: 20% of mean)	Beta
Intervention costs	Total intervention costs are NZ $2,883,000	Uncertainty±SD (SD: 20% of mean)	Gamma
Relative risks for BMI and disease incidence	See Multimedia Appendix 1 [5] for disease-specific relative risks		Sex, age

The effect size for reduction in BMI among *successful* app users (ie, 10 or more uses) was taken from a systematic review of RCTs for mobile devices and weight loss in adults [4]. It included 17 RCTs, 12 of which were primary studies and 5 were secondary analyses of primary studies. Of the 12 studies, 8 used a mobile phone as the intervention medium, specifically smartphones. Of these 12 studies, 9 targeted both diet and physical activity to induce weight loss. The remaining 3 studies concentrated primarily on physical activity to induce weight loss, whereas none of the studies targeted just dietary change. Intervention duration for the 12 studies (ie, not including follow-up) ranged from 4 weeks to 2 years, and studies were all carried out in high-income countries: the United Kingdom, United States, Finland, and Australia. All included studies used an intention-to-treat analysis in accordance with the original assignment. Interventions included a variety of approaches including weight, energy intake and energy expenditure goal setting and self-monitoring, text reminders on various topics, meal and physical activity planning, buddy components, trophy rooms for rewards, and even some group sessions and calls from counselors. The meta-analysis of these 12 studies with weight as the outcome estimated an additional 0.43 kg weight loss (95% CI 0.25-0.61) for the intervention groups compared with controls. This weight change was converted to a change in BMI using average height for the 4 demographic groups in the MSLT model (Māori men, Māori women, non-Māori men, non-Māori women).

The effect size of −0.43 kg (−0.61 to −0.25) equated to a reduction in 0.14 to 0.17 BMI units across sex by ethnic groups in those successfully completing the mHealth intervention. Regarding decay of effect, the trials included in the systematic review [4] did not measure maintenance of the weight loss over time. A meta-analysis of face-to-face dietary advice by Dansinger et al [20] found that BMI increased by 0.03 BMI units per month post dietary counseling from an initial BMI decrease of 1.9 units. Evidence on how weight regain differs by type of weight loss intervention and magnitude of initial weight loss is currently limited, so we used this 0.03 BMI units per month as an estimate of how the effect of the mHealth intervention would decay post intervention. With such a small initial effect size, the BMI decrease returned to 0 approximately 5 months post the year of the intervention.

#### Intervention Costs

For this intervention, it was assumed that already existing smartphone apps (as identified in a recent survey [12]) were promoted, which avoids costs associated with any new software development. Costs of a media campaign by the MoH or the HPA have been estimated from previous health promotion media campaigns (Table 3).

#### Relative Risks

The change in BMI was then combined with the disease-specific relative risks (see Multimedia Appendix 1 [5]) through PIFs, which altered the incidence of BMI-related diseases.

**Table:** Costs associated with the smartphone app for weight loss promotion intervention.

Cost component^a^	Cost (NZ $)	Details
One-off costs for the promotion of the smartphone apps	$72,000	The cost of promotion on relevant government-funded websites (Ministry of Health, district health board, Health Promotion Agency [HPA]). Estimate based on the HPA Breakfast-eaters campaign (Personal Communication, HPA, October 2015) for Web-based promotions (Google adwords, Facebook adverts, promoting Facebook posts, etc) to drive consumers to the Breakfast-eaters website
Mass media promotion	$2,791,000	Cost of 1 year mass media promotion (assumed to be the same as the 2013-14 Quitline marketing budget; the promotion required for this intervention was assumed to be similar to the level of marketing undertaken by Quitline); $2,887,000) [21]. The cost of Quitline advertising and promotion for the 12-month period in 2013-2014 was NZ $2,165,000, and the staff management costs for “marketing and communications” were NZ $722,000 [21]. These 2014 costs were consumer price index–adjusted to the 2011 base year, giving an annual cost of NZ $2,791,000
Identifying top apps	$20,000	Cost of a one-off upgrade of previous New Zealand work [12] in identifying the top 5 apps for Apple and Android weight loss apps for promotion on the websites (NZ $20,000 contract)
Total intervention costs	$2,883,000	Uncertainty: estimated at SD±20% of the point estimate, gamma distribution. Correlated (0.75) with intervention parameters (access to smartphones, exposure to promotion campaign, and weight loss app downloaded)

^a^Costs to the individual were not included as they were out of scope with the health system perspective used but would include a proportion of the cost of a smartphone and its running costs, the usually trivial cost of the app (though most are free) and any costs (or cost-savings) for dietary changes and increased physical activity.

### Modeling and Analysis

Microsoft Excel using an Ersatz add-in (Epigear International, created by Jan J Barendregt) was used to run each of the scenarios presented with uncertainty through the model 2000 times. Each of these simulations involved a random draw from the probability density function about those parameters specified with uncertainty in Tables 1 and 2. The main results produced were incremental QALYs gained and net health system costs accrued. The net health system cost was the sum of the intervention cost and any difference in projected future health system expenditure resulting from changes in disease incidence because of the mHealth intervention (including extra health costs from any increased life span).

## Results

The estimated impact of the base-case intervention was a health gain of 29 QALYs (95% UI: 14-52; with 3% discounting) and costs to the health system of NZ $2.3 million (95% UI: NZ $1.6-3.0 million, US $1.6 million [95% UI: 1.1-2.0]) over the lifetime of the modeled population (Table 4). This was assuming the standard rate of app downloading (ie, the mass media promotion does not increase the proportion of the target population downloading weight loss apps but may still change the type of app downloaded to those promoted). QALY gains increased to 45 (95% UI: 21-81) and 59 (95% UI: 27-107) when the proportion of the target population downloading the app was modified in plausible directions, that is, increased by 50% from the standard download rate and doubled, respectively. Costs decreased to NZ $2.0 million (95% UI: NZ $1.1-2.8 million, US $1.4 million [95% UI: 0.7-1.9]) and NZ $1.8 million (95% UI: NZ $0.7-2.6 million, US $1.2 million [95% UI: 0.5-1.8]), respectively.

Costs per QALY gained (or the incremental cost-effectiveness ratio) were NZ $79,700 (US $53,600) for the standard download rate, NZ $45,500 (US $30,600) for the 50% increase in download rate, and NZ $29,900 (US $20,100) for the doubling download rate scenarios. On the basis of a threshold of NZ $45,000 (US $30,000), this intervention would appear to be of borderline cost-effectiveness for the total population and cost-effective for Māori when standard download rates increased by 50%. The intervention was cost-effective when download rates doubled as a result of the mass media campaign.

QALYs and associated costs were similar between men and women. As Māori make up only 15% of the total population in New Zealand, the majority of absolute QALYs gained and costs occurred in the non-Māori population. Health gains for the target population, those that are overweight or obese, were 0.011 QALYs per 1000 people for standard download rates and 0.021 QALYs per 1000 people when download rates were doubled. The age-standardized per capita QALY gains from the intervention for Māori were double of those for non-Māori at 0.010 per 1000 population for Māori and 0.005 for non-Māori in the base-case and were 0.021 in Māori and 0.009 in non-Māori when download rates were doubled. Adjusting for higher background mortality and morbidity rates for Māori, in an equity analysis where non-Māori mortality and morbidity rates were applied to Māori [22], QALYs gained for Māori increased from 5 to 6 total QALYs.

Undiscounted base-case results gave a health gain of 55 QALYs as a result of the intervention (Table 5) and 19 QALYs with 6% discounting. In the hypothetical scenario where weight loss is maintained over time, the total QALYs gained would increase to 2420 over the lifetime of the cohort and provide NZ $44.3 million in cost savings.

**Table 4:** Health gain (in quality-adjusted life-years) and health system costs saved over the life course from the promotion of smartphone apps for weight loss among the New Zealand population alive in 2011 (population N=4.4 million; 3% discounting; 95% UI in brackets). Results presented for those older than 25 years as relative risks for the associations between risk factors and disease start at age 25 years.

Subpopulation		Non-Māori	Māori	Ethnic groups combined	
		QALYs^a^	QALYs	QALYs	Net costs to the health system (NZ $ million)^b^
**Base-case with no increase in standard downloading of apps (13.5% of exposed population download a weight loss app)**					
	All	24 (10-47)	5 (2-10)	29 (14-52)	2.3 (1.6-3.0)
	Men	12	2	14	1.1
	Women	12	3	15	1.2
	Per capita^c^	0.006 (0.005)	0.007 (0.009)	0.007	0.53
	Per capita for those overweight and obese^c^	0.010 (0.005)	0.011 (0.010)	0.011	0.84
**Scenario: 50% increase in standard downloading of apps (20.3% of exposed population download a weight loss app)**					
	All	37 (15-73)	8 (3-15)	45 (21-81)	2.0 (1.1-2.8)
	Men	18	4	22	1.0
	Women	19	4	23	1.0
	Per capita^c^	0.010 (0.008)	0.011 (0.014)	0.010	0.46
	Per capita for those overweight and obese^c^	0.016 (0.007)	0.016 (0.016)	0.016	0.73
**Scenario: doubling the standard downloading of apps (27.0% of exposed population download a weight loss app)**					
	All	49 (19-97)	10 (4-20)	59 (27-107)	1.8 (0.7-2.6)
	Men	24	5	29	0.9
	Women	25	5	30	0.9
	Per capita^c^	0.013 (0.010)	0.015 (0.018)	0.013	0.40
	Per capita for those overweight and obese^c^	0.021 (0.009)	0.021 (0.021)	0.021	0.63

^a^QALYs: quality-adjusted life-years.

^b^Includes both the cost offsets and intervention cost (see Table 3), distributed pro rata across all people alive in 2011.

^c^All per capita results are QALYs per 1000 adults and NZ $ per adult. Results in brackets for Māori and non-Māori are age-standardized. Results rounded to either 2 or 3 meaningful digits.

**Table 5:** Scenario analyses about health gain in quality-adjusted life-years and health system costs for the promotion of smartphone apps for weight loss compared with business as usual (expected value analysis; no uncertainty).

Scenario		QALYs^a^ gained	Net costs to the health system (NZ $ million)
Base-case model^b^		30	2.3
**Discount rate**			
	0% per annum	55	2.1
	6% per annum	19	2.4
No decay in intervention benefit (permanent weight loss)		2420	−44.3 (ie, cost saving)

^a^QALY: quality-adjusted life-years.

^b^Discount rate 3%, standard app download rates, and intervention effect decays at a rate of 0.03 body mass index (BMI) units per month.

**Figure 2:**
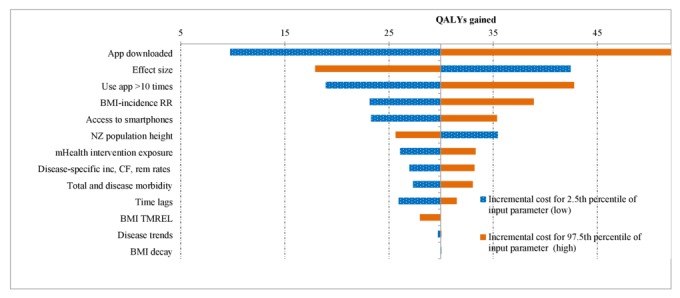
Tornado plot indicating which parameters drive uncertainty in the model results for health gain (in quality-adjusted life-years; QALYs) for the population. BMI: body mass index; CF: case fatality; inc: incidence; mHealth: mobile health; NZ: New Zealand; rem: remission; RR: relative risks; TMREL: theoretical minimum risk exposure level.

**Figure 3:**
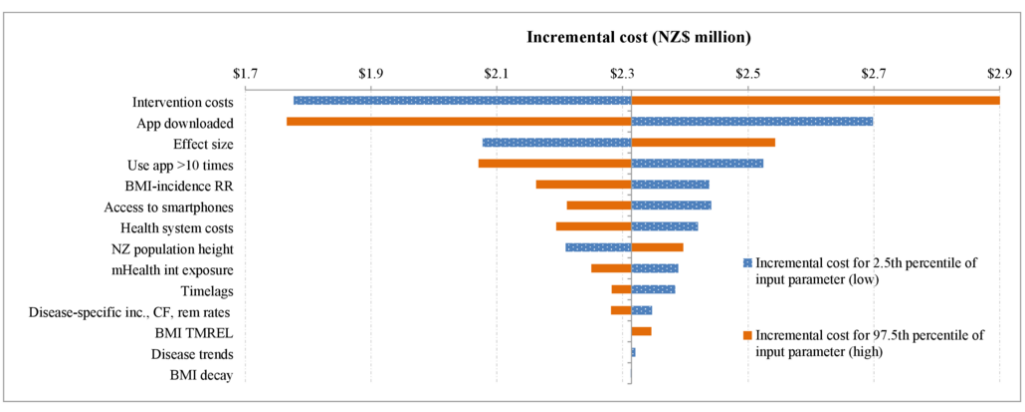
Tornado plot indicating which parameters drive uncertainty in the model results for health system costs for the population. BMI: body mass index; CF: case fatality; inc: incidence; mHealth: mobile health; NZ: New Zealand; rem: remission; RR: relative risks; TMREL: theoretical minimum risk exposure level.

Parameters contributing to the uncertainty in the model are shown in tornado plots in Figures 2 and 3. The parameters contributing the most to the uncertainty around the QALYs are whether the app was downloaded (which was varied in the main results presented in Table 4), the effect size, and the estimate of regular use of the app (defined as 10 or more uses). Other parameters that also contributed to overall uncertainty included uncertainty around the relative risks for the association between BMI and disease incidence; access to smartphones; the height of New Zealanders; estimates of the proportion of the target population being exposed to the mass media campaign; the disease-specific estimates of incidence, case fatality, and remission; and morbidity estimates. Uncertainty around the time lag between the intervention and onset of disease, the theoretical minimum risk exposure level around the association between BMI and disease, disease trends, and the BMI decay contributed the least to overall uncertainty.

Similar results are seen for the uncertainty contributing to the UIs around costs, but the biggest contributor was the intervention costs. The next top 3 parameters are as follows: (1) whether the app was downloaded, (2) the effect size, (3) and regular use of the app. Uncertainty around the disease-specific health system costs also makes a moderate contribution to the overall uncertainty.

## Discussion

### Principal Findings and Interpretation

The modeled intervention is based on a meta-analysis of mobile device interventions, which reported that those following the mHealth weight loss interventions lost an average of 0.43 kg (95% CI −0.61 to −0.25) more weight than the controls [4]. When modeled through to changes in incidences of BMI-associated diseases and then health gain in QALYs and health system costs, this intervention was found to be of borderline cost-effectiveness for the total population when download rates increased by 50% but cost-effective for Māori, based on threshold of NZ $45,000 per QALY gained. However, it would be cost-effective for the total population if download rates doubled (NZ $29,900 or US $20,100 per QALY) as a result of the mass media campaign. Further research to improve the estimate of download rates resulting from this type of campaign would be useful. The total impact on health was small, with total QALYs gained ranging from 29 to 59 over the remaining life span of 4.4 million people entering the simulation. Costs to the health system ranged from NZ $1.8 to NZ $2.3 million.

Even with higher download rates (and therefore higher health gain), the total health gain in these scenarios was still small from a population per capita perspective. For example, health gains seen with annual tobacco tax increases gave a total of 60,400 QALYs in the New Zealand population [23]. Reducing dietary salt intake by 35% (through mandatory maximum salt levels in packaged foods and reductions in salt through fast foods and restaurant food and discretionary intake) generated 235,000 QALYs [24] over the lifetime of the New Zealand population.

Assuming that the intervention itself is equally effective for Māori and non-Māori, age-standardized population per capita QALYs for Māori were double of those for non-Māori, reflecting higher rates of overweight and obesity in Māori compared with non-Māori. Even greater benefit for Māori could potentially be achieved from an app and promotional campaign designed specifically for this population group.

### Study Strengths and Limitations

The effect size used for modeling is an important parameter that drives the health and cost outputs presented. This was taken from a meta-analysis, the best quality evidence available, but the effect seen in the New Zealand context may vary from the meta-analysis effect size. The mHealth apps in the meta-analysis are different from the apps that would be promoted through this intervention if it was implemented in New Zealand. The meta-analysis effect size also included other mobile devices other than smartphones and other elements of mHealth. These differences between the modeled effect and the likely real effect are important to consider along with the fact that the uncertainty around the effect size was the second largest contributor to the overall uncertainty of the results. The lack of evidence on the effectiveness of a mass media campaign on download rates is another limitation of this work. This parameter contributes the most uncertainty to the health outcomes, and variation in this parameter changes the intervention from being cost-ineffective to being cost-effective. Therefore, future research regarding this parameter would better inform understanding of the cost-effectiveness of promoting smartphone apps for weight loss.

The modeled health benefits may actually be underestimated for a number of reasons. This study only models the effect of the intervention in those that actually take up the intervention, but there may be additional spill-over benefits to other members of the household through health-promoting changes in household meals or additional family physical activity. Furthermore, the impact of the intervention on physical activity itself was not modeled through to disease incidence. The intervention was also modeled as a one-off intervention when in reality the intervention could be ongoing, having the potential to both recruit more people over a number of years and/or sustain behavior change among initial participants. It is also likely that smartphone app usage and the quality of the apps available will increase over time, with effectiveness potentially increasing the effect size relative to that from the meta-analysis published in 2014. For example, higher-quality apps can integrate the collection of data on dietary energy intake with automatic estimates of energy expenditure based on the pedometer built into the smartphone. Additionally, the scope for this type of intervention may expand if smartphone weight loss apps were integrated with dietary counseling in primary care. These types of apps might become more integrated into daily routines and so weight loss achievements might be sustained for longer periods into the future.

The evidence for the rate at which the weight loss attenuates back to baseline, the BMI decay, is sourced from a meta-analysis [20] and is based on weight loss dietary counseling interventions. However, weight loss decay may differ for mHealth interventions where individuals can continue to access the app in the future, unlike with face-to-face dietary counseling, which is time limited. The scenario analysis where weight loss is maintained over the life course shows much greater health gains (2420 QALYs) and produces substantial cost savings (NZ $44.3 million). It is likely that the truth lies somewhere between the base-case and this scenario. Furthermore, as discussed above, app design and also changes to the obesogenic environment may impact the intervention decay rate. These factors may warrant additional research to improve estimation of health gains.

Finally, this study takes a health system perspective, but this intervention might result in wider societal benefits, for example, modifications made to people’s diets for weight loss could result in lower consumption of energy-dense dairy products and meat products, therefore reducing greenhouse gas emissions [25] and other livestock-related environmental damage (water use, water pollution, erosion, and reduced biodiversity). Improved health from a lower BMI and increased physical activity could also result in higher productivity in the workplace (eg, from reduced illness-related absenteeism, early retirement because of illness, and premature death before retirement age).

### Potential Implications for Research

As the field of mHealth develops, further research into the proportion of overweight and obese people who would regularly use an mHealth weight loss intervention, how this would be influenced by mass media campaigns, their subsequent weight loss, and how long their weight loss is maintained would all be useful. Consideration could also be given to determining app usage in the context of smartphone-based digital assistants, which can access apps (eg, Google’s “Google Assistant” and Apple’s “Siri”), or the provision of weight loss support from home-based digital assistants (eg, Amazon’s “Alexa”).

### Potential Implications for Health Agencies

The results of modeling this mHealth intervention suggest it is likely to have relatively small absolute health gains at a population level (given the current levels of app use and current app design). As such, smartphone weight loss apps should not be a priority for inclusion in current obesity reduction strategies. Resources should instead be prioritized toward cost-effective or cost-saving interventions likely to have greater health impacts, such as food or beverage taxes and subsidies [26], restrictions on marketing of unhealthy foods [27], and improved nutrition labeling [28]. The scope for smartphone weight loss apps may expand with increasing smartphone use, more easy-to-use and effective apps becoming available, and integration of app promotion with dietary counseling by health workers. mHealth for weight loss may therefore become a more viable component of obesity prevention strategies in the future.

## Appendix

Multimedia Appendix 1 Technical Report for BODE^3^ Diet Intervention and Multistate Lifetable Models Version 1.

## Abbreviations

BAU: business-as-usual
BMI: body mass index
CHD: coronary heart disease
CPI: consumer price index
CVD: cardiovascular disease
DHB: district health board
HPA: Health Promotion Agency
mHealth: mobile health
MoH: Ministry of Health
MSLT: multistate life table
NZBDS: New Zealand Burden of Disease Study
PIF: population impact fraction
QALY: quality-adjusted life-year
RCT: randomized controlled trial
UI: uncertainty interval
YLD: years of life lived with disability
