# Supplementary material for: Health Benefits and Cost-Effectiveness From Promoting Smartphone Apps for Weight Loss: Multistate Life Table Modeling
Source: JMIR Mhealth Uhealth. 2019 Jan 15;7(1):e11118. doi: 10.2196/11118 (PMC6350086; doi:10.2196/11118)

Public Health Monograph Series
No. 36
ISSN 1178-7139

Technical Report for BODE^3 Diet Intervention and Multistate Lifetable Models

Version 1

Burden of Disease Epidemiology, Equity and Cost-Effectiveness Programme (BODE^3)

Technical Report: Number 16

Cristina Cleghorn
Tony Blakely
Nhung Nghiem
Anja Mizdrak
Nick Wilson

Version 1: November 2017

A technical report published by the Department of Public Health,
University of Otago, Wellington

ISBN 978-0-9951070-0-7

Contact: Cristina Cleghorn
(Dietary modelling lead in BODE^3 Programme, University of Otago, Wellington, New Zealand).
Email: Cristina.cleghorn@otago.ac.nz

Table of Contents

Overview and Purpose 4

Part 1. Dietary Modelling 7

Section 1.01. Baseline diet data 7

1.01.1. Food consumption data - baseline 7

1.01.2. Food nutrient data 7

1.01.3. Price 8

Section 1.02. Dietary interventions parameterised as directly changing a risk factor 9

Section 1.03. Dietary interventions that change food consumption or composition 10

1.03.1. Food taxes and subsidies 10

1.03.2. Food reformulation 23

Part 2. Risk factors 24

Section 2.01. Risk factor distributions 24

2.01.1. Change in BMI 26

Part 3. Disease Modelling 27

Section 3.01. Model structure 27

3.01.1. Life-table analysis 27

3.01.2. Diseases included in BODE^3 DIET MSLT model 29

3.01.3. Diet-related disease models 30

3.01.4. How changes in risk factors change disease incidence 31

3.01.5. Diabetes: both a disease and a risk factor 38

Section 3.02. Baseline specification and parameters 43

3.02.1. Background population inputs 43

3.02.2. Data sources, processing, DISMOD, and inputs to BODE^3 DIET MSLT model 43

3.02.3. Final processing of incidence and prevalence estimates 46

3.02.4. Future disease trends (incidence, remission and case-fatality) 46

3.02.5. Disease health system cost inputs 47

Section 3.03. Calibration 47

Section 3.04. Validation 55

3.04.1. Face Validity 55
3.04.2. Verification (or Internal Validity) 55
3.04.3. Cross Validity 56
3.04.4. External Validity 57
3.04.5. Predictive Validity 57
Section 3.05. Model: Analysis 57
Section 3.06. A note on interchangeable use of 'DALYs averted' and 'QALYS gained' 58
Part 4. References 59
Appendix A: Food groups from the NZANS used in the BODE[3] intervention model 63
Appendix B: SPEND Study price elasticity table; aggregated food groups with Standard Errors 72
Appendix C: DISMOD II example for lung cancer 86
Appendix D: Parameterisation of 'DM as both a risk factor and disease' 89
Section 4.01. Baseline parametrization 89
4.01.1. Diabetes inputs to DISMOD: incidence, prevalence, mortality 89
4.01.2. Case fatalities operationalized in DM disease process 93
4.01.3. Preventing double-counting of BMI effects on DM and on CHD and stroke 93
Appendix E: Relative risks of diet to disease associations 95
Appendix F: Health system costs 100
Appendix G: Model rates vs. DISMOD rates, log graphs from Section 3.04.5 119

Overview and purpose

This Technical Report provides the documentation on the Burden of Disease Epidemiology Equity and Cost Effectiveness (BODE^3) DIET models.[i] The first intervention model (IM) estimates the effect of a range of *preventive dietary interventions* on risk factors. The second, the BODE^3 DIET multistate lifetable (MSLT) model, estimates the effect the change in risk factor has on *health impacts* and *cost impacts* of a range of interventions in the New Zealand population, with the ability to examine *heterogeneity by sex, age and ethnicity*.

- By *health impacts*, we mean a range of metrics. The primary metric is quality adjusted life years (QALYs) gained (or perhaps lost) by the intervention compared to modelled business as usual (BAU), but the following can also be outputted: mortality rates, morbidity rates, life years gained, and disease incidence.
- By *cost impacts*, we mean two levels. First, and as the main or default option, *health system perspective* costs. This is the net of both the intervention cost (e.g. the cost of a new law for new taxes or the cost of dietary counselling by practice nurses) and the downstream costs averted (or incurred) in the health system due to changing disease incidence and prevalence. Second, *societal* cost impacts, most notably productivity costs.[ii] This adds to the health system costs those costs due to gains (or losses) in productivity in the labour force through keeping people healthy to work. We plan to extend this to welfare benefit costs. Greenhouse gas emissions and other 'costs' will also be considered. We do not, however, extend out to monetary value of life as this is partially captured in the QALY metric.
- By *preventive dietary interventions* we mean public health or similar interventions that have the potential to change future dietary-related disease incidence. We consider these as two types of intervention: dietary interventions directly changing a 'risk factor', such as dietary counselling parametrized as directly changing body mass index (BMI; Section 1.02); dietary interventions that change food consumption or composition (and then change risk factors; Section 1.03).
- By *heterogeneity by sex, age and ethnicity* we mean that model outputs will be examined and contrasted by these demographic groups. Why? Several reasons: we are interested in the ability of population-wide interventions to reduce ethnic inequalities in health (and socioeconomic inequalities in the future); intervention effectiveness varies by background epidemiological parameters (e.g. if the cardiovascular disease [CVD] rate for a group is high, they stand to gain more); gains in QALYs (intervention effect held constant) will differ by background mortality and morbidity rates.

The conceptual structure of the combination of both of the BODE^3 models is shown below in Figure 1. Specific dietary interventions lead to change in foods consumed, and then to change in nutrients and physiological markers that in turn lead to changes in disease incidence. The dietary interventions are 'channelled through' selected foods (fruit and vegetables, sweetened sugary beverages (SSBs)),

---

[i] As of this version of the Technical Report, and models, the physical activity component is not yet developed – pending.
[ii] As of this version of the Technical Report, productivity costings are pending.

nutrients (sodium and PUFA) and BMI which have epidemiological associations with diseases as reported in the Global Burden of Disease (GBD).[1] [iii]

The modelling occurring in the BODE^3 DIET MSLT model itself is carried out for multiple cohorts in parallel, currently for the four ethnic (Māori, non-Māori) by sex sub-populations alive in 2011, for each five-year age group. This model structure can and will be adapted in the future to be for different sub-populations (e.g. just pre-diabetics within each of the four demographic groups, by socio-economic position), and will require substantial re-parametrization. In this Technical Report, we focus on the base-case sex-by-ethnic-by-age modelling.

The BODE^3 DIET model is a multi-state life-table (MSLT) 'macro-simulation' model. That means, we model averages or expected values for each cohort (i.e. sex by age by ethnic group). For example, the average age-specific coronary heart disease (CHD) rate in each of these groups is used. The model can capture some distributional aspect for risk factors, though. For example, the percentage of each starting sex by age by ethnic group in each BMI category is used when 'merging' all of intervention effect (e.g. a 0.1 BMI unit reduction across the population) with the relative risks (e.g. for CHD between BMI categories), to generate the population impact fraction (PIF; i.e. the percentage by which disease incidence will change given intervention effect, risk factor distribution and Relative Risk (RR) strength of association). This 'merging' also means we can target interventions by level of (say) BMI, by allowing the intervention effect to only be applied to obese people. However, the model does not explicitly allow for correlated joint distributions of risk factors in the general population, e.g. BMI and sodium intake are assumed to be independently distributed. A microsimulation model would be required for this level of sophistication and may be needed in the future – however, given the (often large) uncertainty in the association of dietary risk factors with disease, such sophistication may not be yet warranted.

This Technical Report is structured to mirror the conceptual model in Figure 1 below. Specifically:

- PART 1 describes the dietary modelling
- PART 2 describes the 'risk factors' (e.g. BMI, sodium). All interventions must influence one or more risk factors, to in turn generate health gains (or losses) and changes in costs.
- PART 3 describes the 'disease' BODE^3 DIET MSLT modelling. Here changes in risk factors lead to changes in disease incidence, and then to changes in health and costs.

[iii] Future models will extend the range of risk factors included, e.g. physical activity, wholegrains, see section 1.01.08 for further details.

Figure 1: Conceptual model for dietary interventions

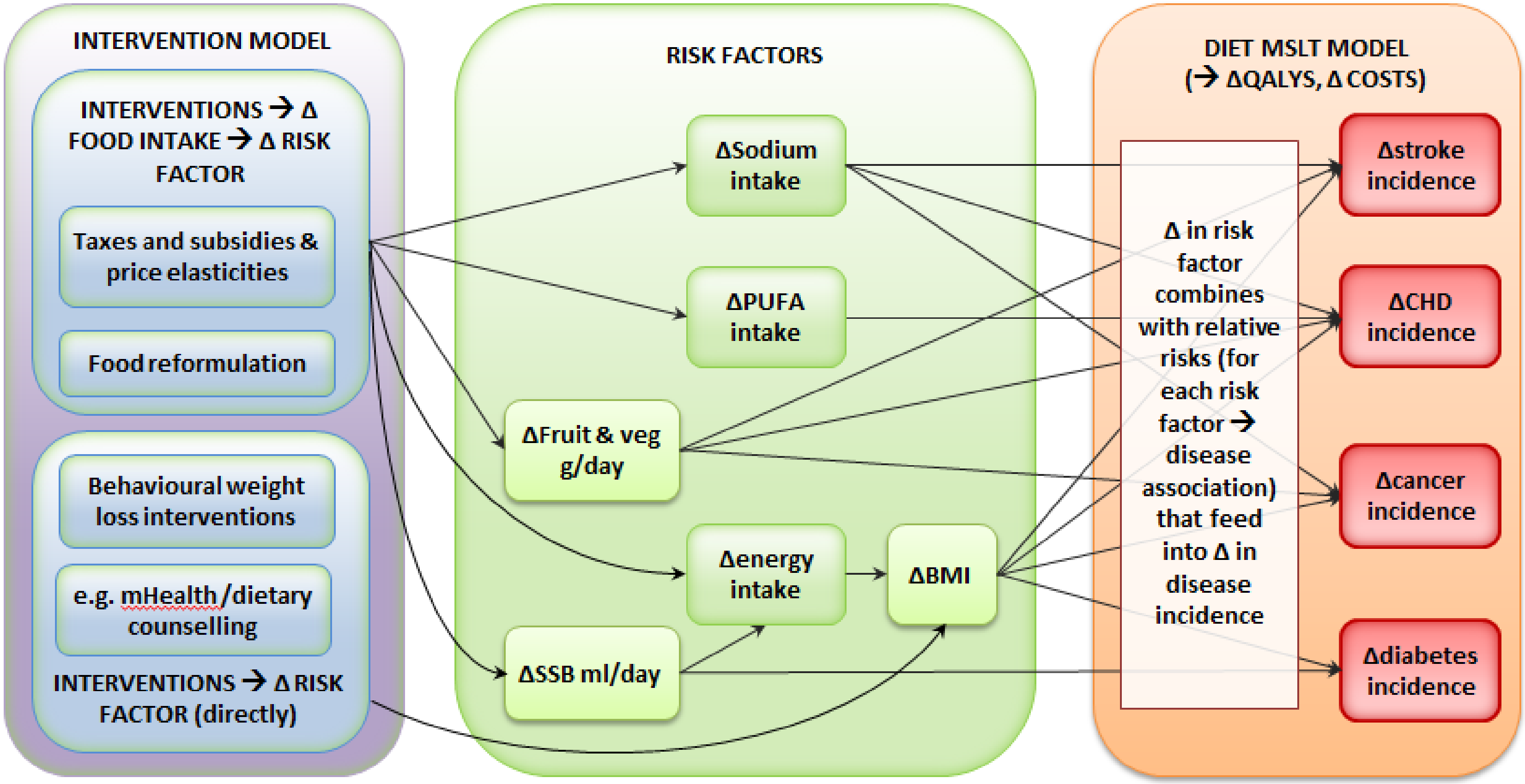

This conceptual model applies as of the time of this first Technical Report, e.g. the risk factors may increase in the future to include e.g. wholegrains.

BMI: Body Mass Index, PUFA: Polyunsaturated fatty acids, SSB: Sugar-sweetened beverages, CHD: Coronary Heart Disease.

PART 1. Dietary Modelling

In PART 1, we first describe some of the fundamental baseline dietary data (e.g. food consumption and price data; Section 1.01). The following two sections consider dietary interventions directly changing a 'risk factor', such as dietary counselling parametrized as directly changing body mass index (BMI; Section 1.02), and dietary interventions that change food consumption or composition (and then change risk factors; Section 1.03).

Section 1.03 includes a detailed section on price elasticities, which are the 'conduit' through which price changes (e.g. taxes and subsidies) change food purchasing and therefore intake. Price elasticities are complex and uncertain, and accordingly this section is detailed (and the reader not so interested in taxes and subsidies may skip this section). This complexity is because a price increase on one food item not only changes its own consumption, but also can change consumption of other food items, i.e. substitutes (e.g. increasing the price of high fat milk would be expected to reduce its consumption, but also increase consumption of other types of milk) and complements (e.g. increase the price of sugar would be expected to reduce its consumption, and possibly also reduce consumption of 'complementary' products which are often consumed with sugar, such as tea).

Section 1.01. Baseline diet data

1.01.1. Food consumption data - baseline

Data from the New Zealand Adult National Nutrition Survey (NZANS)[2] conducted in 2008/09 was used for food consumption data, obtained directly from the University of Otago's Life in New Zealand Research Group who conducted the survey (personal communication, Parnell, 2014). The data are in grams per food group (n=346) for each of 4721 participants (see Appendix A: Food groups from the NZANS used in the BODE[3] intervention model (page 63) for a list of these food groups). Data for each of the 346 food groups was too sparse when stratified, or modelled, by age in addition to sex and ethnicity. Therefore, for the majority of foods we simply calculated a value for each sex by ethnic group, averaged by age. Some of the risk factors modelled vary profoundly by age (e.g. sugar-sweetened beverage (SSB) consumption, which is also a 'risk factor' in our conceptual model in Figure 1). To account for this variation we calculated percentage changes in consumption for the intervention effect and then applied this percentage change to age-specific consumption amounts to give a change in consumption by age. That is, we assumed a constant percentage effect of the interventions on consumption by age, but allowed for heterogeneity in absolute consumption by age.

1.01.2. Food nutrient data

The nutrient content of each of the 346 food groups was calculated using the New Zealand food composition tables, using the matching schema carried out in the NZANS. For each of the 119,037 food items[iv] recorded in the 24-hour recall collected in the NZANS, the Life in New Zealand Activity and Health

[iv] These 119,037 food items were coded into the 346 food groups.

Research Unit (LINZ) nutrition team matched the food item to a food item in the New Zealand food composition tables (unpublished data supplied by Otago University Life in New Zealand staff (personal communication, Blakey, Smith and Parnell, 2014)). The nutrient content of all the food items consumed within each of the 346 food groups was weighted by the grams of each food item consumed over the total number of grams consumed within each food group for the whole survey population.

For example, there were 864 records of consumption of 'White rice (includes parboiled & basmati)' (Food group 01.01.01) in the survey. This was matched to 6 different types of rice in the New Zealand food composition tables. The main food composition item that the 01.01.01 rice was matched to was 'Rice, white, polished, boiled' at 163,305 grams out of a total of 200,694 grams. The food composition data associated with 'Rice, white, polished, boiled' contributed 81% (163,305/200,694) to the food composition data for 01.01.01.[v]

Food composition data is available for energy, protein, total fat, total carbohydrates, sodium, fibre, cholesterol, saturated fat, monounsaturated fat, polyunsaturated fat, fructose, glucose, lactose, maltose, sucrose, sugar, starch, calcium, copper, iodine, iron, magnesium, manganese, phosphorus, potassium, selenium, zinc, beta-carotene, retinol, vitamin A, thiamine, riboflavin, niacin, vitamin B6, folate, vitamin B12, vitamin C, vitamin D, vitamin E, alcohol and caffeine. The nutrients currently used in the BODE[3] Dietary intervention model are energy, total fat, saturated fat, polyunsaturated fat, sodium and sugar. The food composition data were specified per 100 grams, these values were multiplied by the amount consumed in the four demographic groups to give baseline nutrient intakes, which in turn was converted to an intervention nutrient intake.

Sodium excretion data from the NZANS was available and was used to scale the sodium intake data up to the excretion data. This was to account for under reporting in the dietary intake data. The ratio of sodium intake data, calculated as described above, and the urinary excretion data from the NZANS (provided by Dr Rachael McLean from the Human Nutrition Department, University of Otago) were used to calculate scalars that were then applied to the sodium intake data. The scalar for males was 1.339 and for females was 1.51, these were applied to both ethnicities and all age groups.

The difference in nutrient intake from baseline to intervention scenarios modelled is the basis of the risk factor calculations for change in energy intake for the BMI calculation, change in sodium intake and change in total energy from polyunsaturated fat intake.

1.01.3. Price

The retail prices of New Zealand specific Nutritrack supermarket data[3] were used to estimate food prices of the NZANS food groups. The brand name and product name in the Nutritrack database were grouped by Nutritrack food category (for example, beverages or bread products). NZANS food groups and Nutritrack food products are not the same groupings. At maximal disaggregation, there were 346 NZANS food groups, and 6192 Nutritrack food products. Therefore, NZANS food groups and associated food

[v] All relevant files are stored on the BODE[3] shared drive at: G:\Publications\Papers\300_A_DIET MODELS\DIET models\Working documents for model development\Price and nutrient matching, 8 February 2017.

group codes were matched to an assortment of Nutritrack food products to provide price information for each food group. Product price was considered when matching food groups to the corresponding food products to ensure the range of products were most appropriate in terms of cost. Each food group required matching to at least one food product, and where possible food groups were matched to at least 10 different food products.[v]

Where there were a limited number of appropriate Nutritrack food products available to match to NZANS food groups, food products were duplicated to allow more than one NZANS food group to be matched to one Nutritrack product. Food groups that reflected recipes (for example, casseroles/stews with sauce only) were matched to the most appropriate food products resembling the same or similar food components, and with probable similarities in terms of cost.

Prices for food groups that could not be matched to Nutritrack data (collected between December 2010 and April 2011) were obtained from online supermarket data. This included food products such as fresh fruit, vegetables and meat and poultry. The prices for these food products were obtained using the Countdown online supermarket (http://shop.countdown.co.nz). An unweighted average price was calculated across a range of food products considered to be most commonly consumed to obtain an average price for that food. Prices obtained from the online supermarket (year 2014) were scaled using the CPI to reflect 2011 prices.

Section 1.02. Dietary interventions parameterised as directly changing a risk factor

Here we focus on the 'general' modelling of dietary interventions onto 'risk factors' (the risk factors currently in the BODE[3] DIET MSLT model are fruit, vegetables, sugar-sweetened beverages, sodium and polyunsaturated fat intake and BMI). The parameterisation of these interventions is straight forward in principle. Firstly, we need to determine, based on the current best evidence, the effect of the intervention on the particular risk factors. For example, how much does BMI decrease with a mHealth weight loss intervention? To determine the effect size we perform a literature search, and possibly expert knowledge elicitation (see BODE[3] Protocol for a general approach [4], and specific publications for specific approach).

Then, there are a number of other factors to consider and parameterise:

- Who does the intervention effect? (e.g. just obese? Or everyone?)
- Is there any heterogeneity of effect size by population characteristics? (e.g. sex, age)
- What proportion of this population takes up and also completes the intervention?
- What attenuation of effect is there over time? (E.g. informed by a literature search, and probably at least some expert knowledge elicitation (empirical estimates often sparse, and for short follow-up only), to determine the attenuation.)
- Any heterogeneity of attenuation by population characteristics? (e.g. sex, age; however, it is most unlikely that enough information will be available to specify such heterogeneity of attenuation).

This intervention effect size and attenuation, with attendant uncertainty, is then modelled as an absolute change in risk factor (e.g. a 0.2 absolute unit change in BMI), for the intervention population of interest (e.g. all people 65 years and older, all people with a BMI ≥ 30). This intervention effect size is then applied to each relevant category of risk factor (e.g. if the intervention was targeted at people with a BMI over 30, then only to these groups; see PART 2 later for more detail on how this 'feeds into' the population impact fraction (PIF) estimation using a 'relative risk shift method').

Section 1.03. Dietary interventions that change food consumption or composition

For the purposes of this Technical Report, we consider two types of intervention here: price changes from taxes and subsidies (Section 1.03.1); and food reformulation by the food industry (Section 1.03.2). Food taxes and subsidies are complex to model, and dominate this Section.

1.03.1. Food taxes and subsidies

The BODE^3 intervention model, which merges food price changes with price elasticities to generate changes in 346 foods consumed, is complex. The conceptual process is that a change in price of food(s) leads to change in purchasing (and in parallel consumption), modelled through price elasticities (PEs). This change in consumption then leads to percentage changes in food (vegetables, fruit, SSBs) and nutrient (sodium, PUFA) and total energy intake, which in turn changes disease incidence. The most complicated component is the change in food price to change in consumption, through price elasticities, for reasons such as:

- There are many possible foods that can have a price change, yet price elasticities are only (usually) calculated for aggregate groupings of foods.
- For any single food with a price change, one has to not only model its own change in purchasing/consumption (through own-PEs), but also how the change in this food effects consumption in all (or some) other foods (through cross-PEs).
- Price elasticities are calculated as a system in a different context to that in which they are applied in modelling. For example, the starting consumption of foods may differ between the context in which the PEs were calculated, compared to the population to be modelled. For a price set change (especially if large and/or affecting multiple foods; e.g. a saturated fat tax) the predicted purchasing/consumption of many foods changes, and it is possible to see 'implausible' changes in energy intake. Put another way, the PE modelling may 'correctly' see decreases and increases in consumption of foods relative to one another, but the net energy intake change may be implausibly large.

Yet food taxes and subsidies are a key public health research question, and using price elasticities is usually necessary. We address some of these issues in this Technical Report.

This Section is structured as follows:

- Price elasticities:

- Disaggregating price elasticity matrices
- Theoretically selected price elasticities

- Calculating the estimated change in consumption for a given price change
- Constraining total food expenditure change

(i) Price elasticities

In this section we:

1. Outline the method used to disaggregated a 24 by 24 price elasticity matrix (from the SPEND Study[5,6]; Figure 2, page 13) into a 338 by 338 price elasticity matrix. The reasons for this disaggregation is that our price interventions will differentially effect price within each of the 24 aggregated food categories (e.g. a saturated fat tax based on grams of saturated fat per 100g of product would not affect the price of each food sub-type (e.g. low and high fat cheese) by the same percentage within each aggregated food category (e.g. dairy products)). We mainly used theoretical means to do this, as empiric data are limited or non-existent. Price change, per gram of saturated fat, is based on the food composition data of the specific food groups.
2. Outline a basis upon which to theoretically 'set' many cross-PEs to zero for use as either the 'best' or scenario analyses (a choice that will be made in subsequent publications). The reason for doing this is because even a small (but erroneous) price elasticity from, say, dairy to fruit may just add more error to modelling, whereas theoretical setting of some cross-PEs to zero may improve subsequent modelling – or at least provide a useful scenario or sensitivity analysis. Many published modelling studies theoretically suppress cross-PEs (e.g. [6-8]).
3. Outline a method to scale all purchases up or down by the same percentage, after modelling through disaggregated price elasticities. The reason for doing this is that even with our best efforts above to specify PE matrices, one may still end up with an implausible change in total food expenditure (and total energy intake). For example, a 10% increase in average food prices due to a saturated fat tax may result in no change in food expenditure (and a 10% reduction in energy intake) through the above PE matrix modelling. Yet there is an elasticity of total food expenditure given change in total food price; an envelope within which redistribution between foods must operate. There are also reasons from econometric theory why such an envelope is sensible to invoke, namely that if the prices of many foods changes then expenditure on food (in total) now has to also consider the total household budget and income elasticities (e.g., for some budget-constrained families higher food prices may result in total reductions in the amount of food purchased).

Figure 2: Price elasticity table from the SPEND Study for aggregated food groups [6] 24 by 24 matrix (shaded cells show own-PEs, other values are cross-PEs)

	Fruit	Vegetables	Beef, lamb & hogget	Poultry	Pork	Prepared, preserved & processed meat	Fish & seafood	Bread & breakfast cereals	Cakes & biscuits	Pastry cook products	Pasta & other cereal products	Milk, yoghurt & eggs	Cheese & cream	Butter	Margarine & edible oil	Sauces, sugar & condiments	Chocolate, confectionary & snacks	Ice cream	Other grocery food	Non-alcoholic beverages	Carbonated soft drinks	Ready to eat food	Energy drinks
Fruit	-0.58	0.06	-0.05	-0.01	0.04	-0.04	0.01	0.06	-0.17	0.04	-0.05	0.01	0.08	0.05	-0.04	0.04	0.01	0.00	-0.25	0.00	-0.11	-0.21	-0.09
Vegetables	0.05	-0.88	-0.08	-0.04	0.00	-0.02	0.03	-0.03	-0.10	-0.02	0.03	-0.03	0.01	-0.08	0.03	0.06	0.02	0.00	-0.17	0.03	-0.11	-0.04	0.03
Beef, lamb & hogget	-0.07	-0.11	-0.93	0.05	0.01	-0.01	-0.05	-0.11	-0.12	-0.08	-0.02	-0.03	-0.04	0.03	-0.02	-0.05	0.00	-0.02	-0.28	0.04	-0.03	0.05	0.15
Poultry	-0.19	-0.14	0.03	-1.70	0.21	0.11	-0.02	0.08	0.27	0.16	-0.03	0.17	0.02	0.25	-0.01	-0.01	-0.04	-0.04	-0.20	0.05	0.03	0.09	0.21
Pork	-0.13	-0.34	0.05	0.43	-4.51	0.25	-0.23	-0.01	0.35	-0.02	-0.07	0.20	0.03	0.17	0.15	-0.04	0.11	-0.06	0.04	0.12	0.65	0.11	1.95
Prepared, preserved & processed meat	-0.07	-0.07	-0.02	0.12	0.10	-1.05	0.00	-0.09	-0.06	-0.01	0.05	-0.07	0.01	0.11	-0.09	-0.06	-0.09	-0.06	-0.18	-0.01	-0.06	-0.10	0.07
Fish & seafood	-0.02	0.21	-0.16	-0.06	-0.11	0.12	-1.68	0.26	0.03	0.06	-0.09	0.24	0.18	-0.02	0.05	0.03	-0.11	-0.04	-0.22	0.03	-0.23	-0.05	-1.04
Bread & breakfast cereals	0.02	0.01	-0.12	0.05	0.08	-0.05	0.05	-0.73	0.06	-0.02	0.01	-0.15	0.00	0.02	-0.09	0.00	0.07	0.00	-0.14	0.02	0.06	-0.09	0.24
Cakes & biscuits	-0.15	-0.10	-0.06	0.07	0.29	0.00	0.04	-0.05	-0.97	-0.09	0.01	-0.04	-0.05	-0.08	-0.06	-0.01	0.03	-0.03	-0.10	-0.01	0.11	-0.06	0.24
Pastry cook products	-0.05	-0.11	-0.20	0.05	0.33	-0.12	-0.15	-0.47	0.43	-1.52	0.15	-0.40	-0.09	0.55	-0.26	-0.09	-0.19	-0.06	0.32	-0.08	-1.90	0.32	1.23
Pasta & other cereal products	-0.05	0.10	-0.16	0.28	-0.05	0.20	0.08	0.27	-0.07	0.12	-1.70	0.11	0.02	-0.02	0.06	-0.06	-0.12	0.00	0.10	0.07	-0.14	0.25	-0.36
Milk, yoghurt & eggs	-0.02	-0.03	-0.01	0.07	-0.01	-0.07	0.06	-0.14	-0.04	-0.01	-0.01	-0.86	-0.03	0.07	-0.01	-0.02	0.04	0.00	-0.17	0.05	-0.03	-0.04	0.09
Cheese & cream	0.18	0.04	-0.07	-0.01	0.03	0.12	0.06	0.11	-0.10	0.04	0.00	0.00	-1.04	0.28	-0.10	-0.04	-0.20	-0.02	-0.21	0.01	0.04	-0.22	-0.07
Butter	0.03	-0.50	-0.34	-0.40	0.39	0.13	0.06	0.09	-0.35	-0.56	-0.50	-0.03	0.01	-0.67	0.50	-0.57	-0.15	-0.09	-0.49	0.07	1.01	-0.05	0.29
Margarine & edible oil	-0.27	-0.05	0.02	0.15	0.12	0.00	0.04	-0.32	-0.27	0.03	-0.04	0.00	-0.07	0.43	-1.04	-0.08	-0.13	0.01	-0.16	0.06	-0.47	0.04	-0.62
Sauces, sugar & condiments	0.13	0.11	-0.18	0.15	-0.01	-0.01	0.05	-0.04	-0.15	0.05	-0.06	0.16	-0.11	0.10	0.05	-1.32	-0.21	-0.04	-0.12	0.03	0.01	0.06	-0.22
Chocolate, confectionary & snacks	0.12	0.02	-0.02	0.03	0.18	0.01	-0.01	0.21	0.04	-0.01	0.04	0.08	-0.08	0.12	-0.02	-0.05	-1.27	-0.08	-0.05	-0.08	0.31	0.07	0.27
Ice cream	0.19	0.19	-0.13	0.55	-0.01	-0.10	-0.05	0.11	0.22	-0.11	0.08	0.01	-0.03	-0.03	0.02	0.01	-0.09	-1.74	0.24	-0.06	0.20	0.38	-0.47
Other grocery food	-0.09	-0.12	-0.09	-0.08	0.01	-0.07	0.00	-0.10	-0.04	-0.04	0.03	-0.10	-0.01	-0.07	0.02	0.03	0.04	0.05	-0.38	0.03	0.07	-0.09	-0.12
Non-alcoholic beverages	-0.10	-0.04	0.00	0.05	0.11	0.02	0.04	0.03	-0.17	-0.03	-0.03	0.11	0.09	0.01	-0.02	-0.10	-0.12	-0.01	-0.13	-1.31	0.26	-0.25	-0.42
Carbonated soft drinks	-0.14	-0.27	0.23	0.59	0.06	0.17	-0.14	-0.21	0.69	-0.25	0.13	0.11	-0.02	0.67	-0.24	-0.02	-0.01	0.03	0.15	-0.18	-1.23	0.05	0.77
Ready to eat food	0.03	0.06	0.15	0.08	0.13	0.03	-0.01	0.11	0.08	0.12	0.10	-0.06	0.03	0.02	-0.01	0.01	0.00	0.05	0.04	-0.08	0.10	-0.93	0.15
Energy drinks	-1.14	0.39	0.36	0.18	1.78	-0.08	-0.23	0.32	3.18	-0.06	0.19	0.25	-0.40	0.49	-0.25	-0.35	-0.58	-0.07	0.31	-0.71	2.73	0.10	-0.31

See Appendix B: SPEND Study price elasticity tables(page 72) for Standard Errors of these Price Elasticities

(ii) Generating disaggregated PE matrices

Initial price elasticities were from the SPEND Study, conducted for New Zealand.[5,6] These are in a 24 by 24 matrix (see Figure 2, page12) of own- and cross-PEs (with standard errors for default uncertainty). These 24 food groups have been matched to the 346 food groups used in the intervention model. This gives us 24 overall food groups and 338 food subgroups (ignoring 5 'alcoholic beverage' groups, 2 'dietary supplement' groups and 1 'not applicable' group). The 24 by 24 price elasticity matrix was then expanded to a 338 by 338 matrix as follows:

- *Own-PEs:* Econometric theory posits that as one keeps disaggregating foods into smaller and smaller subgroupings, the own-PE of each food is expected to increase (in absolute value terms).[9-12] For example, the own-PE of all bread might be -0.5, but wholegrain bread separated might be -0.55. Why? Because, assuming subgroups in each aggregated category are substitutes, changing the price of just white bread means consumers can swap to multigrain bread, meaning that consumers can be more price sensitive (a larger, in the negative sense, own-PE). How much does the own-PE strengthen? Unfortunately, that is difficult to estimate. What we have done is assumed that the own-PE increases by 2.5% (with wide uncertainty expressed as a 50% (of 2.5% = 1.25 percentage point) standard deviation (SD) on the normal scale) for each additional food sub-group. Of note, the own-PE increases by 5% if splitting one category in two (we deliberately allow a greater increase in own-PE for the first split), but then 2.5% for each additional food category thereafter. (Whilst theoretical literature can be found to support the fact that own-PE increases with disaggregation,[9-12] we were unable to find empirical research on the same for food. We therefore plan to undertake such analyses ourselves in the future with data collected from a virtual supermarket experiment in the Price ExaM study (within the DIET Programme; https://diet.auckland.ac.nz/content/price-exam) for which we can change the level of food disaggregation in calculating own-PEs – and then amend our 2.5% estimate as appropriate.) The overall sensitivity of the modelling to this parameter will be investigated and reported with one-way uncertainty analyses and Tornado plots (e.g. of QALYs gained).

- *Cross-PEs within the initial food group.* We assume that each food subgroup (e.g. four bread sub-types of white bread, fibre-containing white bread, wholemeal bread, wholegrain) within each separate food (e.g. bread) is a substitute for each other, meaning they have small positive cross-PEs. We specify all these, so the sum (across rows of PE matrix) of own- and cross-PEs gives the SPEND Study's own-PE, following econometric theory.[12,13] For example, if as above the own-PE of breads as one aggregated food category was -0.5, but when disaggregated the four sub-categories of bread each had an own PE of -0.55 then the sum of:

    - Wholegrain bread's own-PE (-0.55)
    - and each of the three cross-PEs of white, fibre white, and wholemeal onto wholegrain

    … must be -0.5. Meaning the sum of the three cross-PEs must be +0.05. We disaggregated this quantum across the three non-wholegrain breads proportional to their consumption (i.e. the cross-PE of a commonly purchased item on x is greater than the cross-PE of a rarely purchased

item on x). For example, assume that the percentage consumption of the three non-wholegrain breads was white=50%, fibre white=20%, and wholemeal=30%, then the cross-PEs for white onto wholegrain would be 50%×0.05 = +0.025, for fibre white = 0.01, and for wholemeal = 0.015.

Note that, thus far, we have two main assumptions: first, that own-PEs increase by 2.5% (with wide uncertainty) for each additional sub-category of food; second, that the disaggregation of cross-PEs is proportionate to that food's relative consumption. These assumptions are qualitatively justified based on econometric theory, but the exact quantification (or weighting) is unknown, and needs empirical testing (and in the meantime uncertainty or scenario analyses).[vi]

- *Cross-PEs for food sub-categories of food in different aggregate categories.* (For example, for each of four breads (e.g. white, fibre white, wholemeal and wholegrain) onto any fruit.) Again, the cross-PE from the aggregated categories needs to be disaggregated by food sub-category, and we assume weighted by consumption. So, extending the above example, the cross-PE of *aggregated* bread onto fruit is 0.016 from the SPEND PE matrix (Figure 2, page 13). Assume wholegrains were 20% of all bread expenditure (the percentages above excluded wholegrain), meaning percentage expenditure on the three other breads within all breads is white=80%×50%=40%, fibre white=80%×20%=16%, and wholemeal=80%×30%=24%. And therefore, the cross-PEs for each of these four breads onto (any) fruit is estimated to be white=40%×0.16=0.064, fibre white =11%×0.16=0.026, wholemeal=24%×0.16=0.038, and wholegrain=20%×0.16=0.032.

Logic checking of the above was undertaken by determining changes in purchasing for various policies that had the same percentage price change on all food subtypes (e.g. all sub-types of bread and cereals) within each aggregate food category (e.g. bread and cereals combined) through the completely disaggregated and the 'simple' aggregated price elasticity matrix – identical results were obtained, as should be the case.

[vi] Assumptions implicit to price elasticity matrices include:
- The homogeneity assumption: the sum of the cross-PEs for a product and the income elasticity for that product is zero.
- The budget constraints assumption: the sum of the income elasticities weighted by the share of income spent on the goods is equal to 1.

Further mathematical work by Scarborough and Blakely managed to meet this 'stricter' homogeneity assumption using an 'odds' method to calculate the cross-PE in this system (further information from authors; emails and workings August 2016). However: 1) whilst 'mathematically correct' for one system of disaggregated foods, implausibly high cross-PEs can result; 2) it was mathematically intractable to find a solution of linear equations to apply to a larger food system (as we need to in the BODE[3] intervention model).
We also note that the application of PE matrices calculated in one setting (with a set of assumptions (e.g. conditionality, meaning no change in budget share for food)) to another setting (e.g. New Zealand in the future with different starting distributions of food consumption, tastes and preferences) is structurally uncertain – albeit unavoidable.
Therefore, in the interests of model parsimony, we settled on the approach here detailed in the main text of this Appendix.

(iii) When empirical data on disaggregated PEs exists from other research

Finally, for soft drinks there were actual estimates of cross-PEs for regular and diet soft drinks available through a paper published by Sharma et al (2014)[14] (Australian study). These were rescaled to the SPEND carbonated beverages own-PE as follows:

a) Assume that the relative distributions of own/cross-PEs in Sharma et al apply to New Zealand.
b) Then imagine that diet and regular soft drinks have the same price increase/decrease, meaning that this 2 by 2 matrix should return what the single own-PE in SPEND returns.
c) The SPEND own-PE is -1.23. Thus, we need to make the Sharma 2 by 2 matrix behave as if it were -1.23 in aggregate. We achieved this by a scalar based on budget share (using food consumption from the NZANS as a proxy).

The scalar is calculated as follows. First, the own-PEs (shaded cells) and cross-PEs from Sharma et al (2014) are:

	Regular	Diet
Regular	-0.63	0.16
Diet	0.28	-1.01

Second, the proportionate split of New Zealand consumption of carbonated soft drink consumption from the NZANS [2] was 83.2% regular and 16.8% diet.

Third, the scalar was calculated as follows:

- The aggregate price elasticity for diet and regular soft drinks combined using the (unscaled) Sharma PE matrix combined with the NZANS consumption data is as follows:
  - Component 1: The impact of an increase in **regular** soft drinks will be (0.832 × -0.63) + (0.168 × 0.28). Note the weighting of 16.8% for the second part of this component, as this is the price change in regular impacting the diet drinks – which are 16.8% of consumption.
  - Component 2: The impact of an increase in **diet** soft drinks will be (0.832 × 0.16) + (0.168 × -1.01)
  - The sum of these two components is -0.514.
- The SPEND PE of -1.23 is then divided by the sum of these two components, to give the scalar to apply to each of the Sharma et al PEs such that the SPEND own-PE is preserved in aggregate, i.e. -1.23/-0.514 = 2.395.

Thus, the disaggregated PEs to use in the BODE[3] intervention model were:

	Regular	Diet
Regular	-1.509	0.383
Diet	0.670	-2.418

To check this works, imagine a small 1% price increase in both regular and diet soft drinks. This should return a 1.23% decrease in consumption given the SPEND own-PE of -1.23 for regular and soft drinks combined. Using the disaggregated PE matrix, the 1% price increase in:

- regular soft drinks will give a 1.509% reduction in regular and a 0.670% increase in diet soft drinks. Given that regular soft drinks make up 83.2% of consumption, and diet ones 16.8%, the net change in soft drink consumption (due to change in regular prices only) will be (83.2% × -1.509%) + (16.8% × 0.670%) = -1.143%
- diet soft drinks will give a 0.383% increase in regular and a -2.418% decrease in diet, and therefore a net change in soft drink consumption (due to change in diet prices only) of (83.2% × 0.383%) + (16.8% × -2.418%) = -0.088%
- and, therefore, in both regular and diet soft drinks there will be a net change of: -1.143% + -0.088% = -1.23%, consistent with the 'starting' SPEND own-PE of -1.23.

This disaggregation was repeated using own- and cross-PE from a report published by Tiffin et al in 2011[15] in a sensitivity analysis.

Selected examples of expected (i.e. no uncertainty propagated through calculations) cross- and own-PEs for some of the food sub-types from the fully disaggregated PE matrix are shown in Table 1 below (using methods 1, 2 and 3 above for everything except the underlined block of disaggregated soft drink PEs which uses method 4 above, for Sharma et al (2014) external data), and can be contrasted with the more aggregated SPEND PEs shown in Figure 2, page 12.

Table 1. Selected examples of own- and cross-PE extracted from the 338 by 338 disaggregated price elasticity matrix used in the BODE[3] intervention model. Own-PE are shown in bold, and blocks of disaggregated foods in shaded grey.

**NZANS food groups:**		Butter	Butter/margarine blends	Butter - reduced fat	Regular soft drinks	Diet soft drinks	Energy drinks	Ice cream-regular	Ice cream-rich	Ice cream-reduced fat
	**SPEND PE food groups:**	Butter	Butter	Butter	Carbonated soft drinks	Carbonated soft drinks	Energy drinks	Ice cream	Ice cream	Ice cream
Butter	Butter	**-0.720**	0.050	0.000	0.889	0.121	0.290	-0.043	-0.004	-0.012
Butter/margarine blends	Butter	0.050	**-0.720**	0.000	0.889	0.121	0.290	-0.043	-0.004	-0.012
Butter - reduced fat	Butter	0.045	0.006	**-0.720**	0.889	0.121	0.290	-0.043	-0.004	-0.012
Regular soft drinks	Carbonated soft drinks	0.596	0.074	0.000	**-1.922**	0.488	0.770	0.014	0.001	0.004
Diet soft drinks	Carbonated soft drinks	0.596	0.074	0.000	0.854	**-3.081**	0.770	0.014	0.001	0.004
Energy drinks	Energy drinks	0.436	0.054	0.000	2.402	0.328	**-0.310**	-0.033	-0.003	-0.009
Ice cream-regular	Ice cream	-0.027	-0.003	0.000	0.176	0.024	-0.470	**-1.958**	0.018	0.054
Ice cream-rich	Ice cream	-0.027	-0.003	0.000	0.176	0.024	-0.470	0.109	**-1.958**	0.029
Ice cream-reduced fat	Ice cream	-0.027	-0.003	0.000	0.176	0.024	-0.470	0.119	0.011	**-1.958**

(iv) Theoretically selected cross-PEs

We updated the literature review from our previous work [16] to include PE studies for high-income countries (mainly UK, US and Australia). We searched Ovid database with the keywords: "Price elasticit$" AND "Food$" OR "Drink$" OR "Beverage$", NOT tobacco, NOT alcohol, from 2000 onwards (English language, human, full text). Studies that just estimated price elasticities in low or middle income countries were ignored. These studies had to report cross-PEs between at least two food groups (given we are interested in cross-PEs). There were 11 studies that meet our search criteria.[9,17-26]

We matched food groups from the selected studies with the BODE[3] intervention model's food groups. Then all PEs from these studies were extracted to a database. Median cross-PEs from this database were selected as the best cross-PE for each food group pairing in the PE matrix (There were some outliers in the data so we decided not to use average cross-PEs, the majority of the cross-PE had three or more estimates). We refer to these selected cross-PEs as the BODE[3] cross-PEs (as opposed to the SPEND cross-PEs). We also classified cross-PEs as a weak, medium or strong association. That is:

$$|\boldsymbol{cPE}| \begin{cases} \leq \mathbf{0.04}: \boldsymbol{weak} \\ > \mathbf{0.04} \cup \leq \mathbf{0.09}: \boldsymbol{medium} \\ > \mathbf{0.09}: \boldsymbol{strong} \end{cases} \qquad (1)$$

These values were estimated from our PE database above, with weak association accounting for the lower 25th percentile, strong association for the upper 25th percentile, and medium association being the rest.

For modelling of the impact of price changes on food purchasing/consumption, we will use three general approaches, each with alternative options or scenarios within it:

Approach A: use SPEND PEs

In this approach we will simply use all SPEND own-PEs and cross-PEs (i.e. no suppression of any cross-PEs, use standard errors about each own-PE and cross-PE as initial uncertainty intervals to draw from in Monte Carlo simulation).

Suppress selected cross-PEs as sensitivity analyses:

- suppress (i.e. set to 0) those SPEND cross-PEs that in the above mentioned literature review we classified as 'weak', i.e. where the BODE[3] |cross-PE| ≤ 0.04(AS1, see Appendix B: SPEND Study price elasticity tables, page 72);
- suppress those SPEND cross-PEs that in the above literature review we classified as 'weak' or 'moderate', i.e. where the BODE[3] |cross-PE| ≤ 0.09 (AS2, see Appendix B: SPEND Study price elasticity tables, page 72).
- suppress those SPEND cross-PEs as 'theoretically' determined by previous users [6,8] of SPEND price elasticities (AS3, varied by policy and will be described in detail if used).

Approach B: use BODE[3] (cross) PEs

In this approach we will retain SPEND own-PEs, but use the median BODE[3] cross-PEs from the literature (BS1).

Suppress selected cross-PEs as sensitivity analyses:

- suppress (i.e. set to 0) those BODE[3] cross-PEs that in the above literature review we classified as ‘weak’, i.e. where the BODE[3] |cross-PE| ≤ 0.04 (BS2, see Appendix B: SPEND Study price elasticity tables, page 72);
- suppress those BODE[3] cross-PEs that in the above literature review we classified as ‘weak’ or ‘moderate’, i.e. where the BODE[3] |cross-PE| ≤ 0.09 (BS3, see Appendix B: SPEND Study price elasticity tables, page 72).
- Additional sensitivity analysis: Use the median BODE[3] own and cross-PEs from the literature (BS4, see Appendix B: SPEND Study price elasticity tables, page 72).

All the above Approaches used the above described disaggregation method (page 13) to move from the SPEND 24 by 24 matrix to the fully disaggregated 338 by 338 matrix.

(v) Calculating change in consumption for a give price change

Whilst the matrices are large, and there is uncertainty in the own- and cross-PEs (that is uncertainty intervals about each own- and cross-PE that are sampled from during Monte Carlo simulation), the actual mechanics of calculating the change in consumption is fairly straight forward. Imagine that there are only three food groups, A, B and C

Next, assume that the PE matrix is as follows:

**Food groups**	**A**	**B**	**C**
**A**	-0.7	+0.1	+0.05
**B**	+0.02	-0.5	+0.02
**C**	+0.15	+0.03	-0.9

This means that for each 1% increase in price of A, consumption of A will reduce by 0.7% (own-PEs, shaded), but consumption of B will increase 0.02% and consumption of C will increase by 0.15% (cross-PEs). And so on.

Assume that A has a 20% increase in price, B a 10% increase in price, and C no change in price. Next, assume that initial consumption of A was 500g, B 200g and C 100g. Then the post price change consumption will be:

A = 500g + [500g × (20%×-0.7 + 10%×0.1 + 0%×0.05)] = 435g

B = 200g + [200g × (20%×0.02 + 10%×-0.5 + 0%×0.02)] = 191g

C = 100g + [100g × (20%×0.15 + 10%×0.03 + 0%×-0.9)] = 103g.

This gives change in grams. Whilst we are using consumption data in grams, not purchasing data in grams, as long as one assumes that wastage (i.e. the percent of food purchased that is not consumed) is similar between baseline and intervention, one can safely convert to percentage change after working with grams in the actual calculations. We acknowledge that this is a simplifying assumption about wastage).

(vi) Constraining total food expenditure change

The price elasticities used in this model were calculated from a subset of the New Zealand population, with internationally sourced cross-PEs for scenarios BS1 to BS3 and internationally sourced own and cross-PEs for scenario BS4, and do not 'fit' perfectly to the consumption data from the NZANS used in this model. Moreover, the price elasticity values we use are from 'conditional' models, where the total expenditure on food is assumed fixed; if the interventions we model substantially change prices and therefore overall expenditure on food, we need to allow for how much total food expenditure changes as a result of price changes. These two problems can lead to implausible changes in food expenditure and energy intake if the price elasticities are naively used without constraints.

To address this issue, we need to consider how total food expenditure changes as a result of substantive changes in food prices. That is, we need price elasticities from studies that consider all food together as one category, compared to other household categories like education, housing, clothing, etc. The unconditional (i.e. all household expenditure included) and uncompensated (i.e. real income of the household is assumed fixed) own-PE for food can then be converted to an expenditure elasticity for food, what we call the Total Food Expenditure elasticity (TFE_e):

$$TFE_e = 1 + \text{Unconditional uncompensated own-PE}$$

For example, if the unconditional and uncompensated food own-PE in a study of all household expenditure was -0.3 (i.e. for every 1% increase in average food prices – often called the Food Price Index – consumption of food reduces by 0.3%), then this also means that total food expenditure increases by 0.7% as well (with other household expenditure elsewhere decreased commensurately to compensate).

We are aware of two estimates of such a food own-PE in New Zealand: Michelini and Chatterjee (1997)[27] and Michelini (1999) [27,28] (Table 2, page 23) – unfortunately both are quite old. Nevertheless, of these two studies the Michelini (1999) is probably the best with a longer series of data, the use of an almost ideal demands system (AIDS) model, and more disaggregation of food groups. (It still has limitations, for example the use of repeated cross sectional surveys (Household Economic Survey) for expenditure data and separate official statistics on price indices for various categories of household goods.) Table 2 of this paper reports an own-PE for food combined of -0.168 (standard error 0.1952), which equates to a TFE_e of 0.832 (with the same standard error, which translates to a 95% confidence interval of 0.45 to 1.21).

Theoretically, we would not expect the TFE_e to exceed 1.0. If it did exceed 1.0, this would suggest that as food prices increased expenditure increased even faster – clearly implausible on a fixed household total budget. Conversely, it seems unlikely that the TFE_e is less than 0, as food is essential

to our existence. Accordingly, the naïve upper confidence limit of 1.21 from the Michelini (1999) derived TFE seems implausible – it should be less than 1.0.

Table 2 (page 22) presents TFE_e estimates for eight studies that used multi-stage budgeting models to estimate unconditional and uncompensated food own-PEs, for high-income countries up to June 2017 (keywords: “price elasticities” or “price elasticity” or “demand” and “food” and “multi-stage” or “multi stage”, mainly Google Scholar) Consistent with theoretical expectation, all estimates were between zero and one – albeit spanning this entire range. The previous New Zealand study estimated a TFE_e of 0.68, a bit less than 0.832. The average, median and standard deviation across these eight studies were 0.59, 0.66 and 0.29, respectively. In the absence of an ideal (let alone perfect) recent New Zealand study, we elected to specify a Beta distribution to estimate the TFE_e, a Beta distribution was chosen as the value needs to be between 0 and 1. Values for alpha and beta were varied in order to return a mean of close to the New Zealand literature and were set to 6 and 2. This returns a mean of 0.75, a median of 0.77, a mode of 0.83, a 2.5th percentile of 0.42 and a 97.5th percentile of 0.96 (Figure 3). This distribution captures the two previous New Zealand estimates well, and captures the range of other studies reasonably well (without being overly influenced by the apparent ‘outlier’ Banks et al (1997) estimate.

Figure 3: Beta distribution used for TFE_e (source: Ersatz add-in to Excel)

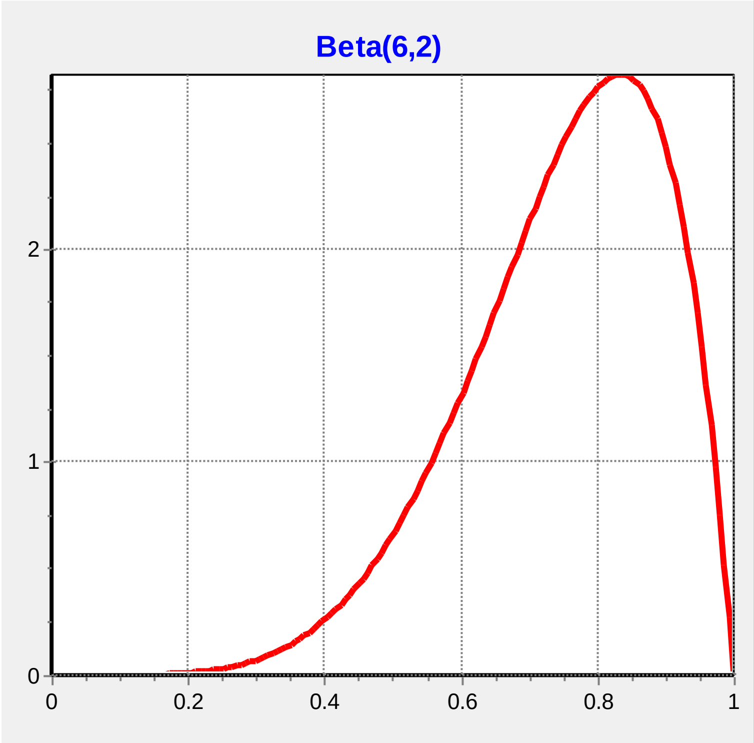

Table 2: Description of the studies used to generate TFE_e values used in the BODE[3] intervention model (ordered by increasing TFE_e; standard errors when published are in the parentheses)

Study	TFE_e =1+ PE for food	Country	Data	Category of economic "goods"	Demand model
Banks et al 1997 [29]	0.041 (0.15)	UK	Using data from the UK Family Expenditure Survey, repeated cross sections for the period of 1970–1986	Food, fuel, clothing, alcohol, and others	QUAIDS
Klonaris and Hallam 2003 [30]	0.457	Greece	Annual time series data from the National Accounts of Greece for the period 1959–1995 on food and non-food consumption expenditures	Food, clothing, housing, transportation, education and health cares	AIDS three-stage budgeting
Huang and Huang 2011 [31]	0.537	US	Annual time series data covering 1960 to 2006 from household expenditure survey.	Foods, energy, clothing, transportation, furniture and health cares	A differential demand system
Brannlund et al. 2007 [32]	0.66	Sweden	Annual time series data on Swedish consumption of non-durable goods.	Food, transport, heating, and other non-durable goods	AIDS three-stage budgeting
Michelini 1999 [28]	0.832 (0.19)	New Zealand	Household Expenditure and Income Surveys (New Zealand Department of Statistics, 1992), for the years 1983/1984 to 1991/1993	Food, household operations, apparel, transport, other goods, and other services	AIDS model
Michelini and Chatterjee 1997 [27]	0.68	New Zealand	Household Expenditure and Income Surveys (New Zealand Department of Statistics, 1992), for the years 1983/1984 to 1991/1992	Food, household operations, apparel, and transport	The Restricted Non-Linear Preference System
Aepli 2014 [33]	0.898	Switzerland	Six years of repeated cross-sectional data from the Swiss Household Expenditure Survey of almost 20,000 households.	Food, tobacco, beverages, and other goods and products	QUAIDS three-stage budgeting

This TFE_e estimate was used to 'set' the new expenditure on food, i.e. [baseline expenditure] × (1+ [% change in food price index] × TFE_e).

There was one additional prior step required too. Changing total household expenditure on food is equivalent to an income change for food consumption. Therefore, income elasticities for each food category were also applied. This step made little relative difference to food expenditure, and everything was still scaled to the 'set' new expenditure based on the TFE_e and percentage change in food price index.

In summary, given our (necessary) reliance on: a) less than ideal price elasticity matrices; b) baseline food consumption distribution in our simulation studies that are not the same as that used in price elasticity estimation and; c) simulated food price interventions that will change the food price index by more than a trivial amount, it was necessary to 'set' the new total expenditure on food. To not do so would have risked implausible changes in total food expenditure and – importantly for final estimation of health gains – implausible changes in food energy intake. We specify generous uncertainty about the TFE_e, as it is genuinely uncertain. Finally, the TFE_e essentially just scales all food purchasing up or down by the same amount; the relative impact on food consumption from the PE matrix is preserved (e.g. the effect of a saturated fat tax decreasing fatty food purchasing but increasing non-fatty food purchasing, *relative to each other*, is preserved).

1.03.2. Food reformulation

The methods used for food reformulation will be expanded in future versions of this Technical Report. In principle, the approach will be:

1. Specification of the policy option, and what foods/nutrients it targets.
2. Estimation of how much individual food product, or nutrient amounts directly, change as a result of the policy. This will be fed into the foods, and resultant changes in risk factors, from baseline, will be estimated. These are likely to be for nutrient risk factors and BMI only (i.e. for sodium, PUFA and BMI).

PART 2. Risk Factors

Section 2.01. Risk factor distributions

There are currently six risk factors generated in the BODE^3 intervention model that flow into the BODE^3 DIET MSLT model; change in **BMI**, intake of **fruit** (grams/day), **vegetables** (grams/day), **sugar-sweetened beverages** (SSBs, mls/day), **sodium** (mgs/day) and **polyunsaturated fat** (as a percentage of total energy (%TE)) between baseline intake and intervention intake.

Changes in consumption from baseline to intervention are calculated separately for Māori and non-Māori, males and females, but due to data limitations could not usually be further calculated by age-groups. We treat this (necessary) simplification as satisfactory for estimating average changes across ages, and from there the percentage change (of baseline intake). But given that there are some important age variations in risk factor distributions (e.g. SSBs more commonly consumed by young people), it was necessary to use the 'all ages percentage change' to in turn estimate grams or mls change by age.

This percentage difference is applied to the average consumption for the specific age-groups (15-25, 25-35, 35-45, 45-55, 55-65, 65-75, 75-85 and 85+) giving a change in intake in grams (for fruit and vegetables) or mls (for SSBs) specific to each sex, ethnic and age-group. Change in sodium uses the change in grams for all the different food groups and the sodium content of these foods (outlined in Section 1.01.1) to calculate a change in mg of sodium. This is also calculated by sex and ethnic groups and estimated as above for age groups. The percentage of total energy (%TE) from polyunsaturated fat is calculated for baseline and intervention. The change in %TE from polyunsaturated fat is the risk factor that flows through to the BODE^3 DIET MSLT model and is not differentiated by age-group.

Table 3 (page 25) shows an example of the output generated from a dietary intervention, in this case a 10% SSB tax, using SPEND PEs, and no suppression of cross-PEs (i.e. see page 18, although it is only the SSB own-PE and cross-PEs activated here). This output then flows through to the BODE^3 DIET MSLT model. For example, for Māori males the price change model estimated a 13.99% decrease in SSB purchasing – or 30.45 g per day decrease *averaged* across all ages (see column 5 of Table 3, page 25). We applied the estimated percentage change to the grams per day by age-group (within Māori males) given by the NZANS [2]. Accordingly, absolute consumption of SSBs was estimated to decrease (under the 10% SSB tax intervention) by a minimum of 2.35mls per day for the elderly, and a maximum of 54.54mls per day for young Māori males.

Table 3: Examples of the output of the BODE[3] intervention model used in the BODE[3] DIET MSLT model arising from a 10% SSB tax

		Δ Fruit (g/day)	ΔVegetables (g/day)	Δ SSB (ml/day)	Δ BMI	Δ Sodium (g/day)	Δ PUFA (% total energy)
Māori males	***absolute Δ****	**-0.45**	**-0.43**	**-30.45**	**-0.13**	**0.00**	**0.02%**
	%Δ **	**-0.40%**	**-0.40%**	**-13.99%**	**-0.45%**	**0.00%**	
	15-24	-0.41	-0.25	-54.54	-0.12	0.00	0.02%
	25-34	-0.40	-0.42	-37.62	-0.13	0.00	0.02%
	35-44	-0.43	-0.44	-23.19	-0.14	0.00	0.02%
	45-54	-0.58	-0.48	-11.85	-0.14	0.00	0.02%
	55-64	-0.55	-0.52	-12.15	-0.14	0.00	0.02%
	65-74	-0.50	-0.59	-6.47	-0.14	0.00	0.02%
	75-84	-0.49	-0.49	-6.48	-0.14	0.00	0.02%
	85+	-0.65	-0.46	-2.34	-0.13	0.00	0.02%
Māori females	***absolute Δ****	**-1.07**	**-0.80**	**-16.56**	**-0.07**	**0.00**	**0.01%**
	%Δ **	**-0.72%**	**-0.72%**	**-11.68%**	**-0.25%**	**0.00%**	
	15-24	-0.94	-0.51	-31.09	-0.07	0.00	0.01%
	25-34	-0.93	-0.82	-21.44	-0.07	0.00	0.01%
	35-44	-0.98	-0.86	-13.22	-0.08	0.00	0.01%
	45-54	-1.25	-0.92	-6.75	-0.08	0.00	0.01%
	55-64	-1.21	-1.00	-6.92	-0.08	0.00	0.01%
	65-74	-1.11	-1.12	-3.69	-0.08	0.00	0.01%
	75-84	-1.10	-0.94	-3.70	-0.08	0.00	0.01%
	85+	-1.37	-0.88	-1.33	-0.07	0.00	0.01%
Non-Māori males	***absolute Δ****	**-1.13**	**-1.17**	**-17.11**	**-0.08**	**0.01**	**0.01%**
	%Δ **	**-0.75%**	**-0.75%**	**-12.59%**	**-0.30%**	**0.00%**	
	15-24	-0.92	-0.83	-29.13	-0.07	0.01	0.01%
	25-34	-0.91	-1.15	-20.09	-0.08	0.01	0.01%
	35-44	-0.96	-1.19	-12.39	-0.08	0.01	0.01%
	45-54	-1.24	-1.25	-6.33	-0.08	0.01	0.01%
	55-64	-1.20	-1.33	-6.49	-0.09	0.01	0.01%
	65-74	-1.09	-1.46	-3.46	-0.08	0.01	0.01%
	75-84	-1.08	-1.27	-3.46	-0.08	0.01	0.01%
	85+	-1.37	-1.22	-1.25	-0.08	0.01	0.01%
Non-Māori females	***absolute Δ****	**-1.42**	**-1.48**	**-7.60**	**-0.05**	**0.00**	**0.00%**
	%Δ **	**-0.91%**	**-0.91%**	**-12.48%**	**-0.18%**	**0.00%**	
	15-24	-1.36	-1.06	-19.71	-0.04	0.01	0.00%
	25-34	-1.34	-1.45	-13.60	-0.05	0.01	0.00%
	35-44	-1.41	-1.50	-8.38	-0.05	0.01	0.00%
	45-54	-1.74	-1.58	-4.28	-0.05	0.00	0.00%
	55-64	-1.69	-1.67	-4.39	-0.05	0.00	0.00%
	65-74	-1.57	-1.82	-2.34	-0.05	0.00	0.00%
	75-84	-1.55	-1.59	-2.34	-0.05	0.00	0.00%
	85+	-1.90	-1.53	-0.84	-0.05	0.00	0.00%

*As a result of the intervention (with TFE_e switched on) average intake (for the four demographic groups as a whole) changed by this absolute amount.
**The absolute change was converted to a percentage change that was then applied to the baseline intake of the specific age-groups to give an estimate of absolute change by age.
For all risk factors except BMI the change occurs in the first year, for BMI it takes 2 years for the full BMI change to occur (see section 2.01.1 for details). For taxes and subsidies the change in risk factor is then maintained for the length of the tax/subsidy. For one off interventions the initial effect starts to decay after the first year (or 2 in the case of BMI, see section 1.01.08 for details).

2.01.1. Change in BMI

Change in BMI is calculated through a change in energy intake from baseline to intervention. As outlined in the Nutrients section on page 7, baseline consumption is matched to the energy content of the foods consumed. As consumption increases or decreases so does the energy intake.

Change in energy intake is converted to change in kg and change in BMI using the formula presented in Hall et al (2011).[34] This paper critiques the commonly used 'static weight-loss rule': reduction of food intake of 2mJ/day will lead to a steady rate of weight loss of 0.5kg/week. This Hall et al method takes into account the dynamic physiological adaptations that occur with decreased bodyweight, and quantifies the effect of energy imbalance on bodyweight using mathematical modelling: reduction of food intake of 100kJ/day will lead to a change of 1kg with half of the weight change reached in 1 year and 95% by year 3. This is operationalised in the BODE[3] DIET MSLT model as 50% of the change in BMI in the first year, then 100% of the change by the second year, and then with subsequent weight change either held constant or decayed (due to decaying intervention effect) over time.

PART 3. Disease Modelling

Part 3, Disease Modelling in the BODE[3] DIET MSLT model, is presented as four sections:

1. Section 3.01 outlines the structure of the BODE[3] DIET MSLT model.
2. Section 3.02 outlines the baseline specification and parametrization of the model. In other words, how the mortality, morbidity and cost parameters are expected to behave under 'business as usual' (BAU).
3. Section 3.03 presents model calibration.
4. Section 3.04 presents model validation.
5. Section 3.05 briefly outlines analysis.
6. Section 3.06 provides an additional note on why we use disability-adjusted life-years (DALYs) and QALYs interchangeability in the context of simulation modelling.

Section 3.01. Model structure

3.01.1. Life-table analysis

Life-tables are at the centre of the BODE[3] DIET MSLT model, both an overall life-table and multiple disease 'state' life-tables that are mathematically linked to the main life-table. In the baseline or BAU model, the New Zealand population is projected out into the future through all-cause and disease-specific expected trends in incidence, case-fatality and mortality. The contribution of the New Zealand diet to these trends is not *explicitly* modelled in the BAU model.

The population is divided into five-year age group cohorts (from age 0 to age 105-109), modelled as four separate sex by ethnic (Māori, non-Māori) populations, and simulated in the life-table until death.

The model is a *proportional multi-state life-table model*. This basically means that:

- Everyone still alive in each cycle of the model (more specifically, the alive proportion for whichever five-year cohort is currently being modelled) is represented in the main life-table. In this main life-table, age-specific all-cause mortality and morbidity rates are applied in each cycle to the 'alive cohort', until the age of 110 years when all remaining alive people are assumed to die. As such, the sum of QALYs can be tallied.

- In parallel, proportions of the cohort can simultaneously reside in one or more parallel disease-specific life-tables or states. Or put more correctly, multiple disease states are modelled independently.[vii] Within these disease-specific life-tables, disease incidence rates, remission and case-fatality rates, and disease-specific morbidity (disability weights from the New Zealand Burden of Disease Study (BDS)[35] and GBD[36]), and disease-specific costs, are modelled.

[vii] With the exception of diabetes, which has been 'linked' to coronary heart disease and stroke states (See section 1.01.09 for details).

- The disease-specific life-tables have both a BAU and intervention model. The latter intervention model differs from the BAU model, in that incidence rates are changed (usually lowered) based on population impact fractions (PIFs; a 'merging' of changes in risk factor distributions and relative risks; see 3.01.4 later in this Technical Report). This allows a calculation of differences in disease-specific mortality and morbidity rates, and differences in disease-costs per capita.

- These differences are then summed across all parallel disease states, and added or subtracted to the all-cause mortality and morbidity rates in the main life-table and captured as cost differences between BAU and intervention, allowing estimation of QALYs gained (or lost) and health system cost change between the BAU and intervention scenarios for the population overall – the main objective of the modelling.

Figure 4: Schematic of a proportional multi-state life-table, showing the interaction between disease parameters and life-table parameters, where x is age, i is incidence, p is prevalence, m is mortality, w is disability-adjustment (or health status valuation), q is probability of dying, l is number of survivors, L is life years, Lw is health adjusted life expectancy (HALE), and where '-' denotes a parameter that specifically excludes modelled diseases, and '+' denotes a parameter for all diseases (i.e. including modelled diseases). [37]

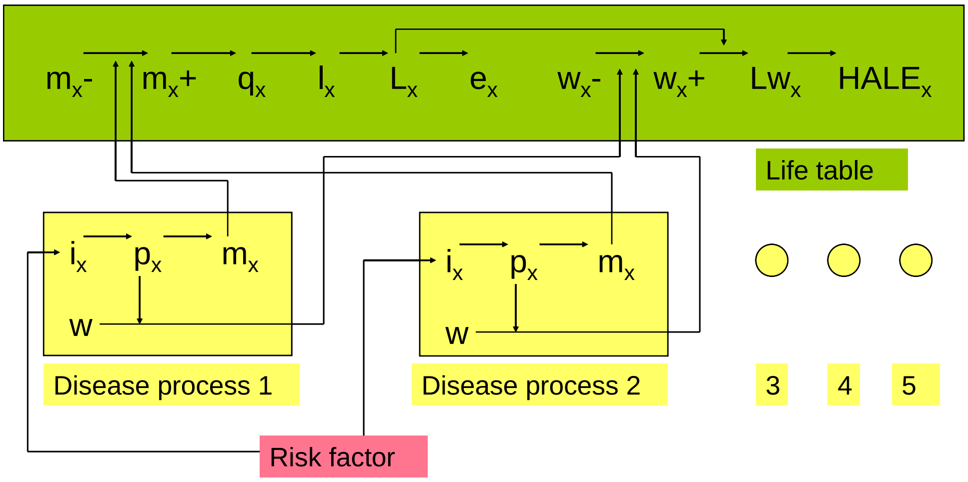

(page 30) is an alternative way of presenting a proportional multi-state life-table structure. There are numerous 'disease processes' that are modelled independently, and the total population 'experience' (in this case shown as health-adjusted life expectancy, or quality adjusted life expectancy) is a sum of these disease process contributions, and the mortality and morbidity experience due to all remaining diseases considered as one 'residual entity'. The way the intervention simulations work (not shown directly in the figure below) is to calculate changes between BAU and intervention scenarios in mortality, prevalence and disability rates for each disease process (due to changing disease incidence rates in each disease process), and then 'sum' these changes to calculate new total population (i.e. in the main life-table) mortality, prevalence and disability rates. And from here one derives a change in quality adjusted life years lived by the cohort. Other outputs like change in total mortality rate can also be outputted. Finally, health system costs can be 'attached' to the model structure in a similar way to disability or morbidity weights, allowing an estimation of change in health system costs due to changing disease epidemiology (see Section 3.02.5).

Figure 4: Schematic of a proportional multi-state life-table, showing the interaction between disease parameters and life-table parameters, where x is age, i is incidence, p is prevalence, m is mortality, w is disability-adjustment (or health status valuation), q is probability of dying, l is number of survivors, L is life years, Lw is health adjusted life expectancy (HALE), and where '-' denotes a parameter that specifically excludes modelled diseases, and '+' denotes a parameter for all diseases (i.e. including modelled diseases). [37]

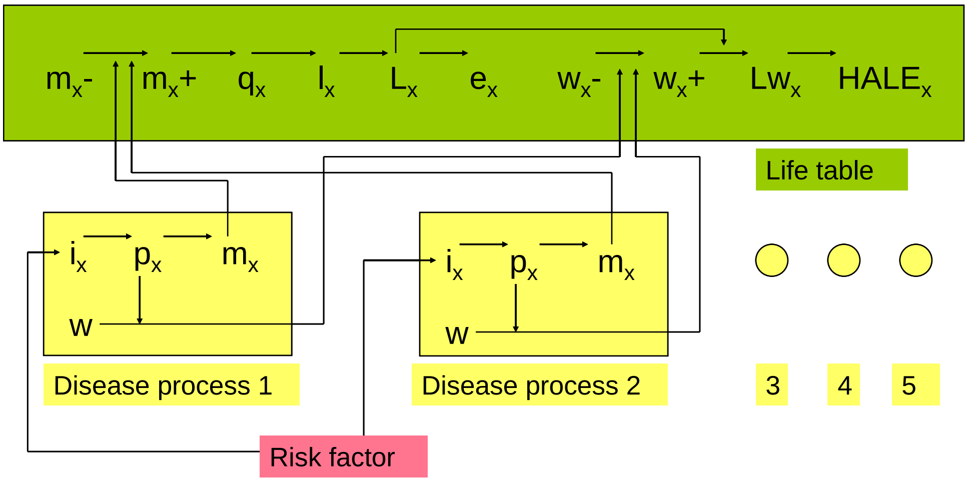

3.01.2. Diseases included in BODE[3] DIET MSLT model

Table 4 (page 31) includes all the diseases included in the BODE[3] DIET MSLT model. There are many diseases associated with diet, with varying evidence in terms of the contribution of specific dietary risk factors to their incidence. We included diseases in the model if they were included as related to specific dietary risk factors in the Global Burden of Disease Study.[38] All of the included diseases were chronic diseases (e.g. various cancers).

Table 4: Diet related diseases included in BODE[3] DIET MSLT model

NZBDS codes	ICD-10 codes	Condition
Cardiovascular disease		
E01	I20-I25	Coronary heart disease which includes "congestive heart failure"
E10	G45, G46, I63-I67	Ischaemic stroke
	I60, I61, I62	Haemorrhagic stroke
Cancers		
C50	C50	Breast cancer (females only)
C04 & C05	C18-C20	Colorectal cancer
C14	C54-55	Endometrial cancer (females only)
C07	C23-C24	Gallbladder cancer
C01	C00-C14, C30-C32	Head & neck cancer
C19	C64-C66, C68.0-C68.8	Kidney cancer
C06	C22	Liver cancer
C09	C33-34	Lung cancer
C02	C15	Oesophageal cancer
C57	C56	Ovarian Cancer (females only)
C08	C25	Pancreatic cancer
C03	C16	Stomach cancer
C21	C73	Thyroid cancer
**Other**		
N01	M15-M19	Osteoarthritis
D01	E11-E13, E14	Type 2 diabetes

NZBDS = New Zealand Burden of Disease Study.

3.01.3. Diet-related disease models

Diet has been linked to increased incidence of various cancers (e.g. colorectal), cardiovascular diseases (e.g. coronary heart disease (CHD), stroke) and osteoarthritis through dietary impacts on BMI. These diseases were modelled, within each disease process or parallel disease state as above, using a set of differential equations that describe the transition of people between four states (healthy, diseased, dead from a disease in the model, and dead from all other causes), with transition of people between the four states based on rates of background mortality, incidence, case-fatality and remission (Figure 5, page 32).

Figure 5: Each disease is modelled with four states (healthy, diseased, dead from the disease, and dead from all other causes) and transition hazards between states of incidence, remission, case-fatality and mortality from all other causes.

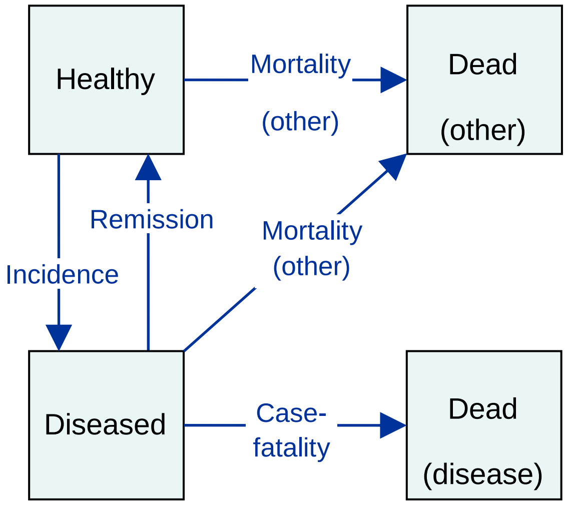

The default model structure was that diseases were modelled independently. Specifically, the sex-, age- and ethnic-specific incidence, remission, and case-fatality rates for each disease were modelled independently, e.g. the incidence rate for colorectal cancer did not vary with changes in the incidence rate (or prevalence) of kidney cancer. However, we include dependency for diabetes as a disease state, essentially treating it both as a disease state and a risk factor itself for coronary heart disease and stroke. Given this 'both a disease and risk factor' treatment of diabetes, we defer describing this model structure until after describing how risk factors are treated (i.e. Section 3.01.5).

3.01.4. How changes in risk factors change disease incidence

Health and cost impacts of simulated interventions are achieved by interventions changing risk factors (e.g. BMI) which in turn change disease incidence. This is similar to comparative risk assessment, and indeed involves 'shifts' in risk factor distributions that are merged with relative risks to determine PIFs, the percentage by which disease incidence is (usually) decreased. In this section we describe the model structure features, namely:

1. the risk factor → disease associations included in the model
2. the calculation of the PIFs
3. how decay (if any) in risk factor change is modelled over time
4. how time lags between risk factor changes and disease incidence changes are modelled.

(Actual relative risks used are given in Appendix E: Relative risks of diet to disease associations (page 96). How dietary interventions change risk factors was described in PART 1. Baseline data on risk factors was described in PART 2 Section 2.01.)

(vii) Risk factor-disease associations included in the BODE[3] DIET MSLT model

Risk factors were included if they met the following criteria:

- If they were assessed as a top risk factor (top 20) in Australasia (Australia/New Zealand) in the GBD 2010 Study.[39]
- There are interventions we plan to model that can modify this risk factor.
- There are data available:
  - o Distributional data in New Zealand (e.g. NZANS)
  - o RR data (to all key diseases; i.e. GBD sourced RRs preferable), and mutually adjusted for other risk factors in the model where possible.

Table 5 (page 33) shows the risk factor-diet associations operating in the BODE[3] DIET MSLT model. All diet-disease associations that met the above criteria were included in the model with planned modifications for future versions of the model outlined in Table 6 (page 34).

Table 5: Risk factors in Version 1 of BODE[3] DIET MSLT model, and which diet disease associations are modelled

	BMI	Fruit	Vegetables	PUFA (%TE)	SSB	Sodium
CHD	√	√	√	√		√
Ischaemic stroke	√	√	√			√
Haemorrhagic stroke	√	√	√			√
Type 2 diabetes	√				√	
Osteoarthritis	√					
Oesophagael cancer	√	√				
Colorectal cancer	√					
Breast cancer	√					
Ovarian cancer	√				√	
Stomach cancer						√
Lung cancer		√				
Head & neck cancer		√				
Pancreatic cancer	√					
Gallbladder cancer	√					
Thyroid cancer	√					
Liver cancer	√					
Kidney cancer	√					
Endometrial cancer	√					

Table 6: Risk factors for diet and Physical Activity related diseases identified by the GBD but not included in Version 1 of BODE[3] DIET MSLT model: why, and whether to be included in future Version 2

**GBD risk factors to be included in Model V2**	**Comment**
Physical inactivity and low physical activity	To be added in the next version of the model (V2).
Diet low in nuts and seeds	To be added in the next version of the model (V2).
Diet low in whole grains	To be added in the next version of the model (V2).
Diet high in processed meat	Ideally to be added in the next version of the model (V2). Firstly investigate the level of effect that is mediated through other risk factors currently in the model (e.g. Sodium). Add the risk factors into the model with appropriately modified RR.
**GBD risk factors not to be included in the model**	
Diet low in fibre	The effect of low fibre is completely mediated between 'diet low in whole grains, fruits and vegetables', risk factors either currently in the model or planned to be in the model (V2).
Diet low in seafood omega-3 fatty acids	There is no intake data for this risk factor in New Zealand.

Additionally SSB intake (ranked as the 31st top risk factor in Australasia in the GBD 2010 Study[39]) is included in the model due to the planned interventions that would impact on SSB consumption.

(viii) Calculation of PIF: Relative risk shift method

We modelled the health benefits of interventions through a reduction in incidence of each diet-related disease (Equation 4, page 35). The change in risk factor acts on the starting risk factor distribution by sex, ethnic and age groups. For each risk factor there are up to 10 categories of risk (e.g. For BMI: <20, 20-25, 25-30, 35-40, 40-45 and 45+; six categories). The proportion of the population for each sex, ethnic and age group that sits in each of those categories is obtained from the NZANS. This proportion, the category midpoint and the relative risk associated with that risk factor are mathematically combined with the effect size to calculate the PIF for each risk factor disease combination – not by shifting proportions of the cohort by category, but rather by shifting the RR to what it would be for the new midpoint of the same starting category under the intervention [40] (more below). Note that all calculations were done by age, sex and ethnicity, although we omit these subscripts from the following equations for clarity.

$$\mathrm{I}_x' = \mathrm{I}_x \times (1 - \mathrm{PIF}_x) \tag{2}$$

where:

I_x = the current incidence of disease *x* in the population;

I_x' = the new incidence of disease *x* after an intervention is implemented; and

PIF_x = is the population impact fraction for disease *x*.

A PIF [41] is derived for each risk factor disease combination. For example, for CHD there were PIFs for the association between each of fruit, vegetables, BMI, sodium, percentage of total energy from polyunsaturated fatty acids and CHD.

The PIF is calculated using the Relative Risk shift method.[40] This method changes the relative risk of the categories and keeps the proportion in each category constant. For example, if categories are formed for every 5-point increase on the continuous scale (e.g. BMI), and the RR per 5-point increase was 1.5, and the intervention lowers everyone's (and therefore the category midpoints) risk factor by 1 unit, then each categories RR is lowered by 0.1 if RR on linear scale or multiplied by exp(ln(1.5)/5)=1.0845 if on log-linear scale. For a risk factor with n categories, the equation for the PIF is:

$$\mathrm{PIF} = \sum_{c=1}^{n} P_c RR_c - \sum_{c=1}^{n} \mathrm{P_c RR_c^*} \Big/ \sum_{c=1}^{n} P_c RR_c \tag{3}$$

where:

P_c = the proportion of the population in category c;

RR_c = the RR for category c;

RR_c^* = the RR for category c after the intervention; i.e. the 'shifted' RR;

'Per unit' RR from the GBD were used for the RR for category c after the intervention.

The PIFs, most simply, combine the relevant effect size with the relative risk for the risk factor disease combination. 1-PIF is used to change incidence (see equation 2, page 34). For diseases with multiple risk factors, (1-PIF) are multiplied together to give an overall (1-PIF) (Equation 4). For the CHD example there are (1-PIF)s for fruit, vegetables, BMI, sodium, percentage of total energy from polyunsaturated fatty acids and physical activity which are multiplied together to give the overall effect of the intervention on CHD.

$$1 - \mathrm{PIF}_{\mathrm{Final}} = \prod_{i=1}^{n} (1 - \mathrm{PIF}_{\mathrm{i}}) \qquad (4)$$

where:

n= the number of risk factors;

Scaling of risk factor distribution and category midpoints

For the majority of the risk factors the risk factor distributions are taken straight from the NZANS as described above, however additional scaling is done for Sodium and SSB intakes. Sodium intake data is scaled to sodium excretion data as described in Section 1.01.2. SSB intake data are scaled to approximate usual intake as described below.

SSB intake to approximate usual intake

The majority of risk factors in the DIET model are foods or nutrients that will be consumed on a daily basis. SSBs on the other hand are a periodically consumed food group. GBD relative risk estimates are based on SSB consumption as recorded by food frequency questionnaires, and therefore represent estimates for usual intake of SSBs. Data from a single 24-hr recall is unlikely to accurately represent usual consumption of SSBs. Firstly, a single 24-hr recall is likely to underestimate the proporiton of the population that consume some SSBs. Secondly, a single 24-hr recall is likely to overestimate the amount of SSBs consumed by individuals who do have SSB consumption recorded on the day of the survey. For

these reasons, we rescaled SSB intakes from 24-hr recall data in NZANS to obtain a better estimate of usual population SSB intake.

We combined data from the overall NZANS sample with the subsample of the survey for whom two 24-hr recalls were recorded. This allowed us to calculate the probability of being a SSB consumer, and (for consumers) the probability of consuming SSBs on any given day. At the individual level, we then predicted whether an individual was a true zero consumer and if not, we predicted a weekly frequency of SSB consumption. SSB intakes for (predicted) consumers were then scaled based on (predicted) consumption frequency to avoid overestimating SSB consumption in consumers. For example, an individual with 500ml SSB intake recorded in the single 24-hr recall with a predicted frequency of consumption of two days per week was assigned an estimated usual SSB intake of 143ml (1000ml estimated weekly total divided by seven). Estimates of usual intake for (predicted) consumers without consumption recorded in the single 24 hr recall were based on average recorded intake values for their age, sex, and ethnic group.

We simulated individual intakes 10,000 times and averaged across the runs to obtain estimates of population distributions of SSB intake. Each simulation randomly assigned different individuals with different frequency of consumption values, and also accounted for the survey standard error around initial estimates of the probability of ever-consumption and consumption on any given day.

Theoretical Minimum Risk Exposure Level (TMREL)

In the Comparative Risk Assessment (CRA) approach, attributable burden is calculated in reference to a counterfactual risk exposure. In this modelling the counterfactual used is the Theoretical Minimum Risk Exposure Level (TMREL). The TMREL is a theoretically possible level of intake that minimizes overall risk. This allows us to quantify how much of the disease burden could be lowered by shifting the risk factor distribution to a ‘theoretically possible’ level associated with the greatest improvement in population health [1]. As the evidence for the TMREL is uncertain for the risk factors modelled, a range or uncertainty interval about the TMREL is used rather than just a central estimate.

Table 7: Range or uncertainty intervals for risk factor TMRELs

Risk factor	Range or uncertainty interval about the TMREL
BMI	21-23; BMI *above* some value in this range deleterious
Diet high in sugar-sweetened beverages	Consumption of SSBs between 0g and 64.3g per day; SSBs *above* some value in this range deleterious
Diet high in sodium	Consumption of sodium between 1g and 5g per day; sodium *above* some value in this range deleterious
Diet low in fruits	Consumption of fruit between 200g and 400g per day; fruit *beneath* some value in this range deleterious
Diet low in vegetables	Consumption of vegetables between 350g and 450g per day; vegetables *beneath* some value in this range deleterious
Diet low in polyunsaturated fatty acids	Consumption of polyunsaturated fatty acids between 10% and 15% of total daily energy; %PUFA *beneath* some value in this range deleterious.

For risk factors where lower BMI or intake decreases disease incidence (BMI, SSBs and sodium), in those categories whose midpoints are lower than the TMREL then there is no effect. For risk factors where higher intake decreases disease incidence (fruits, vegetables and polyunsaturated fatty acids) the method works in reverse; those categories whose midpoints are higher than the TMREL there is no effect, i.e. people are already receiving maximum benefit from their high consumption.

(ix) Modelling decay or attenuation of effect

Many interventions, such as dietary counselling, have attenuating effects. For example, a particular dietary counselling regime may change population average BMI by 0.1 unit initially, but over the next 'x' years the population tends to regain weight back to their BAU levels. The length and shape (e.g. linear or exponential to return back to BAU) of this decay is informed by evidence relevant to the specific interventions modelled e.g. Dasinger et al. (2007)[42], and specified in the model.

(iv) Time lags

Changing diet does not usually rapidly change disease incidence; it takes time for disease incidence to change to a 'new equilibrium'. Evidence on time lags, and the shape of change in disease incidence, following dietary change, is very limited. Some simulation studies circumvent this by assuming the change in disease incidence is immediate. However, this will (grossly) over-estimate the effect of dietary intervention on cancer incidence (where time lags are likely to be decades, and moderately overestimated changes in cardiovascular disease (where time lags might be months to years). This issue of time lags is compounded by discounting (i.e., little net benefit might be seen with a cancer preventing diet where a high discount rate is used in the model).

The approach we used was to look back to the average (1-PIF) reflecting the average change in risk factor in a past window of exposure. For example, the relevant time of exposure to increased fruit consumption on current CHD incidence may be the previous 5 years. Thus, we use the average (1-PIF) in the last five years. For cancers, it might take at least 10 years for any (notable) change in disease

incidence to occur, and any benefit on disease incidence might last up to 30 years. Therefore, we would use the average (1-PIF) for 10 to 30 years ago. There is considerable uncertainty in these time lags. Therefore, we:

- Specify the minimum and maximum time lags (e.g. 10 and 30 years for cancers)
- And additionally make these parameters uncertain themselves (e.g. 20% SD normal distribution about minimum and maximum).
- And calculate the average (1-PIF) within this look-back time lag range.

We will include these parameters in actual publications, but in principle the following parameters (by disease) are the 'default'.

Table 8: Default parameters for time lags in Diet MSLT

Disease	Minimum lag	Maximum lag
**"Short" time lag**		
CHD	1	5
Stroke	1	5
Type 2 Diabetes	1	5
Osteoarthritis	1	5
**"Long" time lag**		
All cancers	10	30

3.01.5. Diabetes: both a disease and a risk factor

The MSLT has key independence assumptions, including:

1. *Risk factor distribution:* the distributions of each risk factor can be treated as though independent of other risk factors.
2. *Disease incidence rates:* the incidence rate for a given disease (e.g. CHD) is independent of other diseases (e.g. the presence of diabetes).
3. *Disease case-fatality and remission rates*: the rates for a given disease (e.g. CHD) are independent of those for other diseases (e.g. diabetes).

The second assumption is the focus here, for diabetes. Diabetes is associated with increased rates of coronary heart disease and stroke (and some cancers), be it by shared common causes (i.e. confounding) or cause and effect (the concern here). Whether to address such 'dependency' depends on what one is doing with the model, through what risk factors. For the BODE[3] DIET MSLT model, interventions that change BMI and thence disease incidence are important. Figure 6 (page 39) gives the standard structure. BMI is independently associated with each of CHD and Diabetes Mellitus (DM), and change (Δ) in the BMI distribution combined with the relative risk for the BMI→CHD and BMI→DM association to give a PIF results in a change in both disease incidence rates. The change in mortality, morbidity and cost rates that result are then 'added' to the overall mortality, morbidity and cost rates in the main life-table.

Figure 6: Standard structure in MSLT for BMI as risk factors and CHD and DM states

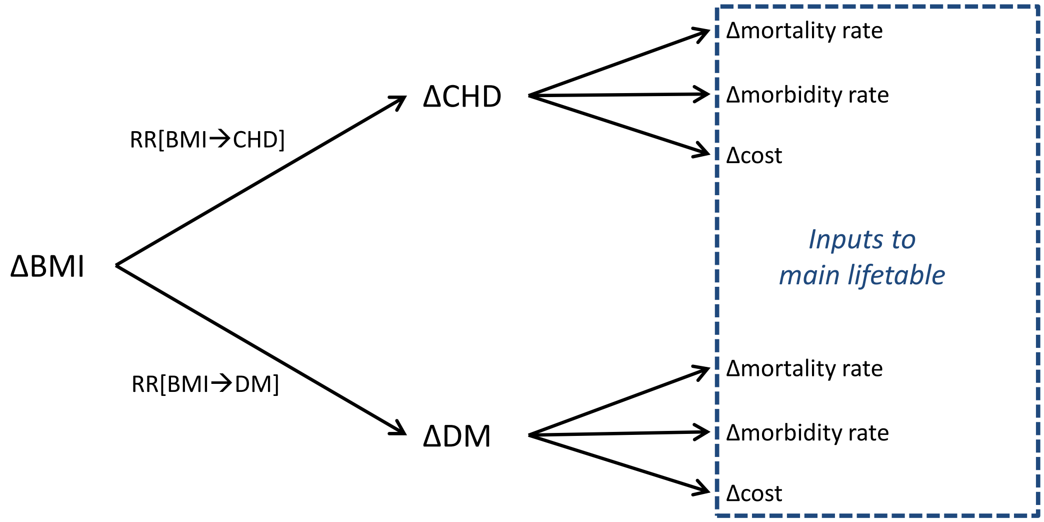

A modelled intervention that lowers BMI may result in an overestimated QALYs if the reduction in diabetes and coronary heart disease 'double-count' the gains when considered independently. But if only the 'pure diabetes' mortality rate (e.g. based on the deaths coded as DM) is estimated in the DM state, and the higher than average population mortality rate otherwise (e.g. due to people with DM having higher CHD and stroke mortality) is not allowed for, the prevalence of DM will drift too high over time as the total mortality rate modelled for diabetics is not high enough. This over-estimated morbidity rate, in turn, may lead to an overestimate of morbidity gains due to a BMI lowering intervention. (And likewise an overestimate of costs savings as costs are a function of prevalence.)

One solution to this dependency problem is a microsimulation model, where each individual's other disease status is 'known'. But for the BODE^3 DIET MSLT model, the partial solution we use is to restructure and re-parameterise the model.

Figure 7 below gives that structure. The changes are:

- To 'link' the DM state to the CHD state (and stroke state; not shown), such that:
  - DM becomes a risk factor for CHD, linked through a RR that is adjusted for BMI (which is now a confounder of the DM→CHD association). Specifically, a change in the DM prevalence changes CHD incidence through a PIF link.
  - The RR for the BMI → CHD association is now the 'direct effect' [43], i.e. that not through DM.
  - The outputs from the CHD state that input to the MSLT remain unchanged in structure.
- The mortality rate output from the DM to the main life-table in the MSLT is:
  - 'just' the mortality rate due to deaths coded as DM in the default model. This use of a case fatality rate due to DM-coded deaths only is likely an underestimate of the death due to DM. However, the CHD and stroke excess deaths are explicitly modelled through

the PIF link from the DM prevalence to CHD and stroke incidence. And the excess rate of other deaths due to DM will (partly at least) be implicitly captured through the changes in (say) BMI to cancer that includes some unquantified pathway through diabetes.

  - given the uncertainty above in the default model, as a sensitivity analysis we model excess mortality among people with DM from having diabetes, excluding CHD and stroke mortality as that is quantified in, and outputted from, the CHD (and stroke) states instead of the DM-only case fatality rate above. This will probably overestimate the mortality due to DM, but does give an upper limit.

- But to ‘allow’ for the higher mortality rate among diabetics, a ‘total excess’ mortality rate (mort[all-cause|DM] – mort[all-cause], where the former is the all-cause mortality rate among diabetics, and the latter is the all-cause mortality rate in the general population without DM) is applied within the DM state as an absorbing state. This mortality is only used to ‘kill people off’ in the model to allow for dependent mortality risk; without this higher mortality rate taking people out of the alive DM population, the prevalence would drift too high (impacting on costs and morbidity).

Figure 7: Altered structure in the BODE[3] DIET MSLT to allow for dependency of CHD (and stroke – not shown) on DM

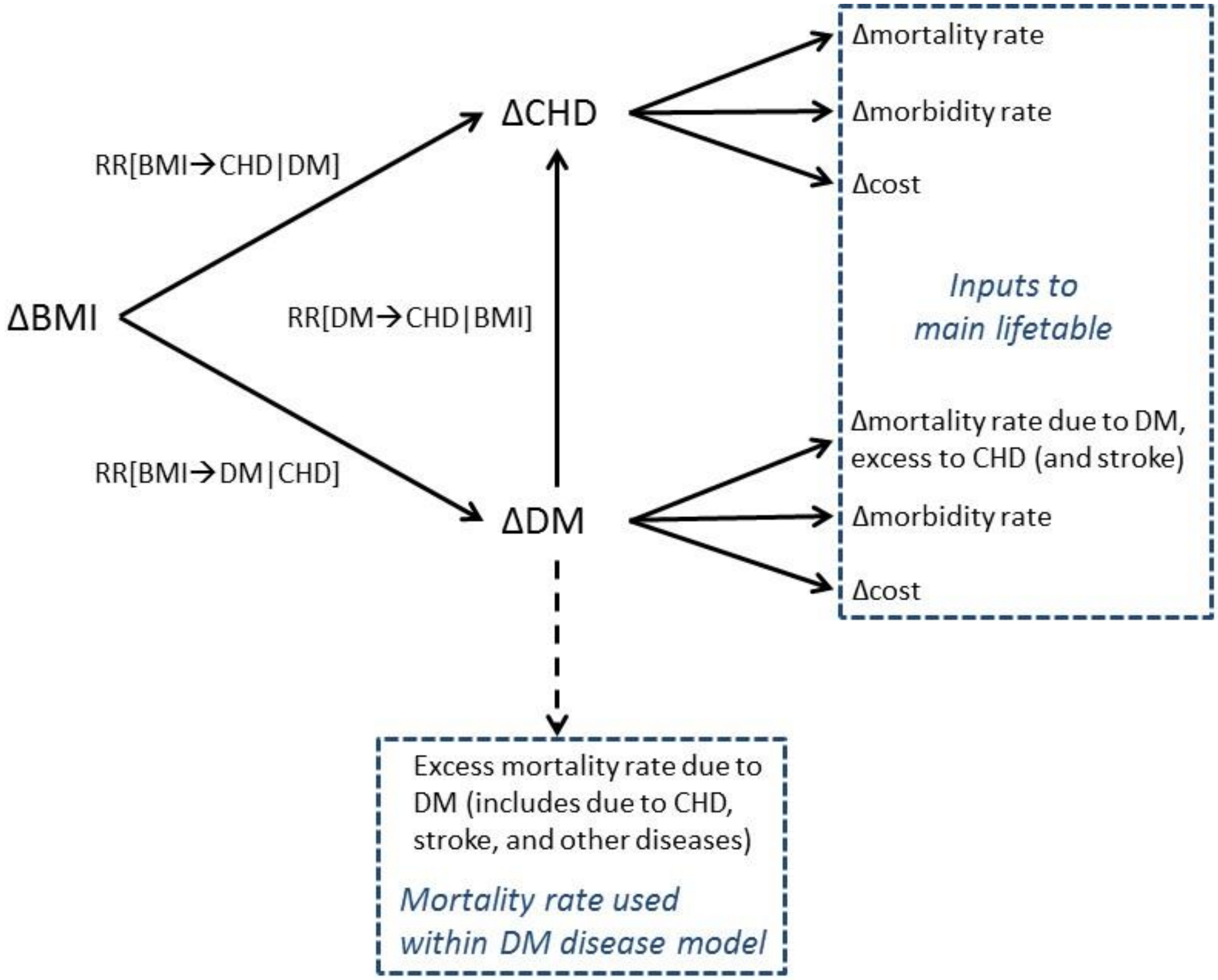

Figure 8 (page 42) gives an alternate depiction of the mortality 'stack'. The total of all components is the total mortality rate among diabetics. 'C', 'S' and 'O' are, respectively, the excess CHD, excess stroke, and excess non-CHD non-stroke mortality among people with DM compared to the general population, and 'O' is partitioned again into DM-coded deaths and non-DM coded deaths.

Figure 8: Partitioning of total mortality rate among people with DM into components relevant to BODE[3] DIET MSLT structure

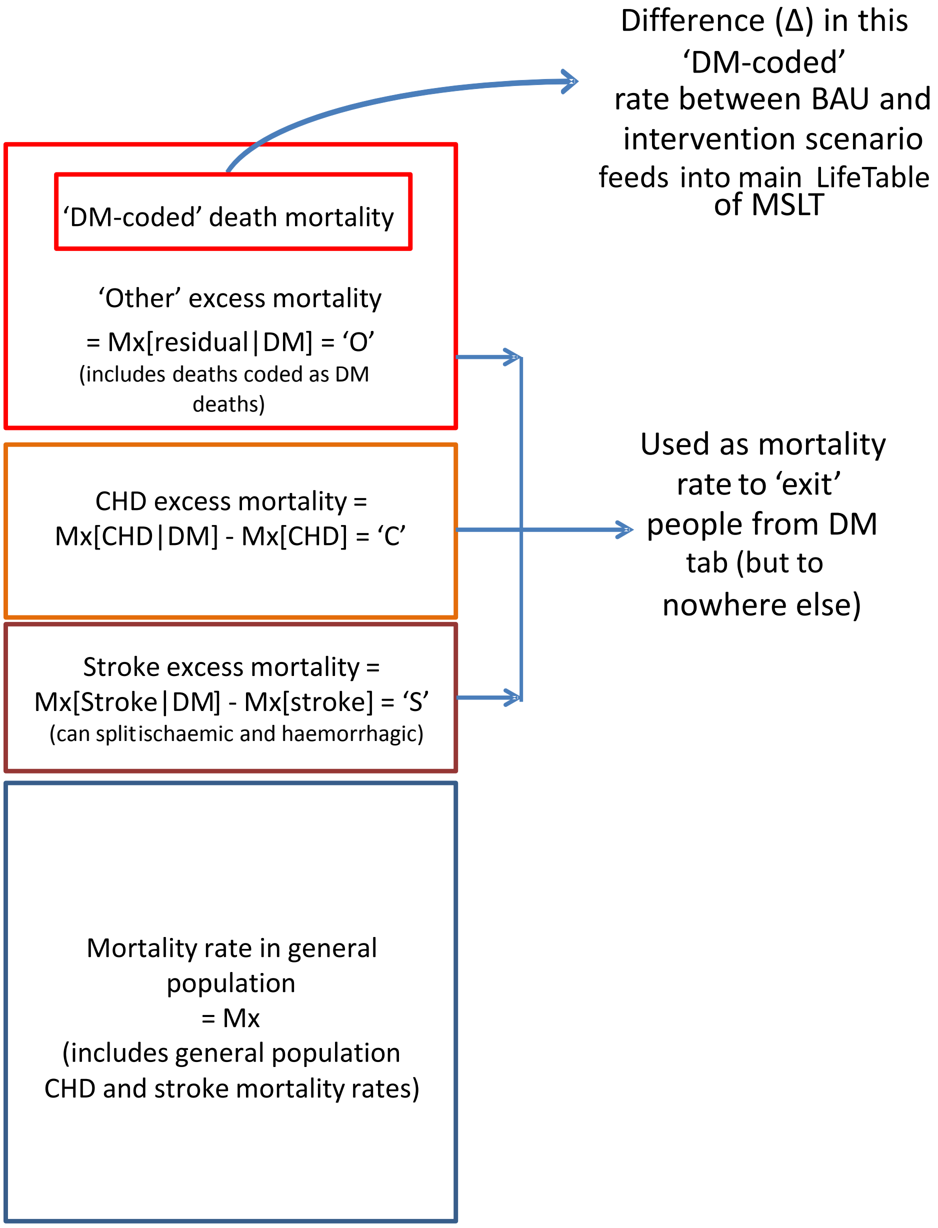

The above structure (Figure 7 and Figure 8, pages 41 and 42) and parametrization is an improvement for a disease like DM. However, it is not perfect.

The parameterisation of this modification to the MSLT requires recalculation of baseline or BAU parameters, and intervention parameters. Rather than present it either here (before such parameterisation has been described for the main model), we give a full description of how the above model alteration was specified in Appendix D: Parameterisation of 'DM as both a risk factor and disease' (page 89).

Section 3.02. Baseline specification and parameters

3.02.1. Background population inputs

The following population parameters were included: 1) population size; 2) total prevalence years lived with disability (pYLDs); and 3) total mortality rates, all by 5-year age groups for each sex and ethnicity. Population counts were compiled using Statistics New Zealand 2011 estimates. Total pYLDs were calculated using the total (corrected for multiple morbidity) YLDs for all diseases in the NZBDS divided by the total population in New Zealand for each age, sex and ethnicity group. Population mortality rates were calculated using data from the Statistics New Zealand life tables for 2010-2012. Annual reductions in background population mortality were assumed to be 1.75% for non-Māori and 2.25% for Māori out to 2026 [44], then held constant.

3.02.2. Data sources, processing, DISMOD, and inputs to BODE^3 DIET MSLT model

The basic steps for generating disease inputs for the BODE^3 DIET MSLT model were: 1) data compilation; 2) preliminary processing of the data; and 3) DISMOD II estimation of epidemiologic parameters [45].

Step 1: Data for these diseases were compiled from various sources (see Table 9).

Step 2: Some parameters were further processed to give 'best' (pre-DISMOD) estimates for 2011. For example, data on prevalence for less common diseases were compiled and then regression-smoothed prior to inputting into DISMOD II. Readers can refer to Appendix C: DISMOD II example for lung cancer (page 86) for a step by step description of data compilation and processing in DISMOD II for one example, lung cancer. (Similar documentation for all other diseases is available from the authors on request.) All parameters were generated by 5-year age groups by sex and ethnicity (Māori/Non-Māori), except breast, ovarian and endometrial cancers which were only compiled for women.

Step 3: These parameters were then inputted to DISMOD II, separately by sex and ethnicity, to generate a mathematically and 'epidemiologically consistent' set of parameters. For example, if the prevalence estimate was too low given what is known about incidence and case-fatality from the disease (and background 'competing' mortality), DISMOD II outputs values that are epidemiologically / mathematically consistent, allowing the user to 'weight' the inputs. For cancers, full weighting (setting at "100%") was given to incidence, as it was the most reliable parameter (due to New Zealand Cancer Registry data). Typically, mortality was also given full weighting and prevalence was given a 50% weighting (for disease-specific weighting information, README files for the disease of interest available upon request from the authors, and for lung cancer (only) in the Appendix C: DISMOD II example for lung cancer page 86). For DM, stroke and CHD, we additionally included time trends in incidence and case fatality inputs to DISMOD II, given the strong time trends in these diseases.

Table 9: Diet-related disease data sources and processing notes for those disease variables subsequently estimated in DISMOD II

Disease	Incidence	Prevalence	Disease-specific mortality	Case-fatality rate	Remission
Breast cancer	BODE[3] estimates^	NZBDS, 5-year look back	NZBDS	Generated using equations†	Generated using equations†
Colorectal cancer	BODE[3] estimates^	NZBDS, 5-year look back	NZBDS	Generated using equations†	Generated using equations†
Endometrial cancer	BODE[3] estimates^	NZBDS, 5-year look back	NZBDS	Generated using equations†	Generated using equations†
Gallbladder cancer	BODE[3] estimates^	NZBDS, 5-year look back	NZBDS	Generated using equations†	Generated using equations†
Head & neck cancer	BODE[3] estimates^	NZBDS, 5-year look back, regression smoothed	NZBDS, regression smoothed	Generated using equations†	Generated using equations†
Kidney cancer	BODE[3] estimates^	NZBDS, 5-year look back, regression smoothed	NZBDS, regression smoothed	Generated using equations†	Generated using equations†
Liver cancer	BODE[3] estimates^	NZBDS, 5-year look back	NZBDS	Generated using equations†	Generated using equations†
Lung cancer	BODE[3] estimates^	NZBDS, 5-year look back	NZBDS	Generated using equations†	Generated using equations†
Oesophageal cancer	BODE[3] estimates^	NZBDS, 5-year look back, regression smoothed	NZBDS, regression smoothed	Generated using equations†	Generated using equations†
Ovarian cancer	BODE[3] estimates^	NZBDS, 5-year look back	NZBDS	Generated using equations†	Generated using equations†
Pancreatic cancer	BODE[3] estimates^	NZBDS, 5-year look back, regression smoothed	NZBDS, regression smoothed	Generated using equations†	Generated using equations†
Stomach cancer	BODE[3] estimates^	NZBDS, 5-year look back	NZBDS	Generated using equations†	Generated using equations†
Thyroid cancer	BODE[3] estimates^	NZBDS, 5-year look back, regression smoothed	NZBDS, regression smoothed	Generated using equations†	Generated using equations†
Non-cancer outcomes					
CHD	HealthTracker, regression smoothed for Māori only	NZBDS, 10-year look back	MOH	No direct input, output determined by DISMOD	Set to 0
Stroke	HealthTracker	HealthTracker	MOH	No direct input, output determined by DISMOD	Set to 0
Type 2 diabetes	HealthTracker	HealthTracker	HealthTracker	HealthTracker	Set to 0
Osteoarthritis	HealthTracker	HealthTracker	Set to 0	Set to 0	Set to 0

^Source:[46]

† Using simple assumptions about the mathematical relationship of prevalence and incidence to generate an average duration, then a total rate of 'exit' (i.e. remission rate + case-fatality rate (CFR) + background mortality rates), and then estimating the case-fatality rate given the five year relative survival ≈[Remission]/[Remission + CFR].

The DISMOD output rates (in one year age groups) for incidence, prevalence, case-fatality and remission were then used to populate the BODE[3] DIET MSLT model for all diseases – except CHD, stroke, type 2 diabetes and osteoarthritis. For CHD, stroke, type 2 diabetes and osteoarthritis, only incidence, prevalence, and case-fatality were used (i.e. remission was assumed to be zero as these are usually life-long conditions).

Disability rates (DRs) were calculated by dividing the NZBDS's disease-specific pYLDs (adjusted for other co-morbidities, for the year 2006, projected to 2011) by the DISMOD II estimated prevalent cases for all diseases. To estimate the pYLDs in 2011 we applied the following equation:

$$YLD_{2011} = YLD_{2006} \times (1 + APC_{inc})^5 \times \frac{1}{\left(1 + APC_{cfr}\right)^5 \times (1 + APC_{rem})^5} \times \frac{Popn_{2011}}{Popn_{2006}} \tag{5}$$

Where:

YLD[2006] is the "corrected for comorbidities YLD" in 2006 from NZBDS
APC[inc] = annual percentage change in incidence rate for each disease
APC[cfr] = annual percentage change in CFR for each disease
APC[rem] = annual percentage change in remission rate for cancers only
Popn[2006] = the population count/size for the given sex by age by ethnic group in 2006
Popn[2011] = the population count/size for the given sex by age by ethnic group in 2011

For specific details on final parameters for each disease, see Table 10 below.

Generating DRs by dividing pYLDs by prevalent cases for each 5-year age group, for each disease, for each sex by ethnicity, was often too unstable due to sparse data. We therefore aggregated age groupings to ensure the sum of prevalent cases exceeded 10 (e.g. 0-44 year olds were always combined; for common diseases such as CHD and stroke age groupings were: 0-44, 45-54, 55-64, 65-74, and 85+ years; for rare diseases such as pancreatic cancer in Māori males all age groups were combined).

Table 10: Final disease parameters and sources used in the BODE[3] DIET MSLT model

Disease	Incidence	Prevalence	Case-fatality rate	Mortality rate	Remission	Disability rate
CHD	DISMOD II	DISMOD II	DISMOD II			DISMOD II & NZBDS
Stroke	DISMOD II	DISMOD II	DISMOD II			DISMOD II & NZBDS
Type 2 diabetes	DISMOD II	DISMOD II	DISMOD II			DISMOD II & NZBDS
Osteoarthritis	DISMOD II	DISMOD II	DISMOD II			GBD DW
Breast cancer	DISMOD II	DISMOD II	DISMOD II		DISMOD II	DISMOD II & NZBDS
Colorectal cancer	DISMOD II	DISMOD II	DISMOD II		DISMOD II	DISMOD II & NZBDS
Endometrial cancer	DISMOD II	DISMOD II	DISMOD II		DISMOD II	DISMOD II & NZBDS
Gallbladder cancer	DISMOD II	DISMOD II	DISMOD II		DISMOD II	DISMOD II & NZBDS
Head & neck cancer	DISMOD II	DISMOD II	DISMOD II		DISMOD II	DISMOD II & NZBDS
Kidney cancer	DISMOD II	DISMOD II	DISMOD II		DISMOD II	DISMOD II & NZBDS
Liver cancer	DISMOD II	DISMOD II	DISMOD II		DISMOD II	DISMOD II & NZBDS
Lung cancer	DISMOD II	DISMOD II	DISMOD II		DISMOD II	DISMOD II & NZBDS
Oesophageal cancer	DISMOD II	DISMOD II	DISMOD II		DISMOD II	DISMOD II & NZBDS
Ovarian cancer	DISMOD II	DISMOD II	DISMOD II		DISMOD II	DISMOD II & NZBDS
Pancreatic cancer	DISMOD II	DISMOD II	DISMOD II		DISMOD II	DISMOD II & NZBDS
Stomach cancer	DISMOD II	DISMOD II	DISMOD II		DISMOD II	DISMOD II & NZBDS
Thyroid cancer	DISMOD II	DISMOD II	DISMOD II		DISMOD II	DISMOD II & NZBDS

3.02.3. Final processing of incidence and prevalence estimates

In an effort to more accurately reflect the disease epidemiology in the New Zealand population, some diseases incidence and prevalence rates were forced to be zero at young ages as a final step in processing. Specifically, the incidence and prevalence rates for all cancers were set to 0 for those 20 years and younger, for CHD they were set to 0 for 18 year olds and younger and for stroke they were set to 0 for 24 year olds and younger.

3.02.4. Future disease trends (incidence, remission and case-fatality)

The above parameterisation was for 2011 only. Some key parameters are known to have increasing or decreasing trends in recent decades – and are likely to have such trends in the near-future. Thus, we also specified future disease incidence and case-fatality as percentage annual change from 2011 to 2026. For CHD and stroke, we relied on NZBDS projections for annual changes in incidence and mortality (see Table 8 in a Report [47]). Specifically, we incorporated an annual incidence change of -2% and an annual case-fatality trend of -2% for CHD and stroke.

For cancer trends, we relied on our previous modelling of future cancer incidence.[46] We generated average incidences for cancer types by sex and ethnicity for age groups in the 25-85+ year range for the years 2006 to 2026. Then we calculated trends for individual age-groups as "ln(rate_2026/rate_2006)/20" and took a weighted average (weighted on incidence 2006) across age groups. However, since the NZBDS did not develop projections for changes in cancer case-fatalities, we calculated the average case-fatality rate and remission rate for those aged 45-84 years in 2011 (DISMOD outputs/multistate life-table disease inputs) by sex and ethnicity for each cancer. We then used the coefficient for year since diagnosis from the Excess Mortality Rate models (Table 30 in;[46] http://www.otago.ac.nz/wellington/otago032865.pdf) to calculate annual percentage change in case-fatality and remission.

Uncertainty around the incidence, case-fatality and remission disease trends were included in the model for all diseases of 1 percentage point SD about the annual percentage change. This uncertainty draw is independent for each epidemiological parameter (i.e. incidence, case-fatality

and remission) by disease, but correlated r=1.0 across each of the four sex by ethnic groupings and all diseases.

3.02.5. Disease health system cost inputs

Just as proportions of the cohort 'alive' in the overall and disease process are rewarded with additional QALYs for each annual cycle they live, so too can health system costs be 'rewarded'. In the BODE[3] DIET MSLT model, we have five types of health system cost:

- Main life-table:
  A. Annual cost to the New Zealand health system for being alive for a given sex and age, and *not* in the last six months of life and *not* concurrently alive with one of the modelled diseases (i.e. diet-related disease). All members of the cohort are assigned this cost; it is the base cost.
  B. Excess cost to A for being in the last six months of life if dying of a disease *other than* one of the modelled diseases (i.e. dying of a non-diet-related disease).
- Disease process life-tables:
  C. Excess cost to A for being in first year of diagnosis of a diet-related disease.
  D. Excess cost to A for being alive with a diet-related disease, and neither in the first year of diagnosis nor in the last six months of life if dying of that disease.
  E. Excess cost to A for being in the last six months of life if dying of a diet-related disease.

Cost offsets, due to reduced rates of diet-related disease, are calculated using these five types of health system cost, and changing the 'flow' through the multi-state life-table by altering disease incidence.

We sourced these five costs from the New Zealand HealthTracker database for all diseases except diabetes, which was sourced through the Virtual Diabetes Register (VDR). The specific details and equations of how these costs, developed within BODE[3], are calculated are detailed in the following online Report: "Kvizhinadze G, Nghiem N, Atkinson J, Blakely T. Cost Off-Sets Used in BODE[3] Multistate Lifetable Models Burden of Disease Epidemiology, Equity and Cost-Effectiveness Programme (BODE[3]). Technical Report: Number 15. Wellington: University of Otago, Wellington, 2016" (at: http://www.otago.ac.nz/wellington/otago619391.pdf).

All costs are in 2011 New Zealand dollars. At the time of writing this Technical Report (2017), HealthTracker costs were available for the 2006 to 2012 period. For examples of cost parameters by disease, see Appendix D: Parameterisation of 'DM as both a risk factor and disease' (page 89). All costs are being made available on the BODE[3] website (www.otago.ac.nz/bode3), and are subject to ongoing improvements. Of note, health system costs will be updated in the future (as more years of data are accrued, and with 'improvements' to scale costs to more accurately reflect VOTE: Health), productivity costs (human capital approach) will be added in future models.

Section 3.03. Calibration

Calibration has been described as ensuring that "inputs and outputs are consistent with available data".[48,49] To a large extent, the BODE[3] DIET MSLT model is self-calibrating on inputs; the model uses

total New Zealand population data for 2011, with some modification (usually slight) with DISMOD II to ensure epidemiological coherence.

As an additional calibration check, we compared the following rates for CHD, stroke and diabetes, in Figure 9 to Figure 14 (pages 49 to 54):

- MSLT model input incidence, case fatality and prevalence – which are actually outputs from DISMOD II.
- DISMOD mortality rates. They are neither inputs nor outputs for the MSLT, but are one of the rates used in DISMOD to develop the coherent set of epidemiological parameters – most notably the case fatality input rate.
- MSLT model output prevalence and mortality rates. These differ from the DISMOD mortality and prevalence rates, as they are determined dynamically within the model as the cohorts (aged 2, 42 or 72 in 2011) age within the model.

The model check is that we expect the output prevalence and mortality rates to differ somewhat – but not too much – from the input prevalence and mortality rates given what we know about epidemiological trends and transitions. In brief, they appear to, and thus provide a form of calibration 'check' on the model.

In more detail, consider first the CHD rates in Figure 9 (page 49) and Figure 10 (page 50). The DISMOD and output mortality rates are virtually indistinguishable as the cohorts age. The output prevalence, however, is a bit lower. But this is coherent. The inputs are the rates in 2011. As CHD incidence is falling so rapidly, the prevalence as recorded in 2011 is higher than what it would have been if incidence had not been falling in the past. Put another way, for these graphs where 2011 rates are used as inputs with no future time trends, the prevalence rate is at 'equilibrium' for these inputs, whereas the prevalence as recorded in 2011 is not at equilibrium. Thus, we conclude the CHD rates are plausible and coherent.

Stroke rates are shown in Figure 11 (page 51) and Figure 12 (page 52). There is closer agreement than with CHD.

Diabetes rates are shown in Figure 13 (page 53) and Figure 14 (page 54). Here the pattern is the reverse of that for CHD, which is plausible and coherent as diabetes incidence rates have been increasing (and case fatality rates decreasing) that the observed prevalence rates by age in 2011 are less than the 'equilibrium' prevalence rates over time into the future outputted by the model.

As at younger ages it is a bit difficult to see differences in rates on an absolute scale, Appendix G: Model rates vs. DISMOD rates, log graphs from Section 3.04.5 (page 119) gives replicates of these calibration graphs with rates on a log scale. It is only with diabetes that a difference in prevalence rates between input and output series remains evident.

Figure 9: CHD rates, by sex and ethnic group cohorts 2 years of age in 2011. DISMOD prevalence and mortality rates, and case fatality rates, are those inputted to the BODE[3] DIET MSLT; other rates are those outputted over the remainder of the cohort's life

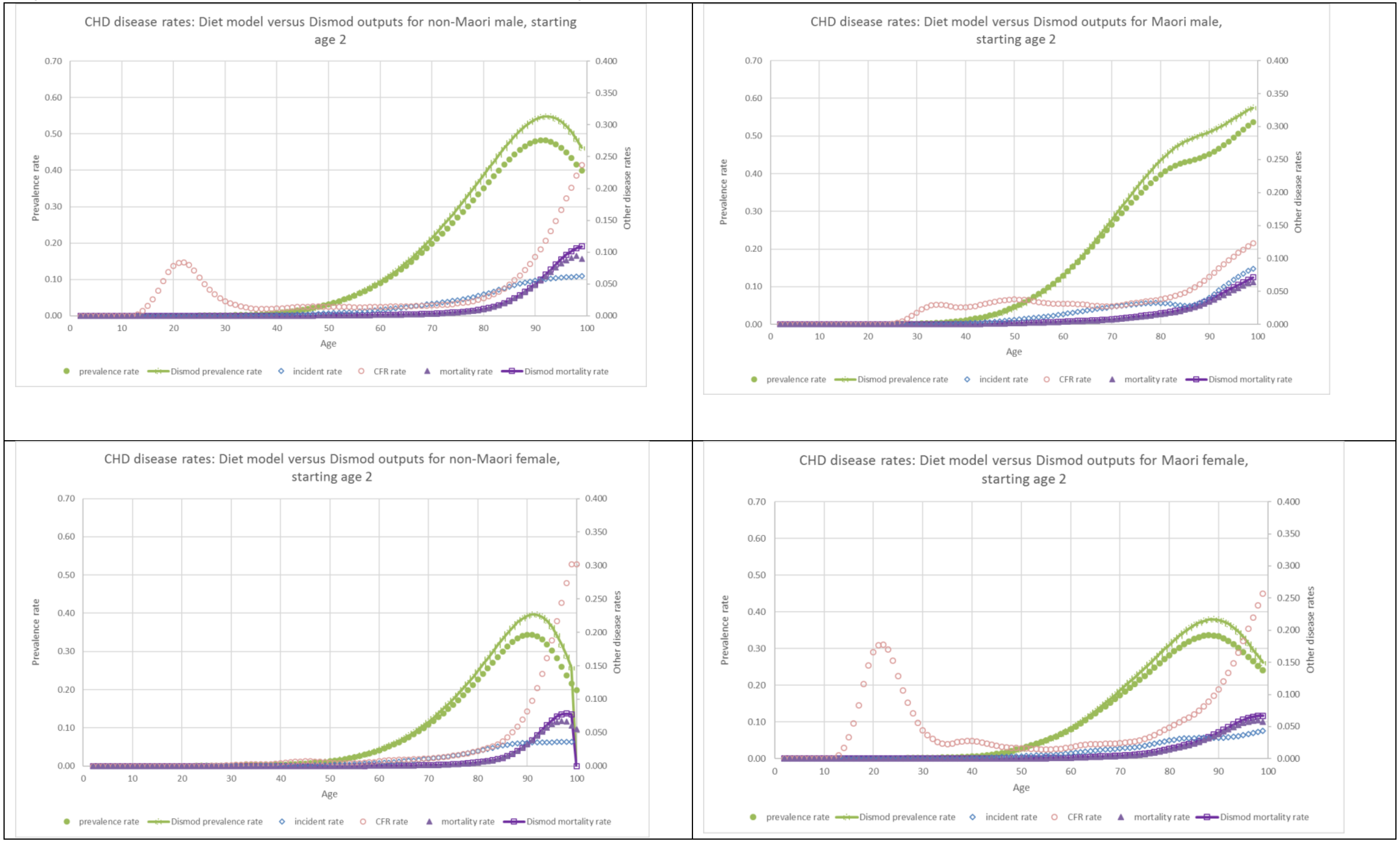

Figure 10: CHD rates, for non-Māori males aged 42 and 72 years of age in 2011. DISMOD prevalence and mortality rates, and case fatality rates, are those inputted to the BODE[3] DIET MSLT; other rates are those outputted over the remainder of the cohort's life

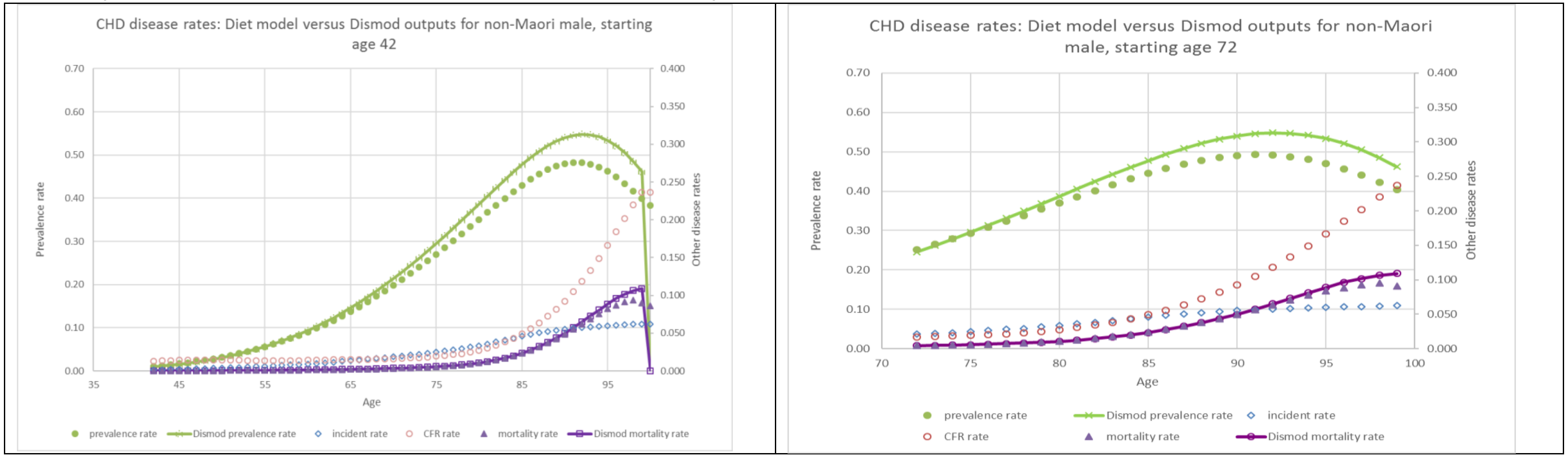

Figure 11: Stroke rates, by sex and ethnic group cohorts 2 years of age in 2011. DISMOD prevalence and mortality rates, and case fatality rates, are those inputted to the BODE[3] DIET MSLT; other rates are those outputted over the remainder of the cohort's life

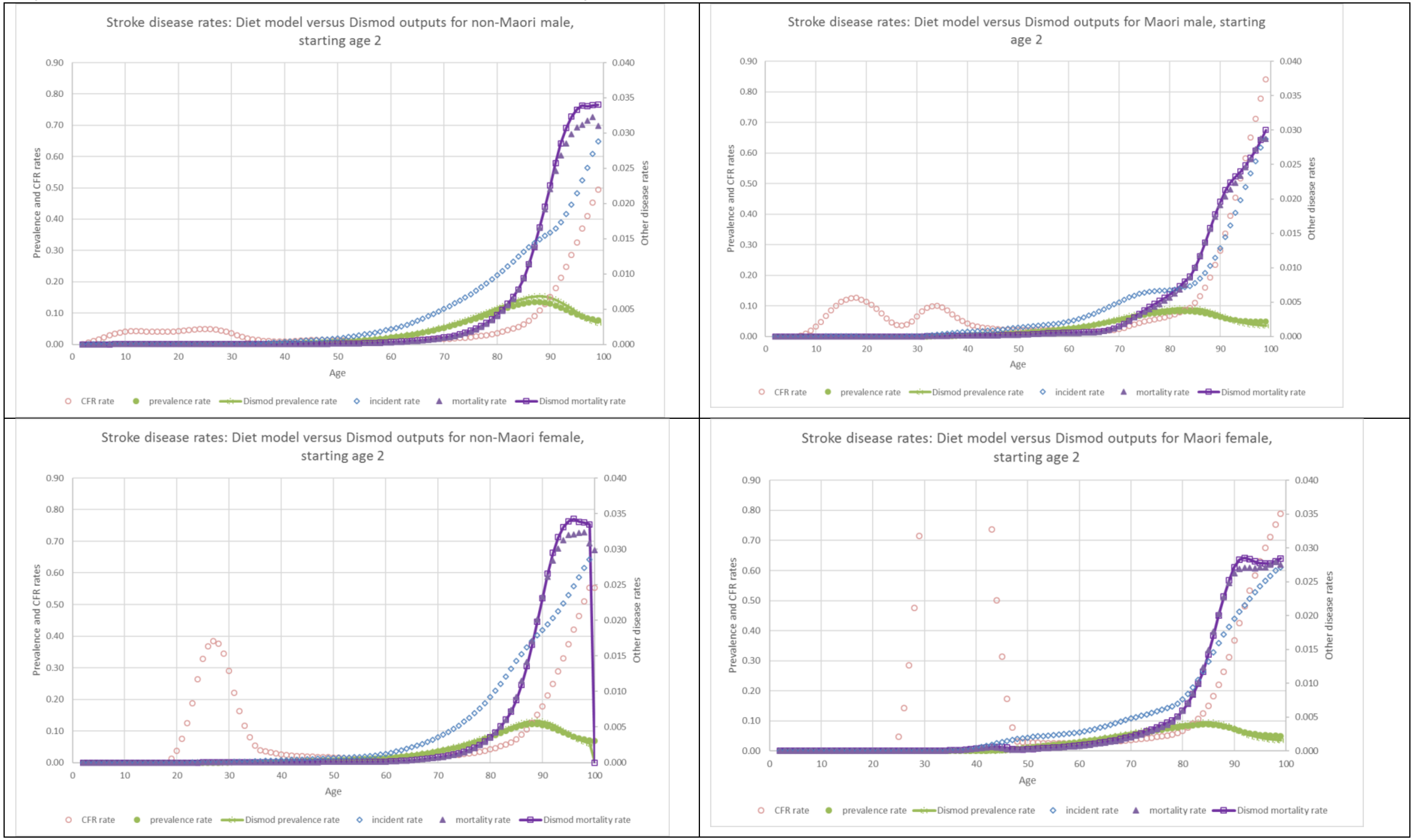

Figure 12: Stroke rates, for non-Māori males aged 42 and 72 years of age in 2011. DISMOD prevalence and mortality rates, and case fatality rates, are those inputted to the BODE3 DIET MSLT; other rates are those outputted over the remainder of the cohort’s life

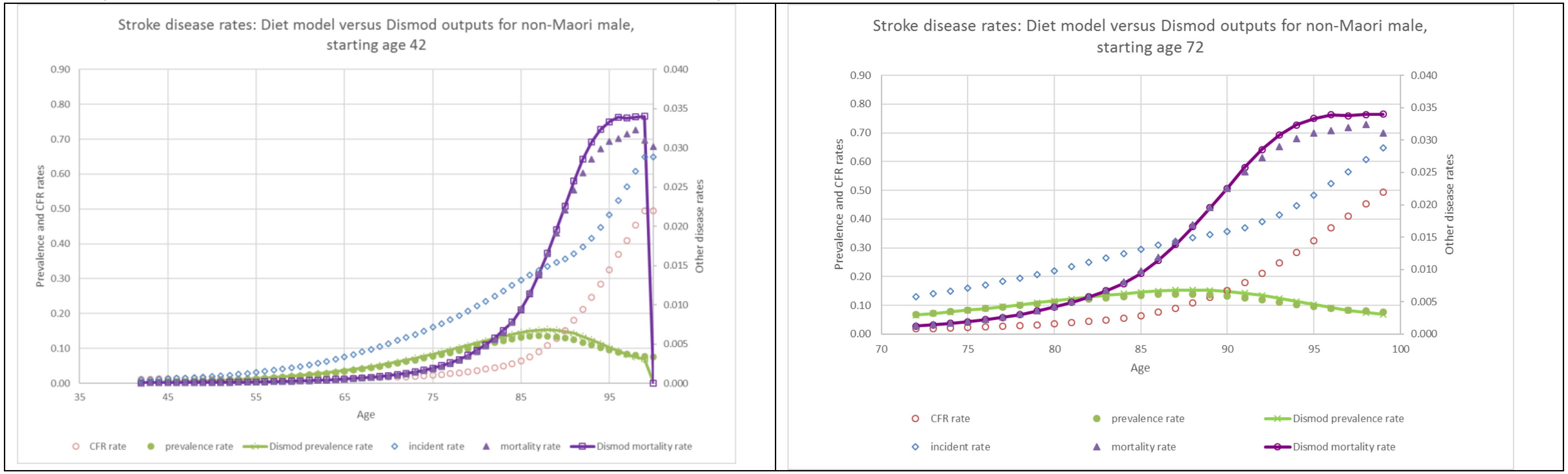

Figure 13: Diabetes rates, by sex and ethnic group cohorts 2 years of age in 2011. DISMOD prevalence and mortality rates, and case fatality rates, are those inputted to the BODE[3] DIET MSLT; other rates are those outputted over the remainder of the cohort's life

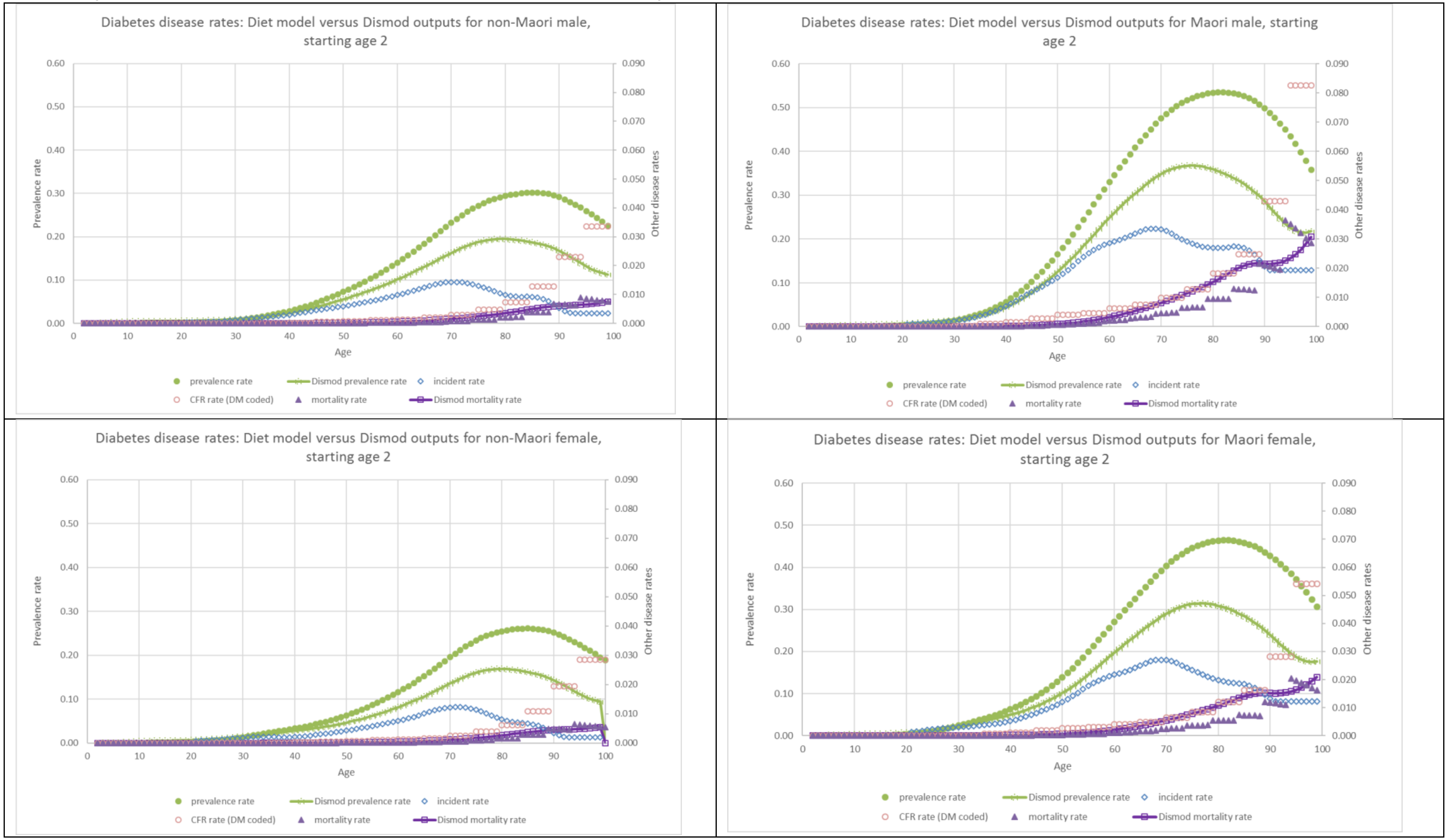

Figure 14: Diabetes rates, for non-Māori males aged 42 and 72 years of age in 2011. DISMOD prevalence and mortality rates, and case fatality rates, are those inputted to the BODE3 DIET MSLT; other rates are those outputted over the remainder of the cohort's life

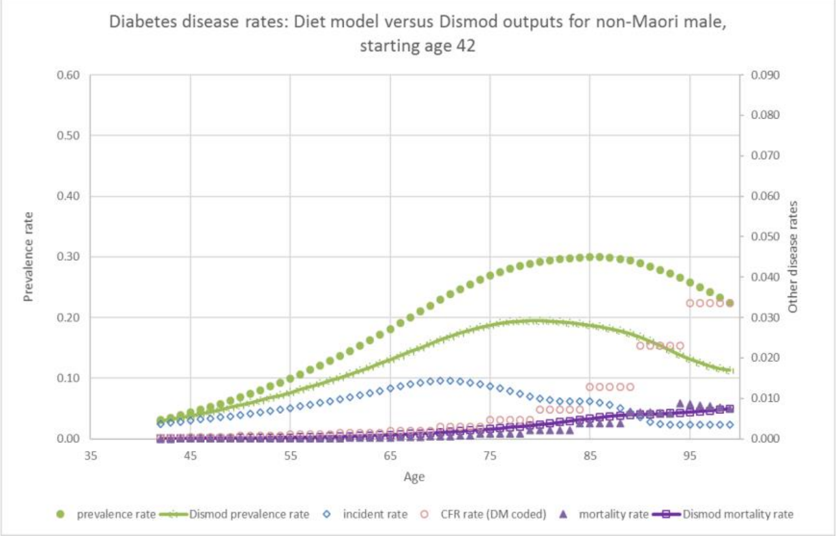

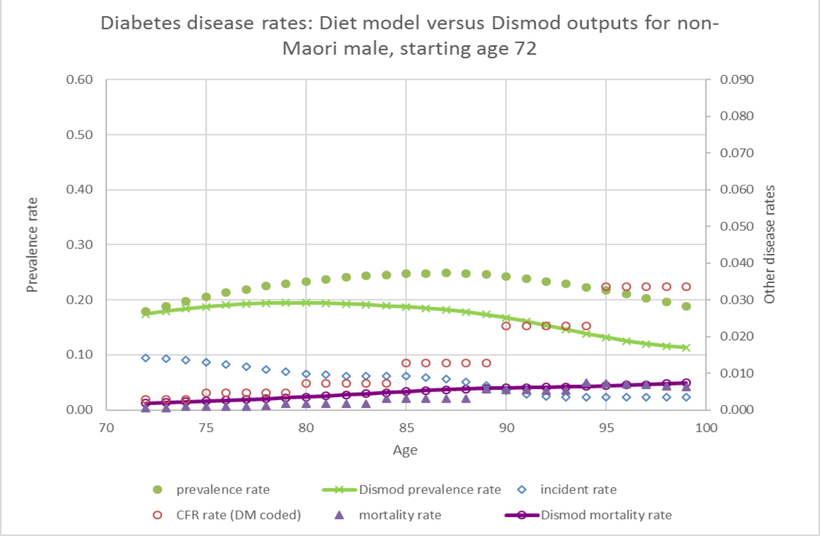

Section 3.04. Validation

The BODE^3 DIET MSLT model is a multi-application model, for studying preventive interventions. We attempted some validation given this broad remit. However, it was and is impossible to fully validate the model, both due to resource limitations and an absence of 'gold standard' data for many interventions (e.g. no randomized trials of saturated fat taxes through to disease incidence outcomes exist). Validation of the BODE^3 DIET MSLT model will continue alongside producing results (e.g. comparisons with overseas models), and new data will be forthcoming (e.g. disease incidence trends, intervention effect sizes). Thus, future improvements to the model are likely.

We organize this section using the headings from an International Society for Pharmacoeconomics and Outcomes Research (ISPOR) *Good Practices in Modelling Task Force* consensus paper:[48] face validity, verification (or internal validity), cross validity, external validity, and predictive validity.

3.04.1. Face Validity

"Face validity is the extent to which a model, its assumptions, and applications correspond to current science and evidence, as judged by people who have expertise in the problem."[48]

The BODE^3 DIET MSLT model follows the form and structure of a MSLT, and more specifically the Assessing Cost Effectiveness (ACE) Prevention models [37,50-58] (including dietary and Physical Activity models) and the BODE^3 Tobacco model [59,60]. These models have been peer reviewed many times, lending face validity.

Figure 1 (page 6) lays out the conceptual model for the BODE^3 DIET MSLT model – at least for the selected risk factors and pathways. It is – necessarily – a simplification of reality. For example:

- Other known risk factors (e.g. nuts and seeds) will likely be included in the future.
- As a macrosimulation model, it is difficult to allow for correlated risk factor distributions. That is, the BMI distribution is assumed independent of the fruit and vegetable intake distribution, across the population. Thus, the BODE^3 DIET MSLT model – unless modified or adapted – will be limited in answering questions around targeting of populations with multiple poor risk factors. The model also treats diseases as independent; no correlations in disease incidence for – say – CHD and lung cancer are allowed for. (Importantly – though – we do treat diabetes as both a risk factor and disease, allowing for the dependency of diabetes with CHD and stroke.)

We have not formally subjected the BODE^3 DIET MSLT model to external face validity review prior to submission of publications for scientific peer review.

3.04.2. Verification (or Internal Validity)

"Verification addresses whether the model's parts behave as intended and the model has been implemented correctly." [48]

A regular process of verification was and is used in modifying and extending the BODE^3 DIET MSLT model, namely:

- The following procedure was followed once model development was complete, checked and signed off by one of the Programme Directors: all model changes are undertaken by one team member, checked and signed off by a second team member, and signed off by one of the Programme Directors. This process accords with a Accountability for Quality Assurance process outlined by UK Department of Energy and Climate Change in their guidance for quality assurance of Excel-based models [61], and is documented more fully in a BODE[3] quality assurance protocol (forthcoming). All model builds and extensions are 'logged' in a 'readme' tab in the model.
- The following checks are implemented for a model version to be signed off:
    - A second team member – independently - randomly checking formulas and links in models
    - A second team member – independently – working through each process from beginning to end (e.g. risk factor A distribution, merged with risk factor A relative risks, to population impact fractions and their connection with disease incidence, then all-cause mortality, etc.).
- A series of sensitivity analyses are undertaken to logic (stress) test the model. This covers both extreme values and a likely range of values to check how the model responds. For example, trends in disease incidence rates are turned off, and compared against expectation, the results of this checking is signed off by a Programme Director. For stress testing, selected input parameters are changed to extreme values (e.g. turning disease incidences to zero, one by one) to ensure changes in model outputs are consistent with expectation.

The above and other BODE[3] quality assurance processes are documented more fully elsewhere,[62] as well as specifically to the BODE[3] DIET MSLT model in its Readme tab.

3.04.3. Cross Validity

"Cross-validation involves comparing a model with others and determining the extent to which they calculate similar results."[48]

Model comparisons within the BODE[3] Programme have occurred, and are proposed with other international groups.

Within the BODE[3] programme, identical dietary salt reduction interventions were run through an early iteration of the BODE[3] DIET MSLT model and a CVD model built in TreeAge that had previously been developed by BODE[3].[63,64] When an intervention of a decrease in sodium of 22.8mmol/day was run through both models, the overall QALYs gained were 110,000 in the TreeAge model and 103,000 in the DIET MSLT model (3% discounting). As there are a number of differences between the models generating results within 20% of each other was regarded as satisfactory, and the difference seen was closer to 5%. From our investigations it seems that the differences seen between the two models were due to a combination of different baseline incidence rates, baseline case-fatality rates and differing disability rates/weights between the two models. Model structure, definitions of stroke and effect size calculations don't appear to contribute very much to the differences seen.

Model comparisons are also underway with the Nuffield Department of Population Health, Oxford University (Adam Briggs, Peter Scarborough and colleagues) who are working on similar types of

models with similar food taxes and subsidy interventions (e.g. [65-67]). Model comparisons proposed include 'stripping back' to the same population demography and epidemiology to allow a head-to-head comparison of any differences in model structure, then sequential addition of varying population epidemiology (e.g. disease incidence rates, case-fatality and trends), and population demography (e.g. varying age structures).

3.04.4. External Validity

"In external validation, a model is used to simulate a real scenario, such as a clinical trial, and the predicted outcomes are compared with the real world ones." [48]

Randomized trials through to disease incidence for the interventions proposed to be modelled with the BODE[3] DIET MSLT model are rare. We will consider the relevance of one of these for such validation work: a major sodium reduction trial on health outcomes,[68] but we note this might not prove to be informative given the decline in CVD incidence over the 20 years of this trial.

Meta-analyses of trials (where available) are used for parameterizing intervention effect sizes in the model (e.g. association of mHealth on weight loss[69]).

'Natural experiments' – as they accrue (e.g. Danish food taxes[67,70] and Mexican SSB taxes[71]) – will also provide comparison points.

3.04.5. Predictive Validity

"Predictive validity involves using a model to forecast events and, after sometime, comparing the forecasted outcomes with the actual ones." [48]

It was not possible to compared forecast incidence and mortality rates in New Zealand for various *interventions* with model forecasts, as none of the interventions have been applied. However, it will be possible to compare BAU trends in disease incidence from the 2011 base-year out in due course.

Section 3.05. Model: Analysis

For each intervention, the model is run 2000 times using Monte Carlo simulation. Probabilistic uncertainty is included for intervention effect sizes (e.g. price elasticities, relative risks for the association between diet and disease incidence), intervention costs (e.g. cost of a new tax law) and selected baseline parameters (i.e. health system costs were assumed to have a gamma distribution with a SD of +/- 10%).

We included uncertainty in the annual percentage changes in selected disease incidence trends (see above) for the diseases that made the largest contribution to the QALYs gained (and hence also cost savings).

Uncertainty around the starting estimates of incidence and case-fatality has been included in the model. Year 2011 starting estimates have been assigned a log-normal distribution, SD of +/- 5%, with random draw in each iteration separately for incidence and case-fatality, by sex and age, but applied uniformly across ages (i.e. independent uncertainty by sex and age, but 100% correlated uncertainty by age within sex by ethnic groups).

All modelling is undertaken in Microsoft Excel®, using the add-in tool Ersatz (EpiGear, Version 1.3) for uncertainty analysis with R-software 'add-ons' for batch processing and output collation.

Section 3.06. A note on interchangeable use of 'DALYs averted' and 'QALYS gained'

Previous BODE[3] modelling[50] termed health gain as DALYs averted'. We use the terms 'DALYs averted' and 'QALYs gained' interchangeably. Why? Two reasons. First, The QALYs gained (or DALYs averted) in the MSLT modelling are _not_ the same as DALYs calculated in a BDS. In the BDS they are (usually) calculated in one cross-sectional year, as a shortfall against an ideal standard (e.g. the best sex-specific life-table mortality rates in the world). In BODE[3] (and other related MSLT modelling, e.g. ACE-Prevention [50]) the QALYs gained are the difference between the starting population's expectation of the remainder of their lives, and that under the intervention scenario. Second, the morbidity weights or 'health status valuations' (HSV) are pairwise comparisons conducted for the GBD [36], and as such are one variant of HSV used in routine economic evaluations and QALY estimation.[72] These morbidity weights – given their derivation for the GBD – are called disability weights.

The disability weighting (DW) (in this case DRs, which in term stem from DWs applied in the BDS itself) assigned is just one variant of health status valuation (HSVs); QALYs use a variety of HSVs (e.g. those from EQ5D, etc.). Furthermore, DALYs in the BDS use an external or reference life-table (to generate a health gap or loss measure); in this multi-state life-table, the DALYs averted are at the incremental margin for the 2011 New Zealand population, the same concept and method as used for QALYs. The only conceptual difference between the QALYs we calculate and the various QALYs presented in much other research, is the HSV metric. In other cost-utility analyses the source of HSV is likely to vary between studies (arguably to fit the population's preference, but more usually due to the pragmatics of different questionnaires used) whereas our QALYs are derived from one very large and coherent set of disability weights calculated in the GBD 2010 from multi-country surveys.[36] We do not claim that the HSV in our QALY is 'better' than that used in other QALY estimates – there is genuine uncertainty in all HSVs.

The QALY metric captures health gain (assuming the intervention is beneficial) that arises from a mix of change in years of life and quality of each year of life. Usually a gain in QALYs (in prevention interventions at least) is due to a gain in life years lived (with or without change in quality of life). Note, however, that it is possible to achieve QALY gains with a reduction in life years lived (but very good improvements in quality of life), or with an increase in life years gained that is greater than any 'penalty' from living in lower quality of life.

PART 4. References

1. Forouzanfar MH, Alexander L, Anderson HR, et al. Global, regional, and national comparative risk assessment of 79 behavioural, environmental and occupational, and metabolic risks or clusters of risks in 188 countries, 1990–2013: a systematic analysis for the Global Burden of Disease Study 2013. *The Lancet* 2015.
2. University of Otago MoH. The 2008/09 New Zealand Adult Nutrition Survey Data. In: Health; Mo, editor. Wellington; 2011.
3. Luiten CM, Steenhuis IH, Eyles H, Mhurchu CN, Waterlander WE. Ultra-processed foods have the worst nutrient profile, yet they are the most available packaged products in a sample of New Zealand supermarkets. *Public health nutrition* 2015: 1-9.
4. Blakely T, Foster R, Wilson N, BODE[3] Team. Burden of Disease Epidemiology, Equity and Cost-Effectiveness (BODE[3]) Study Protocol. Version 2.0. Wellington: Department of Public Health, University of Otago, Wellington 2012.
5. Mhurchu CN, Eyles H, Schilling C, et al. Food prices and consumer demand: differences across income levels and ethnic groups. *PloS one* 2013; 8(10): e75934.
6. Ni Mhurchu C, Eyles H, Genç M, et al. Effects of Health-Related Food Taxes and Subsidies on Mortality from Diet-Related Disease in New Zealand: An Econometric-Epidemiologic Modelling Study. *PLoS ONE [Electronic Resource]* 2015.
7. Nnoaham KE, Sacks G, Rayner M, Mytton O, Gray A. Modelling income group differences in the health and economic impacts of targeted food taxes and subsidies. *International Journal of Epidemiology* 2009; 38(5): 1324-33.
8. Cobiac L, Wa Tam K, Veerman L, Blakely T. Modelling the cost-effectiveness of taxes and subsidies for improving diet and population health. *Plos Med* 2017; 14(2): e1002232.
9. Andreyeva T, Long MW, Brownell KD. The impact of food prices on consumption: a systematic review of research on the price elasticity of demand for food. *American journal of public health* 2010; 100(2): 216-22.
10. Dharmasena S, Capps O. Intended and unintended consequences of a proposed national tax on sugar-sweetened beverages to combat the US obesity problem. *Health economics* 2012; 21(6): 669-94.
11. Smith TA, Lin B-H, Lee J-Y. Taxing caloric sweetened beverages: potential effects on beverage consumption, calorie intake, and obesity. *USDA-ERS Economic Research Report* 2010; (100).
12. Robert R CLH. Food Demand Analysis. Ames, Iowa. : Iowa State University Press; 1987 p. 7.
13. Edgerton DL. Weak separability and the estimation of elasticities in multistage demand systems. *American Journal of Agricultural Economics* 1997; 79(1): 62-79.
14. Sharma A, Hauck K, Hollingsworth B, Siciliani L. The effects of taxing sugar-sweetened beverages across different income groups. *Health economics* 2014; 23(9): 1159-84.
15. Tiffin R, Balcombe K, Salois M, Kehlbacher A. Estimating Food and Drink Elasticities. *University of Reading* 2011.
16. Nghiem N, Wilson N, Blakely T. Price elasticities for health economic modeling of food pricing intervention in Australia and New Zealand. . Wellington, NZ: Department of Public Health, University of Otago, 2011.
17. Ministry of Agriculture, Fisheries and Food. National Food Survey 2000: Annual Report on Food Expenditure, Consumption and Nutrient Intakes. London: Her Majesty's Stationery Office, 2000.
18. Ulubasoglu M, Mallick D, Wadud M, Hone P, Haszler H. Food Demand Elasticities in Australia. 2010. http://www.deakin.edu.au/buslaw/aef/workingpapers/papers/2010_17.pdf4/5/2011).
19. Seale J, Jr. AR, Bernstein JA. International Evidence on Food Consumption Patterns: USDA, 2005.
20. Wirsenius S, Hedenus F, Mohlin K. Greenhouse gas taxes on animal food products: rationale, tax scheme and climate mitigation effects. *Climatic Change* 2011; 108(1): 159-84.

21. Reed AJ, Levedahl JW, Hallahan C. The generalized composite commodity theorem and food demand estimation. *Am J Agr Econ* 2005; 87(1): 28-37.
22. Tiffin R, Arnoult M. Bayesian estimation of the infrequency of purchase model with an application to food demand in the UK. 2008. http://mpra.ub.uni-muenchen.de/18836/.
23. Cornelsen L, Green R, Turner R, et al. What Happens to Patterns of Food Consumption when Food Prices Change? Evidence from A Systematic Review and Meta-Analysis of Food Price Elasticities Globally. *Health Econ* 2015; 24(12): 1548-59.
24. Green R, Cornelsen L, Dangour AD, et al. The effect of rising food prices on food consumption: systematic review with meta-regression. *BMJ* 2013; 346: f3703.
25. Zhen C, Finkelstein EA, Nonnemaker J, Karns S, Todd JE. Predicting the Effects of Sugar-Sweetened Beverage Taxes on Food and Beverage Demand in a Large Demand System. *Am J Agric Econ* 2014; 96(1): 1-25.
26. Hansen J, Brooks N. Commodity and Food Elasticities: Demand Elasticities from Literature. In: USDA, editor.; 2009.
27. Michelini C, Chatterjee S. Demographic variables in demand systems: An analysis of New Zealand household expenditure 1984–1992. *New Zealand Economic Papers* 1997; 31(2): 153-73.
28. Michelini C. New Zealand household consumption patterns 1983–1992: An application of the almost-ideal-demand-system. *New Zealand Economic Papers* 1999; 33(2): 15-26.
29. Banks J, Blundell R, Lewbel A. Quadratic Engel curves and consumer demand. *Review of Economics and statistics* 1997; 79(4): 527-39.
30. Klonaris S, Hallam D. Conditional and unconditional food demand elasticities in a dynamic multistage demand system. *Applied Economics* 2003; 35(5): 503-14.
31. Huang KS, Huang SW. Consumer welfare effects of increased food and energy prices. *Applied Economics* 2011; 44(19): 2527-36.
32. Brännlund R, Ghalwash T, Nordström J. Increased energy efficiency and the rebound effect: Effects on consumption and emissions. *Energy Economics* 2007; 29(1): 1-17.
33. Aepli M. Estimation of elasticities for food in Switzerland: Final results. *Report for the Swiss Federal Office for Agriculture (BLW)* 2014.
34. Hall KD, Sacks G, Chandramohan D, et al. Quantification of the effect of energy imbalance on bodyweight. *The Lancet* 2011; 378(9793): 826-37.
35. Ministry of Health. Health Loss in New Zealand: A report from the New Zealand Burden of Diseases, Injuries and Risk Factors Study, 2006–2016. Wellington: Ministry of Health, 2013.
36. Salomon JA, Vos T, Hogan DR, et al. Common values in assessing health outcomes from disease and injury: disability weights measurement study for the Global Burden of Disease Study 2010. *Lancet* 2012; 380(9859): 2129-43.
37. Cobiac LJ, Vos T, Barendregt JJ. Cost-Effectiveness of Interventions to Promote Physical Activity: A Modelling Study. *PLoS Med* 2009; 6(7): e1000110.
38. Forouzanfar MH, Alexander L, Anderson HR, et al. Global, regional, and national comparative risk assessment of 79 behavioural, environmental and occupational, and metabolic risks or clusters of risks in 188 countries, 1990–2013: a systematic analysis for the Global Burden of Disease Study 2013. *The Lancet* 2015; 386(10010): 2287-323.
39. Lim SS, Vos T, Flaxman AD, et al. A comparative risk assessment of burden of disease and injury attributable to 67 risk factors and risk factor clusters in 21 regions, 1990–2010: a systematic analysis for the Global Burden of Disease Study 2010. *The lancet* 2013; 380(9859): 2224-60.
40. Barendregt JJ, Veerman JL. Categorical versus continuous risk factors and the calculation of potential impact fractions. *J Epidemiol Community Health* 2010; 64(3): 209-12.
41. Morgenstern H, Bursic ES. A method for using epidemiologic data to estimate the potential impact of an intervention on the health status of a target population. *J Community Health* 1982; 7(4): 292-309.
42. Dansinger ML, Tatsioni A, Wong JB, Chung M, Balk EM. Meta-analysis: the effect of dietary counseling for weight loss. *Annals of internal medicine* 2007; 147(1): 41-50.

43. VanderWeele TJ. Explanation in Causal Inference: Methods for Mediation and Interaction: Oxford University Press; 2015.
44. Blakely T, Foster R, Wilson N, Team B. Burden of Disease Epidemiology, Equity and Cost-Effectiveness (BODE3) Study Protocol. Wellington: Department of Public Health, University of Otago, 2012.
45. Barendregt J, Oortmarssen GJ, Vos T, Murray CJL. A generic model for the assessment of disease epidemiology: the computational basis of DisMod II. *Popul Health Metr* 2003; 1(1): 4.
46. Costilla R, Atkinson J, T B. Incorporating Ethnic and Deprivation Variation to Cancer Incidence Estimates over 2006-2026 for ABC-CBA. Wellington: Department of Public Health, University of Otago, 2011.
47. Ministry of Health. Ways and Means: A report on methodology from the New Zealand Burden of Disease, Injury and Risk Study, 2006 - 2016. Wellington: Ministry of Health, 2013.
48. Eddy DM, Hollingworth W, Caro JJ, Tsevat J, McDonald KM, Wong JB. Model Transparency and Validation: A Report of the ISPOR-SMDM Modeling Good Research Practices Task Force-7. *Value in Health* 2012; 15(6): 843-50.
49. Weinstein MC, O'Brien B, Hornberger J, et al. Principles of Good Practice for Decision Analytic Modeling in Health-Care Evaluation: Report of the ISPOR Task Force on Good Research Practices—Modeling Studies. *Value in Health* 2003; 6(1): 9-17.
50. Vos T, Carter R, Barendregt J, et al. Assessing Cost-Effectiveness in the Prevention (Ace-Prevention): Final Report. Brisbane and Melbourne: University of Queensland and Deakin University, 2010.
51. Bertram M, Lim S, Barendregt J, Vos T. Assessing the cost-effectiveness of drug and lifestyle intervention following opportunistic screening for pre-diabetes in primary care. *Diabetologia* 2010; 53(5): 875-81.
52. Byrnes J, Cobiac L, Doran C, Vos T, Shakeshaft A. Cost-effectiveness of volumetric alcohol taxation in Australia. *Medical Journal of Australia* 2010; 192(8): 439-43.
53. Cobiac L, Magnus A, Barendregt J, Carter R, Vos T. Improving the cost-effectiveness of cardiovascular disease prevention in Australia: a modelling study. *BMC Public Health* 2012; 12(1): 398.
54. Cobiac L, Vos T, Doran C, Wallace A. Cost-effectiveness of interventions to prevent alcohol-related disease and injury in Australia. *Addiction* 2009; 104(10): 1646-55.
55. Cobiac LJ, Vos T, Veerman JL. Cost-effectiveness of interventions to reduce dietary salt intake. *Heart* 2010; 96(23): 1920-5.
56. Cobiac LJ, Vos T, Veerman L. Cost-effectiveness of Weight Watchers and the Lighten Up to a Healthy Lifestyle program. *Aust N Z J Public Health* 2010; 34: 240-7.
57. van den Berg M, Smit F, Vos T, van Baal PH. Cost-effectiveness of opportunistic screening and minimal contact psychotherapy to prevent depression in primary care patients. *PLoS One* 2011; 6(8): e22884.
58. Veerman JL, Barendregt JJ, Forster M, Vos T. Cost-Effectiveness of Pharmacotherapy to Reduce Obesity. *PLoS One* 2011; 6(10): e26051.
59. Blakely T, Cobiac LJ, Cleghorn CL, et al. Health, Health Inequality, and Cost Impacts of Annual Increases in Tobacco Tax: Multistate Life Table Modeling in New Zealand. *PLoS Med* 2015; 12(7): e1001856.
60. Pearson AL, Cleghorn CL, van der Deen FS, et al. Tobacco retail outlet restrictions: health and cost impacts from multistate life-table modelling in a national population. *Tob Control* 2016.
61. DECC Modelling Integrity Team. Quality Assurance: Guidance for Models. London: Department of Energy and Climate Change, UK, 2015.
62. Wilson N, Blakely T, Nghiem N, et al. Guide for Ensuring High Quality of Modelling Outputs in the BODE3 Programme, 2017.

63. Nghiem N, Blakely T, Cobiac LJ, Cleghorn CL, Wilson N. The health gains and cost savings of dietary salt reduction interventions, with equity and age distributional aspects. *BMC Public Health* 2016; 16(1): 423.
64. Nghiem N, Blakely T, Cobiac LJ, Pearson AL, Wilson N. Health and Economic Impacts of Eight Different Dietary Salt Reduction Interventions. *PLoS One* 2015; 10(4): e0123915.
65. Briggs AD, Mytton OT, Kehlbacher A, Tiffin R, Rayner M, Scarborough P. Overall and income specific effect on prevalence of overweight and obesity of 20% sugar sweetened drink tax in UK: econometric and comparative risk assessment modelling study. *BMJ* 2013; 347: f6189.
66. Briggs ADM, Wolstenholme J, Blakely T, Scarborough P. Choosing an epidemiological model structure for the economic evaluation of non-communicable disease public health interventions. *Population Health Metrics* 2016; 14(1).
67. Smed S, Scarborough P, Rayner M, Jensen J. The effects of the Danish saturated fat tax on food and nutrient intake and modelled health outcomes: an econometric and comparative risk assessment evaluation. *European Journal of Clinical Nutrition* 2016.
68. Cook NR, Appel LJ, Whelton PK. Sodium Intake and All-Cause Mortality Over 20 Years in the Trials of Hypertension Prevention. *J Am Coll Cardiol* 2016; 68(15): 1609-17.
69. Lyzwinski LN. A systematic review and meta-analysis of mobile devices and weight loss with an intervention content analysis. *Journal of personalized medicine* 2014; 4(3): 311-85.
70. Jensen JD, Smed S. The Danish tax on saturated fat – Short run effects on consumption, substitution patterns and consumer prices of fats. *Food Policy* 2013; 42: 18-31.
71. Colchero MA, Popkin BM, Rivera JA, Ng SW. Beverage purchases from stores in Mexico under the excise tax on sugar sweetened beverages: observational study. *BMJ* 2016; 352: h6704.
72. Brazier J, Ratclife J, Salomon J, Tsuchiya A. Measuring and valuing health benefits for economic evaluation. New York: Oxford University Press; 2007.
73. Ministry of Health. Cancer Mortality Projections 2000–2004 update. Wellington 2008.
74. Blakely T CR, Soeberg M. Cancer excess mortality rates over 2006-2026 for ABC-CBA. Wellington Department of Public Health, University of Otago, Wellington, 2012.
75. Peters SA, Huxley RR, Woodward M. Diabetes as a risk factor for stroke in women compared with men: a systematic review and meta-analysis of 64 cohorts, including 775 385 individuals and 12 539 strokes. *The Lancet* 2014; 383(9933): 1973-80.
76. Peters SA, Huxley RR, Woodward M. Diabetes as risk factor for incident coronary heart disease in women compared with men: a systematic review and meta-analysis of 64 cohorts including 858,507 individuals and 28,203 coronary events. Springer; 2014.
77. Steel N. Global, regional, and national comparative risk assessment of 79 behavioural, environmental and occupational, and metabolic risks or clusters of risks, 1990–2015: a systematic analysis for the Global Burden of Disease Study 2015. *The Lancet* 2016; 388(10053): 1659-724.

Appendix A: Food groups from the NZANS used in the BODE³ intervention model

Table 11: Food groups from the NZANS used in the BODE³ intervention model

01.00.00	Grains and Pasta	01.01.01	White rice(includes parboiled & basmati)
01.00.00	Grains and Pasta	01.01.02	Brown rice
01.00.00	Grains and Pasta	01.01.03	Fried rice/risotto/pilaff/rice salad/sushi
01.00.00	Grains and Pasta	01.01.04	Rice products (e.g. rice wafers/cakes)
01.00.00	Grains and Pasta	01.02.01	Wheat flour-white
01.00.00	Grains and Pasta	01.02.02	Wheat flour-wholemeal
01.00.00	Grains and Pasta	01.02.03	Other flours (rice, corn, rye, arrowroot, oat, barley etc.)
01.00.00	Grains and Pasta	01.03.01	Egg Noodles
01.00.00	Grains and Pasta	01.03.02	Plain pasta
01.00.00	Grains and Pasta	01.03.03	Filled pasta (e.g. ravioli)
01.00.00	Grains and Pasta	01.03.04	Noodles (includes Asian style noodles, 2
01.00.00	Grains and Pasta	01.04.01	Wheat bran
01.00.00	Grains and Pasta	01.04.02	Oat bran
01.00.00	Grains and Pasta	01.04.03	Other brans
01.00.00	Grains and Pasta	01.04.04	Wheat germ
01.00.00	Grains and Pasta	01.05.01	Pasta and sauce, and other cereal based dishes e.g. lasagne
01.00.00	Grains and Pasta	01.06.01	Other grains and cereals
02.00.00	Bread (includes rolls and speciality breads)	02.01.01	White
02.00.00	Bread (includes rolls and speciality breads)	02.01.02	Fibre white
02.00.00	Bread (includes rolls and speciality breads)	02.01.03	Wholemeal
02.00.00	Bread (includes rolls and speciality breads)	02.01.04	Mixed grain
02.00.00	Bread (includes rolls and speciality breads)	02.01.05	Rye and heavy types
02.00.00	Bread (includes rolls and speciality breads)	02.01.06	Corn bread
02.00.00	Bread (includes rolls and speciality breads)	02.01.07	Fruit bread
02.00.00	Bread (includes rolls and speciality breads)	02.01.08	Wheatmeal
02.00.00	Bread (includes rolls and speciality breads)	02.02.01	Flat bread, Pita bread, tortillas (plain), pizza bases
02.00.00	Bread (includes rolls and speciality breads)	02.03.01	Garlic breads
02.00.00	Bread (includes rolls and speciality breads)	02.03.02	Cheese/tomato/pizza topped breads
02.00.00	Bread (includes rolls and speciality breads)	02.03.03	Flavoured breads
02.00.00	Bread (includes rolls and speciality breads)	02.03.04	Other
02.00.00	Bread (includes rolls and speciality breads)	02.04.01	Bagels
02.00.00	Bread (includes rolls and speciality breads)	02.05.01	English muffins and crumpets

02.00.00	Bread (includes rolls and speciality breads)	02.06.01	Sweet yeast buns includes iced buns and buns with sweet fillings; cream, custard
02.00.00	Bread (includes rolls and speciality breads)	02.07.01	Other breads
03.00.00	Breakfast cereals	03.01.01	Wheat based biscuits and shredded wheat with fruits/nuts
03.00.00	Breakfast cereals	03.01.02	Wheat based biscuits and shredded wheat without fruit/nuts
03.00.00	Breakfast cereals	03.02.01	Single cereal, puffed/flaked - sweetened
03.00.00	Breakfast cereals	03.02.02	Single cereal, puffed/flaked - unsweetened
03.00.00	Breakfast cereals	03.02.03	Mixed cereal, puffed/flaked - sweetened
03.00.00	Breakfast cereals	03.02.04	Mixed cereal, puffed/flaked - unsweetened
03.00.00	Breakfast cereals	03.03.01	Single and multigrain extruded cereals - sweetened
03.00.00	Breakfast cereals	03.03.02	Single and multigrain extruded cereal - unsweetened
03.00.00	Breakfast cereals	03.04.01	Single and multigrain porridge and cooked cereals with fruit/nuts
03.00.00	Breakfast cereals	03.04.02	Single and multigrain porridge and cooked cereals without fruit/nuts
03.00.00	Breakfast cereals	03.05.01	Single and multigrain bran based cereals with fruit/nuts
03.00.00	Breakfast cereals	03.05.02	Single and multigrain bran based cereals without fruit/nuts
03.00.00	Breakfast cereals	03.06.01	Toasted muesli - sweetened
03.00.00	Breakfast cereals	03.06.02	Toasted muesli - unsweetened
03.00.00	Breakfast cereals	03.07.01	Untoasted muesli - sweetened
03.00.00	Breakfast cereals	03.07.02	Untoasted muesli - unsweetened
04.00.00	Biscuits	04.01.01	Plain
04.00.00	Biscuits	04.01.02	Chocolate coated or chocolate chip
04.00.00	Biscuits	04.01.03	Fruit filled
04.00.00	Biscuits	04.01.04	Cream filled or with icing/dipping sauce
04.00.00	Biscuits	04.01.05	Biscuits with fruit and/or nuts
04.00.00	Biscuits	04.02.01	single or multi grain base - low fat (<=5% fat)
04.00.00	Biscuits	04.02.02	single or multi grain base - medium and high fat (>5% fat)
05.00.00	Cakes and muffins	05.01.01	Plain includes fruit (includes fruit cakes and sultana cakes) &plain cakes
05.00.00	Cakes and muffins	05.02.01	Sponge includes plain sponges and sponges with fillings
05.00.00	Cakes and muffins	05.03.01	Slices
05.00.00	Cakes and muffins	05.04.01	Cake type desserts/gateaux includes fancy rich cakes and gateaux
05.00.00	Cakes and muffins	05.05.01	Sweet muffins
05.00.00	Cakes and muffins	05.06.01	Savoury muffins
05.00.00	Cakes and muffins	05.07.01	Low fat and bran muffins, scones, pancakes, pikelets, waffles
05.00.00	Cakes and muffins	05.08.01	Doughnuts includes plain doughnut and doughnuts with cream and/or jam
05.00.00	Cakes and muffins	05.09.01	Pastry includes croissant, danish and other sweet pastries, plain pastries
05.00.00	Cakes and muffins	05.10.01	Cake bars
06.00.00	Bread based dishes	06.01.01	Sandwiches, filled rolls, filled pita breads and croissants
06.00.00	Bread based dishes	06.02.01	Burgers and hot dogs
06.00.00	Bread based dishes	06.03.01	Pizza
06.00.00	Bread based dishes	06.04.01	Tortilla, tacos, doner kebabs, burritos, nachos
06.00.00	Bread based dishes	06.05.01	Dim sims, spring rolls, wontons, bread based batters

06.00.00	Bread based dishes	06.06.01	Stuffings (bread based)
06.00.00	Bread based dishes	06.07.01	Other products
07.00.00	Puddings/desserts	07.01.01	Milk puddings includes rice pudding, instant puddings, custards and trifle
07.00.00	Puddings/desserts	07.02.01	Cheesecakes
07.00.00	Puddings/desserts	07.03.01	Fruit crumbles
07.00.00	Puddings/desserts	07.04.01	Mousse
07.00.00	Puddings/desserts	07.05.01	Sponge (steamed)
07.00.00	Puddings/desserts	07.06.01	Sweet pies e.g. fruit or custard pies
07.00.00	Puddings/desserts	07.07.01	Pavlova & meringues
07.00.00	Puddings/desserts	07.08.01	Other puddings
08.00.00	Milk	08.01.01	Whole fluid
08.00.00	Milk	08.01.02	Homogenised fluid (blue)
08.00.00	Milk	08.01.03	Semi trim (light blue)
08.00.00	Milk	08.01.04	Trim (green)
08.00.00	Milk	08.01.05	Calcium enriched fluid
08.00.00	Milk	08.02.01	Goats milks
08.00.00	Milk	08.03.01	Evaporated/Condensed undiluted
08.00.00	Milk	08.03.03	Milk powder-low fat
08.00.00	Milk	08.03.04	Milk powder-regular
08.00.00	Milk	08.04.01	Milkshakes
08.00.00	Milk	08.05.01	Flavoured milk
08.00.00	Milk	08.05.02	Flavoured milk-calcium enriched
08.00.00	Milk	08.06.01	Soy milk
08.00.00	Milk	08.07.01	Infant formula
08.00.00	Milk	08.08.01	Other milk
09.00.00	Dairy products	09.01.01	Cream-regular
09.00.00	Dairy products	09.01.02	Cream-reduced fat
09.00.00	Dairy products	09.02.01	Sour cream-regular
09.00.00	Dairy products	09.02.02	Sour cream-reduced fat
09.00.00	Dairy products	09.03.01	Ice cream-regular
09.00.00	Dairy products	09.03.02	Ice cream-rich varieties
09.00.00	Dairy products	09.03.03	Ice cream-reduced fat/frozen confectionery
09.00.00	Dairy products	09.03.05	Novelty ice cream
09.00.00	Dairy products	09.04.01	Yoghurt-regular
09.00.00	Dairy products	09.04.02	Yoghurt-reduced fat
09.00.00	Dairy products	09.04.03	Dairy foods
09.00.00	Dairy products	09.04.04	Yoghurt-Low/non fat
09.00.00	Dairy products	09.04.05	Frozen yoghurt
09.00.00	Dairy products	09.04.06	Yoghurt-High fat
09.00.00	Dairy products	09.04.07	Yoghurt-Soy
09.00.00	Dairy products	09.04.08	Fromage frais
09.00.00	Dairy products	09.05.01	Other dairy products
09.00.00	Dairy products	09.06.01	Dairy based dips

10.00.00	Cheese	10.01.01	High fat cheese (>30g fat/100g) blue cheeses, cheddar, cream cheese, colby
10.00.00	Cheese	10.02.01	Medium fat cheese (20-30g fat/100g) edam, processed cheese, cheese spread
10.00.00	Cheese	10.03.01	Low fat cheese (<20g fat/100g) includes cottage cheese, quark, ricotta, mozzarella
10.00.00	Cheese	10.04.01	Other
11.00.00	Butter and Margarine	11.01.01	Butter
11.00.00	Butter and Margarine	11.02.01	Butter/margarine blends
11.00.00	Butter and Margarine	11.03.01	Polyunsaturated margarine (approximately 70%)-includes flavoured margarine
11.00.00	Butter and Margarine	11.04.01	Monounsaturated margarine
11.00.00	Butter and Margarine	11.05.01	Butter - reduced fat
11.00.00	Butter and Margarine	11.05.02	Polyunsaturated margarine - reduced fat
11.00.00	Butter and Margarine	11.05.03	Monounsaturated margarine - reduced fat
12.00.00	Fats and oils	12.01.01	High SAFA/MUFA includes beef dripping, lard, chefade, palm oil, suet
12.00.00	Fats and oils	12.02.01	Coconut oil (high SAFA) includes coconut oil, shortening from coconut oil
12.00.00	Fats and oils	12.03.01	High MUFA/PUFA includes sesame oil, corn oil
12.00.00	Fats and oils	12.04.01	High MUFA includes canola shortening, canola oil, peanut oil
12.00.00	Fats and oils	12.04.02	Olive oil
12.00.00	Fats and oils	12.05.01	High PUFA includes sunflower oil, soybean oil, safflower oil
12.00.00	Fats and oils	12.06.01	Oil blends and other oils (includes salad/cooking oil, frying oil and vegetable
13.00.00	Eggs and egg dishes	13.01.01	Poached, boiled and fried eggs
13.00.00	Eggs and egg dishes	13.02.01	Scrambled eggs and omelettes with cheese and other additions
13.00.00	Eggs and egg dishes	13.02.02	Self-crusting quiches
13.00.00	Eggs and egg dishes	13.02.03	Eggs with additions (scrambled eggs and omelettes with fat/milk)
13.00.00	Eggs and egg dishes	13.02.04	Egg stir-fry and egg foo yung
14.00.00	Beef and Veal	14.01.01	Muscle meat includes steak, roast, schnitzel, corned beef, mince & other muscle
14.00.00	Beef and Veal	14.02.01	Casseroles/stews with gravy sauce and vegetables/cereals/pasta
14.00.00	Beef and Veal	14.02.02	Casseroles/stews with tomato based sauce and vegetables/cereals/pasta
14.00.00	Beef and Veal	14.02.03	Casseroles/stews with sauce only
14.00.00	Beef and Veal	14.02.04	Casseroles/stews with cream based sauce and vegetables/cereals/pasta
14.00.00	Beef and Veal	14.03.01	Stir-fries with beef & sauce only
14.00.00	Beef and Veal	14.03.02	Stir-fries with beef, sauce & vegetables
14.00.00	Beef and Veal	14.03.03	Stir-fries with beef, sauce & rice/noodles
14.00.00	Beef and Veal	14.03.04	Stir-fries with beef, sauce & vegetables and rice/noodles
15.00.00	Lamb/Mutton	15.01.01	Muscle meats includes roast, chops, steak, mince & other muscle meats
15.00.00	Lamb/Mutton	15.02.01	Casseroles/stews with sauce only
15.00.00	Lamb/Mutton	15.02.02	Casseroles/stews with gravy sauce and vegetables/cereals/pasta

15.00.00	Lamb/Mutton	15.02.03	Casseroles/stews with tomato based sauce and vegetables/cereals/pasta
15.00.00	Lamb/Mutton	15.03.01	Stir-fries with meat and sauce only
15.00.00	Lamb/Mutton	15.03.02	Stir-fries with meat, sauce and vegetables
15.00.00	Lamb/Mutton	15.03.03	Stir-fries with meat, sauce, vegetables and rice/noodles
15.00.00	Lamb/Mutton	15.03.04	Stir-fries with meat, sauce and rice/noodles
16.00.00	Pork	16.01.01	Bacon
16.00.00	Pork	16.02.01	Ham
16.00.00	Pork	16.03.01	Pork muscle meat includes roast, chops. steak, mince, schnitzel, strips
16.00.00	Pork	16.04.01	Casseroles/stews with sauce only
16.00.00	Pork	16.04.02	Casseroles/stews with gravy sauce and vegetables/cereals/pasta
16.00.00	Pork	16.04.03	Casseroles/stews with tomato based sauce and vegetables/cereals/pasta
16.00.00	Pork	16.05.01	Stir-fries with meat and sauce only
16.00.00	Pork	16.05.02	Stir-fries with meat, sauce and vegetables
16.00.00	Pork	16.05.03	Stir-fries with meat, sauce, vegetables and rice/noodles
16.00.00	Pork	16.05.04	Stir-fries with meat, sauce and rice/noodles
17.00.00	Poultry	17.01.01	Chicken muscle meats includes breast, drum, thigh, wing, mince
17.00.00	Poultry	17.02.01	Chicken processed meat includes nuggets, patty/fingers, roll processed meats
17.00.00	Poultry	17.03.01	Casseroles/stews with sauce only
17.00.00	Poultry	17.03.02	Casseroles/stews with gravy sauce and vegetables/cereals/pasta
17.00.00	Poultry	17.03.03	Casseroles/stews with tomato based sauce and vegetables/cereals/pasta
17.00.00	Poultry	17.03.04	Casseroles/stews with cream based sauce and vegetables/cereals/pasta
17.00.00	Poultry	17.04.01	Stir-fries with meat & sauce only
17.00.00	Poultry	17.04.02	Stir-fries with meat, sauce & vegetables
17.00.00	Poultry	17.04.03	Stir-fries with meat, sauce & vegetables and rice/noodles
17.00.00	Poultry	17.04.04	Stir-fries with meat, sauce and rice/noodles
17.00.00	Poultry	17.05.01	Duck muscle meats
17.00.00	Poultry	17.05.02	Casserole
17.00.00	Poultry	17.06.01	Turkey muscle meats
17.00.00	Poultry	17.06.02	Turkey processed meats
17.00.00	Poultry	17.07.01	Other poultry
18.00.00	Other meat	18.01.01	Venison includes muscle meats, casseroles and sausages
18.00.00	Other meat	18.02.01	Rabbit/Hare
18.00.00	Other meat	18.03.01	Tongue
18.00.00	Other meat	18.03.02	Black and white pudding
18.00.00	Other meat	18.03.03	Kidney
18.00.00	Other meat	18.03.04	Sheep Heart
18.00.00	Other meat	18.03.05	Liver
18.00.00	Other meat	18.03.06	Pate (made from liver)
18.00.00	Other meat	18.03.07	Other offal

18.00.00	Other meat	18.04.01	Goat
18.00.00	Other meat	18.05.01	Ostrich
18.00.00	Other meat	18.06.01	Kangaroo/crocodile
19.00.00	Sausages and processed meats	19.01.01	Sausages
19.00.00	Sausages and processed meats	19.01.02	Sausages, chicken
19.00.00	Sausages and processed meats	19.01.03	Sausages, vegetarian
19.00.00	Sausages and processed meats	19.02.01	Luncheon
19.00.00	Sausages and processed meats	19.03.01	Frankfurters
19.00.00	Sausages and processed meats	19.04.01	Saveloys
19.00.00	Sausages and processed meats	19.04.02	Battered saveloys
19.00.00	Sausages and processed meats	19.05.01	Salami
19.00.00	Sausages and processed meats	19.06.01	Meat-loaf
19.00.00	Sausages and processed meats	19.07.01	Meat patties
20.00.00	Pies and pasties	20.01.01	Beef pies includes pies with pastry, potato topped pies
20.00.00	Pies and pasties	20.02.01	Chicken pies includes pies with pastry, potato topped pies
20.00.00	Pies and pasties	20.03.01	Pasties
20.00.00	Pies and pasties	20.04.01	Savouries
20.00.00	Pies and pasties	20.05.01	Sausage rolls
20.00.00	Pies and pasties	20.06.01	Bacon and egg pie
20.00.00	Pies and pasties	20.07.01	Quiche
20.00.00	Pies and pasties	20.08.01	Other pies includes seafood pies, mutton pies, vegetarian pies
21.00.00	Fish/Seafood	21.01.01	Battered fin fish
21.00.00	Fish/Seafood	21.01.02	Battered shell fish
21.00.00	Fish/Seafood	21.02.01	Canned smoked fish
21.00.00	Fish/Seafood	21.02.02	Canned sardines
21.00.00	Fish/Seafood	21.02.03	Canned tuna,
21.00.00	Fish/Seafood	21.02.04	Canned salmon
21.00.00	Fish/Seafood	21.02.05	Canned shellfish
21.00.00	Fish/Seafood	21.02.06	Canned other
21.00.00	Fish/Seafood	21.03.01	Fin fish includes smoked , frozen and fresh
21.00.00	Fish/Seafood	21.04.01	Mussels,
21.00.00	Fish/Seafood	21.04.02	Oysters,
21.00.00	Fish/Seafood	21.04.03	Paua
21.00.00	Fish/Seafood	21.04.04	Scallops
21.00.00	Fish/Seafood	21.04.05	Shrimp/prawns
21.00.00	Fish/Seafood	21.04.06	Squid
21.00.00	Fish/Seafood	21.04.07	Other shellfish and non-fin fish
21.00.00	Fish/Seafood	21.04.08	Crab and crayfish
21.00.00	Fish/Seafood	21.05.01	Fish/seafood pie

21.00.00	Fish/Seafood	21.06.01	Fish/seafood casserole stirfry and fritters
21.00.00	Fish/Seafood	21.07.01	Fish/seafood products incl's fish fingers, fish cakes, fish paste and roe
22.00.00	Vegetables	22.01.01	Leafy greens includes lettuce, spinach, silver beet, bok choy etc.
22.00.00	Vegetables	22.02.01	Beans/peas/corn
22.00.00	Vegetables	22.03.01	Cooked or canned tomatoes
22.00.00	Vegetables	22.03.02	Purees and pastes
22.00.00	Vegetables	22.03.03	Raw
22.00.00	Vegetables	22.04.01	Carrots
22.00.00	Vegetables	22.04.02	Pumpkin/squash/butternut
22.00.00	Vegetables	22.04.03	Yams
22.00.00	Vegetables	22.05.01	Cauliflower/Broccoli/Brussel sprout/cabbage/turnip & other brassicas
22.00.00	Vegetables	22.06.01	Onion/garlic/leeks
22.00.00	Vegetables	22.07.01	Other vegetables includes parsnip, marrow/courgettes and eggplant etc.
22.00.00	Vegetables	22.08.01	Carrots/peas/beans/corn mixes
22.00.00	Vegetables	22.08.02	Stir-fry mixes
22.00.00	Vegetables	22.09.01	Mature legumes and pulses
22.00.00	Vegetables	22.09.02	Mature legumes and pulse products and dishes (includes baked beans)
22.00.00	Vegetables	22.09.03	Meat substitutes and dishes
22.00.00	Vegetables	22.10.01	Stuffed vegetables and vegetable dishes
22.00.00	Vegetables	22.11.01	Salad recipes (includes green salads, coleslaw, vegetable salads etc.)
23.00.00	Potatoes, kumara and taro	23.01.01	Potato (includes boiled and baked potatoes)
23.00.00	Potatoes, kumara and taro	23.02.01	Potato chips/wedges/croquette/hash browns
23.00.00	Potatoes, kumara and taro	23.03.01	Potato crisps - regular fat
23.00.00	Potatoes, kumara and taro	23.03.02	Potato crisps - reduced fat
23.00.00	Potatoes, kumara and taro	23.04.01	Mashed potatoes with cheese added
23.00.00	Potatoes, kumara and taro	23.04.02	Scalloped potatoes
23.00.00	Potatoes, kumara and taro	23.04.03	Stuffed potatoes and other potato dishes
23.00.00	Potatoes, kumara and taro	23.04.04	Potatoes with additions (e.g. mashed with fat/milk added)
23.00.00	Potatoes, kumara and taro	23.05.01	Kumara
23.00.00	Potatoes, kumara and taro	23.06.01	Taro
24.00.00	Snack foods	24.01.01	Corn snacks including corn chips
24.00.00	Snack foods	24.02.01	Pop corn
24.00.00	Snack foods	24.03.01	Extruded snacks and other crisps (not potato or corn)
24.00.00	Snack foods	24.04.01	Other including mixes
24.00.00	Snack foods	24.05.01	Other crisps e.g.. grain or kumara crisps
25.00.00	Fruit	25.01.01	Apple
25.00.00	Fruit	25.01.02	Pear
25.00.00	Fruit	25.01.03	Other pomme fruits
25.00.00	Fruit	25.02.01	Berry Fruit
25.00.00	Fruit	25.03.01	Stone fruit
25.00.00	Fruit	25.04.01	Oranges

25.00.00	Fruit	25.04.02	Other citrus fruits
25.00.00	Fruit	25.05.01	Banana
25.00.00	Fruit	25.05.02	Pineapple
25.00.00	Fruit	25.05.03	Other tropical fruits
25.00.00	Fruit	25.06.01	Other fruits
25.00.00	Fruit	25.07.01	Dried vine fruit
25.00.00	Fruit	25.07.02	Other dried fruit and mixes
25.00.00	Fruit	25.07.03	Fruit leather/roll ups
25.00.00	Fruit	25.08.01	Mixed fruits includes fruit salad
26.00.00	Nuts and Seeds	26.01.01	Peanuts
26.00.00	Nuts and Seeds	26.01.02	Coconut
26.00.00	Nuts and Seeds	26.01.03	Other nuts
26.00.00	Nuts and Seeds	26.02.01	Nut butters
26.00.00	Nuts and Seeds	26.02.02	Coconut products including coconut cream
26.00.00	Nuts and Seeds	26.02.03	Nut based dips
26.00.00	Nuts and Seeds	26.03.01	Seeds
26.00.00	Nuts and Seeds	26.04.01	Seed products
27.00.00	Sugar/sweets	27.01.01	Sugar
27.00.00	Sugar/sweets	27.02.01	Golden syrups
27.00.00	Sugar/sweets	27.02.02	Other sugar syrups including molasses, maple syrup, treacle
27.00.00	Sugar/sweets	27.03.01	Lollies
27.00.00	Sugar/sweets	27.03.02	Bubblegum and chewing gum
27.00.00	Sugar/sweets	27.04.01	Chocolate and chocolate based confectionery
27.00.00	Sugar/sweets	27.05.01	Sugar based toppings, sauces and icings
27.00.00	Sugar/sweets	27.06.01	Ice blocks including milk or juice base
27.00.00	Sugar/sweets	27.07.01	Jam/marmalade/honey
27.00.00	Sugar/sweets	27.08.01	Other e.g. jelly
27.00.00	Sugar/sweets	27.09.01	Artificial sweeteners
28.00.00	Soups and stocks	28.01.01	Soups containing meat
28.00.00	Soups and stocks	28.01.02	Soups containing chicken
28.00.00	Soups and stocks	28.01.03	Soups containing fish or seafood
28.00.00	Soups and stocks	28.01.04	Tomato based soups
28.00.00	Soups and stocks	28.01.05	Other vegetable soups
28.00.00	Soups and stocks	28.02.01	Stocks
29.00.00	Savoury sauces and condiments	29.01.01	Gravies
29.00.00	Savoury sauces and condiments	29.01.02	Other savoury sauces (no meat)
29.00.00	Savoury sauces and condiments	29.01.03	Tomato based pasta sauces (no meat)
29.00.00	Savoury sauces and condiments	29.01.04	Cream or oil based pasta sauces (no meat)
29.00.00	Savoury sauces and condiments	29.01.05	Pasta sauces containing meat/chicken/fish
29.00.00	Savoury sauces and condiments	29.02.01	Condiments, salt and other flavourings
29.00.00	Savoury sauces and condiments	29.03.01	Other additional sauces includes steak sauce, fruit sauces (eg plum/apricot/a

29.00.00	Savoury sauces and condiments	29.03.02	Tomato sauce
29.00.00	Savoury sauces and condiments	29.04.01	Roux sauces includes white sauces, cheese sauces
29.00.00	Savoury sauces and condiments	29.05.01	Mayonnaise and cream style dressings, full fat
29.00.00	Savoury sauces and condiments	29.05.02	Mayonnaise and cream style dressings, reduced fat
29.00.00	Savoury sauces and condiments	29.05.03	Oil and vinegar, French style dressings, full fat
29.00.00	Savoury sauces and condiments	29.05.04	Oil and vinegar, French style dressings, reduced fat
29.00.00	Savoury sauces and condiments	29.06.01	Pickles and chutneys
29.00.00	Savoury sauces and condiments	29.07.01	Yeast & vege extracts (e.g. marmite)
30.00.00	Non-alcoholic beverages	30.01.01	Tea includes black tea, herbal tea, green tea
30.00.00	Non-alcoholic beverages	30.02.01	Coffee
30.00.00	Non-alcoholic beverages	30.03.01	Hot drinks includes Milo, hot chocolate, cocoa and cereal beverages etc.
30.00.00	Non-alcoholic beverages	30.04.01	Fruit Juices includes apple, orange, grapefruit, grape etc.
30.00.00	Non-alcoholic beverages	30.05.01	Vegetable juices
30.00.00	Non-alcoholic beverages	30.06.01	Cordials and fruit drinks
30.00.00	Non-alcoholic beverages	30.07.01	Regular soft drinks,
30.00.00	Non-alcoholic beverages	30.07.02	Diet soft drinks
30.00.00	Non-alcoholic beverages	30.08.01	Water includes mineral and soda water, tap and filtered water
30.00.00	Non-alcoholic beverages	30.08.02	sweetened water
30.00.00	Non-alcoholic beverages	30.09.01	Sports drinks
30.00.00	Non-alcoholic beverages	30.10.01	Energy drinks
30.00.00	Non-alcoholic beverages	30.11.01	Powdered drinks
30.00.00	Non-alcoholic beverages	30.12.01	Other non-alcoholic beverages
31.00.00	Alcoholic beverages	31.01.01	Beer
31.00.00	Alcoholic beverages	31.02.01	Wine
31.00.00	Alcoholic beverages	31.03.01	Spirits
31.00.00	Alcoholic beverages	31.04.01	Liqueurs and cocktails
31.00.00	Alcoholic beverages	31.05.01	Other alcohol e.g. cider alcoholic soda
32.00.00	Dietary supplements	32.02.02	Dietary supplements1
32.00.00	Dietary supplements	32.03.01	Dietary supplements2
33.00.00	Snack bars	33.01.01	Fruit break/wholemeal fruit bars (fruit wrapped in cereal based casing)
33.00.00	Snack bars	33.02.01	Muesli bars (rolled oat base)
33.00.00	Snack bars	33.03.01	Soft and hard mixed grain bars (mixed cereal base)
33.00.00	Snack bars	33.04.01	Puffed cereal bars (based on rice or corn)
33.00.00	Snack bars	33.05.01	Other breakfast cereal based bars
33.00.00	Snack bars	33.06.01	Nuts and/or seed bars
99.99.99	Not applicable	99.99.99	Not applicable

Appendix B: SPEND Study price elasticity tables

Table 12: SPEND price elasticities used in aggregated food groups with Standard Errors

	Fruit	Vegetables	Beef, lamb & hogget	Poultry	Pork	Prepared, preserved & processed meat	Fish & seafood	Bread & breakfast cereals	Cakes & biscuits	Pastry cook products	Pasta & other cereal products	Milk, yoghurt & eggs	Cheese & cream	Butter	Margarine & edible oil	Sauces, sugar, condiments	Chocolate, confectionary, snacks	Ice cream	Other grocery food	Non-alcoholic beverages	Carbonated soft drinks	Ready to eat food	Energy drinks
**Fruit**	-0.58 (0.05)	0.06 (0.03)	-0.05 (0.04)	-0.01 (0.05)	0.04 (0.06)	-0.04 (0.03)	0.01 (0.02)	0.06 (0.04)	-0.17 (0.05)	0.04 (0.04)	-0.05 (0.02)	0.01 (0.03)	0.08 (0.02)	0.05 (0.05)	-0.04 (0.02)	0.04 (0.01)	0.01 (0.03)	0.00 (0.01)	-0.25 (0.04)	0.00 (0.01)	-0.11 (0.04)	-0.21 (0.04)	-0.09 (0.09)
**Vegetables**	0.05 (0.02)	-0.88 (0.03)	-0.08 (0.03)	-0.04 (0.03)	0.00 (0.05)	-0.02 (0.02)	0.03 (0.02)	-0.03 (0.02)	-0.10 (0.04)	-0.02 (0.03)	0.03 (0.01)	-0.03 (0.02)	0.01 (0.02)	-0.08 (0.03)	0.03 (0.02)	0.06 (0.01)	0.02 (0.02)	0.00 (0.01)	-0.17 (0.04)	0.03 (0.01)	-0.11 (0.03)	-0.04 (0.03)	0.03 (0.05)
**Beef, lamb & hogget**	-0.07 (0.04)	-0.11 (0.03)	-0.93 (0.06)	0.05 (0.05)	0.01 (0.06)	-0.01 (0.03)	-0.05 (0.03)	-0.11 (0.03)	-0.12 (0.04)	-0.08 (0.04)	-0.02 (0.02)	-0.03 (0.03)	-0.04 (0.02)	0.03 (0.05)	-0.02 (0.03)	-0.05 (0.02)	0.00 (0.03)	-0.02 (0.01)	-0.28 (0.05)	0.04 (0.01)	-0.03 (0.08)	0.05 (0.05)	0.15 (0.06)
**Poultry**	-0.19 (0.06)	-0.14 (0.05)	0.03 (0.07)	-1.70 (0.09)	0.21 (0.11)	0.11 (0.04)	-0.02 (0.04)	0.08 (0.06)	0.27 (0.07)	0.16 (0.06)	-0.03 (0.03)	0.17 (0.05)	0.02 (0.03)	0.25 (0.07)	-0.01 (0.04)	-0.01 (0.02)	-0.04 (0.05)	-0.04 (0.02)	-0.20 (0.07)	0.05 (0.02)	0.03 (0.08)	0.09 (0.06)	0.21 (0.11)
**Pork**	-0.13 (0.10)	-0.34 (0.11)	0.05 (0.12)	0.43 (0.14)	-4.51 (0.43)	0.25 (0.09)	-0.23 (0.09)	-0.01 (0.11)	0.35 (0.12)	-0.02 (0.11)	-0.07 (0.04)	0.20 (0.09)	0.03 (0.06)	0.17 (0.13)	0.15 (0.07)	-0.04 (0.04)	0.11 (0.09)	-0.06 (0.03)	0.04 (0.12)	0.12 (0.03)	0.65 (0.18)	0.11 (0.11)	1.95 (0.25)
**Prepared, preserved & processed meat**	-0.07 (0.03)	-0.07 (0.03)	-0.02 (0.05)	0.12 (0.04)	0.10 (0.05)	-1.05 (0.03)	0.00 (0.03)	-0.09 (0.03)	-0.06 (0.03)	-0.01 (0.04)	0.05 (0.02)	-0.07 (0.02)	0.01 (0.02)	0.11 (0.04)	-0.09 (0.03)	-0.06 (0.01)	-0.09 (0.03)	-0.06 (0.01)	-0.18 (0.04)	-0.01 (0.01)	-0.06 (0.04)	-0.10 (0.04)	0.07 (0.05)
**Fish & seafood**	-0.02 (0.03)	0.21 (0.04)	-0.16 (0.06)	-0.06 (0.05)	-0.11 (0.08)	0.12 (0.04)	-1.68 (0.07)	0.26 (0.04)	0.03 (0.03)	0.06 (0.04)	-0.09 (0.03)	0.24 (0.04)	0.18 (0.03)	-0.02 (0.03)	0.05 (0.02)	0.03 (0.02)	-0.11 (0.04)	-0.04 (0.02)	-0.22 (0.06)	0.03 (0.02)	-0.23 (0.05)	-0.05 (0.06)	-1.04 (0.17)
**Bread & breakfast cereals**	0.02 (0.04)	0.01 (0.03)	-0.12 (0.04)	0.05 (0.05)	0.08 (0.07)	-0.05 (0.02)	0.05 (0.02)	-0.73 (0.04)	0.06 (0.05)	-0.02 (0.04)	0.01 (0.02)	-0.15 (0.03)	0.00 (0.02)	0.02 (0.05)	-0.09 (0.02)	0.00 (0.01)	0.07 (0.03)	0.00 (0.01)	-0.14 (0.04)	0.02 (0.01)	0.06 (0.06)	-0.09 (0.03)	0.24 (0.08)
**Cakes & biscuits**	-0.15 (0.05)	-0.10 (0.05)	-0.06 (0.06)	0.07 (0.07)	0.29 (0.11)	0.00 (0.04)	0.04 (0.03)	-0.05 (0.06)	-0.97 (0.10)	-0.09 (0.05)	0.01 (0.02)	-0.04 (0.04)	-0.05 (0.03)	-0.08 (0.09)	-0.06 (0.04)	-0.01 (0.02)	0.03 (0.04)	-0.03 (0.01)	-0.10 (0.05)	-0.01 (0.01)	0.11 (0.11)	-0.06 (0.05)	0.24 (0.13)
**Pastry cook products**	-0.05 (0.06)	-0.11 (0.06)	-0.20 (0.09)	0.05 (0.09)	0.33 (0.12)	-0.12 (0.06)	-0.15 (0.05)	-0.47 (0.09)	0.43 (0.11)	-1.52 (0.18)	0.15 (0.04)	-0.40 (0.08)	-0.09 (0.05)	0.55 (0.14)	-0.26 (0.06)	-0.09 (0.03)	-0.19 (0.07)	-0.06 (0.02)	0.32 (0.11)	-0.08 (0.03)	-1.90 (0.28)	0.32 (0.08)	1.23 (0.22)
**Pasta & other cereal products**	-0.05 (0.04)	0.10 (0.04)	-0.16 (0.06)	0.28 (0.06)	-0.05 (0.07)	0.20 (0.04)	0.08 (0.05)	0.27 (0.06)	-0.07 (0.04)	0.12 (0.05)	-1.70 (0.04)	0.11 (0.04)	0.02 (0.03)	-0.02 (0.05)	0.06 (0.03)	-0.06 (0.03)	-0.12 (0.04)	0.00 (0.02)	0.10 (0.06)	0.07 (0.02)	-0.14 (0.05)	0.25 (0.09)	-0.36 (0.09)
**Milk, yoghurt & eggs**	-0.02 (0.03)	-0.03 (0.03)	-0.01 (0.04)	0.07 (0.04)	-0.01 (0.05)	-0.07 (0.02)	0.06 (0.02)	-0.14 (0.02)	-0.04 (0.05)	-0.01 (0.03)	-0.01 (0.01)	-0.86 (0.05)	-0.03 (0.02)	0.07 (0.07)	-0.01 (0.02)	-0.02 (0.01)	0.04 (0.03)	0.00 (0.01)	-0.17 (0.05)	0.05 (0.01)	-0.03 (0.04)	-0.04 (0.04)	0.09 (0.07)
**Cheese & cream**	0.18 (0.04)	0.04 (0.04)	-0.07 (0.05)	-0.01 (0.05)	0.03 (0.07)	0.12 (0.03)	0.06 (0.03)	0.11 (0.04)	-0.10 (0.04)	0.04 (0.04)	0.00 (0.02)	0.00 (0.03)	-1.04 (0.04)	0.28 (0.06)	-0.10 (0.03)	-0.04 (0.02)	-0.20 (0.04)	-0.02 (0.01)	-0.21 (0.05)	0.01 (0.01)	0.04 (0.05)	-0.22 (0.05)	-0.07 (0.06)
**Butter**	0.03 (0.14)	-0.50 (0.15)	-0.34 (0.16)	-0.40 (0.15)	0.39 (0.25)	0.13 (0.10)	0.06 (0.08)	0.09 (0.16)	-0.35 (0.20)	-0.56 (0.14)	-0.50 (0.07)	-0.03 (0.09)	0.01 (0.09)	-0.67 (0.28)	0.50 (0.07)	-0.57 (0.07)	-0.15 (0.12)	-0.09 (0.03)	-0.49 (0.16)	0.07 (0.03)	1.01 (0.32)	-0.05 (0.10)	0.29 (0.30)
**Margarine & edible oil**	-0.27 (0.06)	-0.05 (0.05)	0.02 (0.08)	0.15 (0.07)	0.12 (0.11)	0.00 (0.05)	0.04 (0.05)	-0.32 (0.06)	-0.27 (0.07)	0.03 (0.06)	-0.04 (0.03)	0.00 (0.05)	-0.07 (0.04)	0.43 (0.08)	-1.04 (0.07)	-0.08 (0.03)	-0.13 (0.05)	0.01 (0.02)	-0.16 (0.07)	0.06 (0.02)	-0.47 (0.10)	0.04 (0.06)	-0.62 (0.13)
**Sauces, sugar & condiments**	0.13 (0.03)	0.11 (0.03)	-0.18 (0.04)	0.15 (0.03)	-0.01 (0.05)	-0.01 (0.02)	0.05 (0.02)	-0.04 (0.02)	-0.15 (0.03)	0.05 (0.03)	-0.06 (0.02)	0.16 (0.03)	-0.11 (0.02)	0.10 (0.04)	0.05 (0.02)	-1.32 (0.02)	-0.21 (0.03)	-0.04 (0.01)	-0.12 (0.04)	0.03 (0.01)	0.01 (0.03)	0.06 (0.04)	-0.22 (0.05)
**Chocolate, confectionary & snacks**	0.12 (0.03)	0.02 (0.03)	-0.02 (0.04)	0.03 (0.04)	0.18 (0.06)	0.01 (0.02)	-0.01 (0.03)	0.21 (0.05)	0.04 (0.03)	-0.01 (0.03)	0.04 (0.02)	0.08 (0.03)	-0.08 (0.02)	0.12 (0.04)	-0.02 (0.02)	-0.05 (0.02)	-1.27 (0.04)	-0.08 (0.01)	-0.05 (0.05)	-0.08 (0.02)	0.31 (0.08)	0.07 (0.04)	0.27 (0.12)

**Ice cream**	0.19 (0.03)	0.19 (0.03)	-0.13 (0.04)	0.55 (0.07)	-0.01 (0.04)	-0.10 (0.03)	-0.05 (0.03)	0.11 (0.02)	0.22 (0.04)	-0.11 (0.03)	0.08 (0.02)	0.01 (0.02)	-0.03 (0.02)	-0.03 (0.02)	0.02 (0.01)	0.01 (0.01)	-0.09 (0.03)	-1.74 (0.06)	0.24 (0.06)	-0.06 (0.02)	0.20 (0.03)	0.38 (0.05)	-0.47 (0.07)
**Other grocery food**	-0.09 (0.02)	-0.12 (0.02)	-0.09 (0.04)	-0.08 (0.03)	0.01 (0.03)	-0.07 (0.02)	0.00 (0.02)	-0.10 (0.02)	-0.04 (0.02)	-0.04 (0.04)	0.03 (0.01)	-0.10 (0.02)	-0.01 (0.01)	-0.07 (0.03)	0.02 (0.02)	0.03 (0.01)	0.04 (0.02)	0.05 (0.01)	-0.38 (0.04)	0.03 (0.01)	0.07 (0.03)	-0.09 (0.04)	-0.12 (0.06)
**Non-alcoholic beverages**	-0.10 (0.03)	-0.04 (0.02)	0.00 (0.03)	0.05 (0.02)	0.11 (0.04)	0.02 (0.02)	0.04 (0.02)	0.03 (0.01)	-0.17 (0.04)	-0.03 (0.02)	-0.03 (0.02)	0.11 (0.02)	0.09 (0.02)	0.01 (0.02)	-0.02 (0.01)	-0.10 (0.02)	-0.12 (0.03)	-0.01 (0.01)	-0.13 (0.05)	-1.31 (0.04)	0.26 (0.06)	-0.25 (0.05)	-0.42 (0.08)
**Carbonated soft drinks**	-0.14 (0.08)	-0.27 (0.07)	0.23 (0.09)	0.59 (0.13)	0.06 (0.18)	0.17 (0.06)	-0.14 (0.05)	-0.21 (0.10)	0.69 (0.15)	-0.25 (0.09)	0.13 (0.03)	0.11 (0.07)	-0.02 (0.05)	0.67 (0.19)	-0.24 (0.06)	-0.02 (0.03)	-0.01 (0.07)	0.03 (0.02)	0.15 (0.08)	-0.18 (0.03)	-1.23 (0.28)	0.05 (0.08)	0.77 (0.34)
**Ready to eat food**	0.03 (0.03)	0.06 (0.02)	0.15 (0.03)	0.08 (0.02)	0.13 (0.03)	0.03 (0.02)	-0.01 (0.02)	0.11 (0.03)	0.08 (0.03)	0.12 (0.02)	0.10 (0.02)	-0.06 (0.02)	0.03 (0.02)	0.02 (0.01)	-0.01 (0.01)	0.01 (0.01)	0.00 (0.02)	0.05 (0.01)	0.04 (0.03)	-0.08 (0.01)	0.10 (0.03)	-0.93 (0.04)	0.15 (0.05)
**Energy drinks**	-1.14 (0.19)	0.39 (0.16)	0.36 (0.21)	0.18 (0.24)	1.78 (0.42)	-0.08 (0.13)	-0.23 (0.11)	0.32 (0.20)	3.18 (0.29)	-0.06 (0.25)	0.19 (0.08)	0.25 (0.17)	-0.40 (0.10)	0.49 (0.26)	-0.25 (0.11)	-0.35 (0.08)	-0.58 (0.13)	-0.07 (0.06)	0.31 (0.24)	-0.71 (0.09)	2.73 (0.36)	0.10 (0.22)	-0.31 (0.60)

Table 13: Selected cross-PEs for AS1 sensitivity analysis: Suppressed those SPEND cross-PEs that we classified as 'weak', i.e. where the BODE[3] |cross-PE| ≤ 0.04

	Fruit	Vegetables	Beef, lamb & hogget	Poultry	Pork	Prepared, preserved & processed meat	Fish & seafood	Bread & breakfast cereals	Cakes & biscuits	Pastry cook products	Pasta & other cereal products	Milk, yoghurt & eggs	Cheese & cream	Butter	Margarine & edible oil	Sauces, sugar, condiments	Chocolate, confectionary, snacks	Ice cream	Other grocery food	Non-alcoholic beverages	Carbonated soft drinks	Ready to eat food	Energy drinks
**Fruit**	-0.58	0.06							-0.17	0.04		0.01	0.08		-0.04		0.01		-0.25	0		-0.21	-0.09
**Vegetables**	0.05	-0.88							-0.1	-0.02						0.06	0.02		-0.17	0.03	-0.11	-0.04	0.03
**Beef, lamb & hogget**			-0.93	0.05	0.01	-0.01	-0.05			-0.08		-0.03			-0.02	-0.05	0	-0.02	-0.28	0.04	-0.03	0.05	0.15
**Poultry**			0.03	-1.7		0.11				0.16					-0.01	-0.01	-0.04	-0.04	-0.2	0.05	0.03	0.09	0.21
**Pork**			0.05		-4.51	0.25				-0.02						-0.04	0.11		0.04	0.12	0.65	0.11	1.95
**Prepared, preserved & processed meat**			-0.02	0.12	0.1	-1.05			-0.06	-0.01			0.01			-0.06	-0.09		-0.18			-0.1	0.07
**Fish & seafood**			-0.16				-1.68	0.26	0.03	0.06	-0.09			-0.02	0.05		-0.11	-0.04	-0.22	0.03		-0.05	
**Bread & breakfast cereals**	0.02				0.08			-0.73	0.06	-0.02				0.02	-0.09	0	0.07	0	-0.14	0.02	0.06	-0.09	0.24
**Cakes & biscuits**	-0.15					0	0.04	-0.05	-0.97	-0.09	0.01	-0.04	-0.05	-0.08	-0.06	-0.01	0.03	-0.03	-0.1	-0.01	0.11	-0.06	0.24
**Pastry cook products**	-0.05	-0.11	-0.2	0.05	0.33	-0.12	-0.15	-0.47	0.43	-1.52	0.15	-0.4	-0.09	0.55	-0.26	-0.09	-0.19	-0.06	0.32	-0.08	-1.9	0.32	1.23
**Pasta & other cereal products**	-0.05	0.1	-0.16	0.28	-0.05				-0.07	0.12	-1.7	0.11	0.02	-0.02	0.06	-0.06	-0.12		0.1	0.07	-0.14	0.25	-0.36
**Milk, yoghurt & eggs**	-0.02		-0.01						-0.04	-0.01		-0.86	-0.03		-0.01	-0.02	0.04	0	-0.17	0.05		-0.04	0.09
**Cheese & cream**	0.18					0.12		0.11	-0.1	0.04		0	-1.04		-0.1	-0.04	-0.2	-0.02	-0.21		0.04	-0.22	
**Butter**		-0.5			0.39		0.06	0.09	-0.35	-0.56	-0.5			-0.67	0.5	-0.57	-0.15	-0.09	-0.49		1.01	-0.05	0.29
**Margarine & edible oil**	-0.27				0.12		0.04		-0.27	0.03		0	-0.07		-1.04		-0.13		-0.16			0.04	
**Sauces, sugar & condiments**									-0.15	0.05			-0.11			-1.32	-0.21	-0.04	-0.12		0.01	0.06	-0.22
**Chocolate, confectionary & snacks**	0.12	0.02	-0.02	0.03	0.18	0.01	-0.01	0.21	0.04	-0.01	0.04	0.08	-0.08	0.12	-0.02	-0.05	-1.27	-0.08	-0.05	-0.08	0.31	0.07	
**Ice cream**				0.55			-0.05	0.11	0.22	-0.11		0.01	-0.03	-0.03		0.01	-0.09	-1.74	0.24	-0.06	0.2	0.38	-0.47

**Other grocery food**	-0.09					-0.07	0		-0.04	-0.04		-0.1	-0.01		0.02			0.05	-0.38			-0.09	
**Non-alcoholic beverages**	-0.1	-0.04	0	0.05	0.11		0.04	0.03	-0.17	-0.03	-0.03	0.11		0.01		-0.1	-0.12	-0.01	-0.13	-1.31	0.26	-0.25	-0.42
**Carbonated soft drinks**		-0.27						-0.21	0.69	-0.25	0.13	0.11	-0.02				-0.01	0.03	0.15	-0.18	-1.23	0.05	0.77
**Ready to eat food**	0.03	0.06	0.15	0.08	0.13	0.03	-0.01	0.11	0.08	0.12	0.1	-0.06	0.03	0.02	-0.01	0.01	0	0.05	0.04	-0.08	0.1	-0.93	0.15
**Energy drinks**	-1.14	0.39	0.36	0.18	1.78	-0.08		0.32	3.18	-0.06	0.19	0.25		0.49		-0.35		-0.07	0.31	-0.71	2.73	0.1	-0.31

Table 14: Selected cross-PEs for AS2 sensitivity analysis: Suppressed those SPEND cross-PEs that we classified as 'weak' or 'moderate', i.e. where the BODE[3] |cross-PE| ≤ 0.09

	Fruit	Vegetables	Beef, lamb & hogget	Poultry	Pork	Prepared, preserved & processed meat	Fish & seafood	Bread & breakfast cereals	Cakes & biscuits	Pastry cook products	Pasta & other cereal products	Milk, yoghurt & eggs	Cheese & cream	Butter	Margarine & edible oil	Sauces, sugar, condiments	Chocolate, confectionary, snacks	Ice cream	Other grocery food	Non-alcoholic beverages	Carbonated soft drinks	Ready to eat food	Energy drinks
**Fruit**	-0.58								-0.17	0.04					-0.04							-0.21	-0.09
**Vegetables**		-0.88								-0.02											-0.11	-0.04	
**Beef, lamb & hogget**			-0.93	0.05	0.01	-0.01				-0.08												0.05	
**Poultry**				-1.7						0.16												0.09	
**Pork**			0.05		-4.51					-0.02												0.11	
**Prepared, preserved & processed meat**						-1.05				-0.01												-0.1	0.07
**Fish & seafood**							-1.68		0.03	0.06												-0.05	
**Bread & breakfast cereals**								-0.73	0.06	-0.02							0.07		-0.14	0.02	0.06	-0.09	0.24
**Cakes & biscuits**	-0.15								-0.97	-0.09		-0.04		-0.08	-0.06		0.03					-0.06	
**Pastry cook products**									0.43	-1.52		-0.4	-0.09	0.55	-0.26	-0.09	-0.19	-0.06	0.32	-0.08	-1.9	0.32	1.23
**Pasta & other cereal products**									-0.07	0.12	-1.7										-0.14	0.25	
**Milk, yoghurt & eggs**									-0.04	-0.01		-0.86	-0.03					0				-0.04	0.09
**Cheese & cream**									-0.1	0.04			-1.04					-0.02	-0.21			-0.22	
**Butter**									-0.35	-0.56				-0.67	0.5		-0.15					-0.05	0.29
**Margarine & edible oil**									-0.27	0.03					-1.04				-0.16			0.04	
**Sauces, sugar & condiments**										0.05						-1.32	-0.21		-0.12			0.06	-0.22
**Chocolate, confectionary & snacks**								0.21	0.04	-0.01	0.04				-0.02	-0.05	-1.27	-0.08	-0.05			0.07	
**Ice cream**										-0.11		0.01	-0.03			0.01	-0.09	-1.74	0.24	-0.06		0.38	-0.47
**Other grocery food**									-0.04	-0.04		-0.1	-0.01		0.02			0.05	-0.38			-0.09	

**Non-alcoholic beverages**									-0.17	-0.03						-0.1		-0.01	-0.13	-1.31	0.26	-0.25	-0.42
**Carbonated soft drinks**										-0.25	0.13								0.15	-0.18	-1.23	0.05	
**Ready to eat food**									0.08	0.12	0.1	-0.06	0.03	0.02	-0.01	0.01	0	0.05	0.04	-0.08	0.1	-0.93	0.15
**Energy drinks**						-0.08				-0.06		0.25				-0.35		-0.07	0.31	-0.71	2.73	0.1	-0.31

Table 15: Selected cross-PEs for BS1 sensitivity analysis: Retain SPEND own-PEs, but using the median BODE[3] cross-PEs from the literature.

	Fruit	Vegetables	Beef, lamb & hogget	Poultry	Pork	Prepared, preserved & processed meat	Fish & seafood	Bread & breakfast cereals	Cakes & biscuits	Pastry cook products	Pasta & other cereal products	Milk, yoghurt & eggs	Cheese & cream	Butter	Margarine & edible oil	Sauces, sugar, condiments	Chocolate, confectionary, snacks	Ice cream	Other grocery food	Non-alcoholic beverages	Carbonated soft drinks	Ready to eat food	Energy drinks
**Fruit**	-0.58								-0.17								0.01	0.00					
**Vegetables**		-0.88	-0.08	-0.04	0.00	-0.02	0.03				0.03												
**Beef, lamb & hogget**		-0.11	-0.93	0.05	0.01	-0.01	-0.05			-0.08	-0.02					-0.05							
**Poultry**		-0.14	0.03	-1.70	0.21	0.11	-0.02			0.16	-0.03					-0.01							
**Pork**		-0.34	0.05	0.43	-4.51	0.25	-0.23			-0.02	-0.07					-0.04							
**Prepared, preserved & processed meat**		-0.07	-0.02	0.12	0.10	-1.05		-0.09		-0.01						-0.06							
**Fish & seafood**		0.21	-0.16	-0.06	-0.11		-1.68									0.03							
**Bread & breakfast cereals**						-0.05		-0.73				-0.15	0.00	0.02	-0.09	0.00							
**Cakes & biscuits**	-0.15								-0.97				-0.05	-0.08			0.03						
**Pastry cook products**			-0.20	0.05	0.33	-0.12				-1.52						-0.09						0.32	
**Pasta & other cereal products**		0.10	-0.16	0.28	-0.05					0.12	-1.70		0.02			-0.06							
**Milk, yoghurt & eggs**								-0.14				-0.86											
**Cheese & cream**								0.11	-0.10		0.00		-1.04										
**Butter**								0.09	-0.35					-0.67	0.50	-0.57							
**Margarine & edible oil**								-0.32						0.43	-1.04								
**Sauces, sugar & condiments**			-0.18	0.15	-0.01	-0.01	0.05	-0.04		0.05	-0.06			0.10		-1.32							
**Chocolate, confectionary & snacks**	0.12								0.04								-1.27	-0.08		-0.08	0.31		0.27
**Ice cream**	0.19																-0.09	-1.74					

**Other grocery food**																			-0.38				
**Non-alcoholic beverages**																	-0.12			-1.31	0.26		-0.42
**Carbonated soft drinks**																	-0.01			-0.18	-1.23		0.77
**Ready to eat food**										0.12												-0.93	
**Energy drinks**																	-0.58			-0.71	2.73		-0.31

Table 16: Selected cross-PEs for BS2 sensitivity analysis: Suppressed those BODE^3 cross-PEs that we classified as 'weak', i.e. where the BODE^3 |cross-PE| ≤ 0.04

	Fruit	Vegetables	Beef, lamb & hogget	Poultry	Pork	Prepared, preserved & processed meat	Fish & seafood	Bread & breakfast cereals	Cakes & biscuits	Pastry cook products	Pasta & other cereal products	Milk, yoghurt & eggs	Cheese & cream	Butter	Margarine & edible oil	Sauces, sugar, condiments	Chocolate, confectionary, snacks	Ice cream	Other grocery food	Non-alcoholic beverages	Carbonated soft drinks	Ready to eat food	Energy drinks
**Fruit**	-0.58	-0.09							0.37			-0.09	-0.08		-0.17		0.06		0.15	0.10			0.20
**Vegetables**	-0.10	-0.88							0.13							-0.05	0.06		-0.09	0.09	0.18		0.10
**Beef, lamb & hogget**			-0.93	0.30	0.30	0.22	0.04					0.09			0.04	0.05	0.08	-0.07	-0.08	0.12	0.10		-0.08
**Poultry**			0.13	-1.70		-0.05									0.10	0.05	0.05	-0.07	-0.08	0.04	0.10		-0.08
**Pork**			0.26		-4.51	0.07										0.08	0.08		-0.08	0.07	0.10		-0.08
**Prepared, preserved & processed meat**			0.05	-0.06	0.15	-1.05			-0.13				0.10			0.05	0.08		-0.08				-0.17
**Fish & seafood**			0.04				-1.68	0.08			0.05			-0.10	-0.05		0.05	-0.06					
**Bread & breakfast cereals**	-0.06				-0.07			-0.73	-0.36					0.05	-0.05	-0.05	0.47	-0.09	-0.19	0.24	0.18		-0.29
**Cakes & biscuits**	0.28					-0.07		-0.11	-0.97		-0.13	-0.29	-0.14	-0.17		0.15	0.31	-0.11	-0.04	-0.10	-0.05		0.04
**Pastry cook products**										-1.52													
**Pasta & other cereal products**	-0.06	0.08	-0.04	-0.04	-0.04				-0.34		-1.70	-0.07	0.05	-0.12	-0.08	0.05	-0.14		0.14	-0.10	-0.20		-0.07
**Milk, yoghurt & eggs**	-0.14		0.13						-0.42			-0.86	0.34		-0.13	-0.07	0.09	0.27	-0.08	0.05			-0.44
**Cheese & cream**	-0.09					0.07		0.05	-0.21			0.12	-1.04		0.04	0.06	0.09	0.19	-0.62		0.09		

**Butter**		0.06			-0.04		-0.13	0.07	-0.78		-0.07			-0.67	0.19	0.04	0.22	-0.11	- 0.10		-0.10		-0.19
**Margarine & edible oil**	-0.09				0.07		-0.10					-0.07	0.09		-1.04		0.04						
**Sauces, sugar & condiments**									0.04				0.12			-1.32	0.81	0.10	0.81		-0.08		0.29
**Chocolate, confectionary & snacks**	0.12	0.12	0.07	0.07	0.07	0.07		0.27	0.32		-0.29	0.08	0.12	0.09		0.25	-1.27	-0.27	- 0.28	-0.07	0.15		
**Ice cream**				-0.05			-0.04	-0.09	-0.13			0.22	0.28	-0.09		0.16	-0.18	-1.74	- 0.36	-0.18	-0.12		-0.46
**Other grocery food**	-0.07					0.09			-0.17			-0.46	-0.46					-0.46	- 0.38				
**Non-alcoholic beverages**	0.06	0.15	0.06	0.05	0.07		-0.05	0.13	-0.17		-0.12	0.07		-0.05		0.21	-0.13	-0.20	0.46	-1.31	0.35		-0.66
**Carbonated soft drinks**		0.14						0.07	-0.04		-0.22		0.07				0.12	-0.07	0.29	0.17	-1.23		0.15
**Ready to eat food**																						-0.93	
**Energy drinks**	0.13	0.13	-0.14	-0.14	-0.14	-0.17		-0.12	0.06		-0.08	-0.40		-0.06		0.45		-0.43	0.73	-0.56	0.26		-0.31

Table 17: Selected cross-PEs for BS3 sensitivity analysis: Suppressed those BODE³ cross-PEs that we classified as 'weak' or 'moderate', i.e. where the BODE³ |cross-PE| ≤ 0.09

	Fruit	Vegetables	Beef, lamb & hogget	Poultry	Pork	Prepared, preserved & processed meat	Fish & seafood	Bread & breakfast cereals	Cakes & biscuits	Pastry cook products	Pasta & other cereal products	Milk, yoghurt & eggs	Cheese & cream	Butter	Margarine & edible oil	Sauces, sugar, condiments	Chocolate, confectionary, snacks	Ice cream	Other grocery food	Non-alcoholic beverages	Carbonated soft drinks	Ready to eat food	Energy drinks
**Fruit**	-0.58								0.37						-0.17								0.20
**Vegetables**		-0.88																			0.18		
**Beef, lamb & hogget**			-0.93	0.30	0.30	0.22																	
**Poultry**				-1.70																			
**Pork**			0.26		-4.51																		
**Prepared, preserved & processed meat**						-1.05																	-0.17
**Fish & seafood**							-1.68																
**Bread & breakfast cereals**								-0.73	-0.36								0.47		- 0.19	0.24	0.18		-0.29
**Cakes & biscuits**	0.28								-0.97			-0.29		-0.17			0.31						
**Pastry cook products**										-1.52													
**Pasta & other cereal products**									-0.34		-1.70										-0.20		
**Milk, yoghurt & eggs**									-0.42			-0.86	0.34					0.27					-0.44
**Cheese & cream**									-0.21				-1.04					0.19	- 0.62				
**Butter**									-0.78					-0.67	0.19		0.22						-0.19
**Margarine & edible oil**															-1.04								
**Sauces, sugar & condiments**													0.12			-1.32	0.81		0.81				0.29

**Chocolate, confectionary & snacks**								0.27	0.32		-0.29					0.25	-1.27	-0.27	-0.28				
**Ice cream**												0.22	0.28			0.16	-0.18	-1.74	-0.36	-0.18			-0.46
**Other grocery food**									-0.17			-0.46	-0.46					-0.46	-0.38				
**Non-alcoholic beverages**									-0.17							0.21		-0.20	0.46	-1.31	0.35		-0.66
**Carbonated soft drinks**											-0.22								0.29	0.17	-1.23		
**Ready to eat food**																						-0.93	
**Energy drinks**						-0.17						-0.40				0.45		-0.43	0.73	-0.56	0.26		-0.31

Table 18: Selected cross-PEs for BS4 sensitivity analysis: Use the median BODE[3] own and cross-PEs from the literature

	Fruit	Vegetables	Beef, lamb & hogget	Poultry	Pork	Prepared, preserved & processed meat	Fish & seafood	Bread & breakfast cereals	Cakes & biscuits	Pastry cook products	Pasta & other cereal products	Milk, yoghurt & eggs	Cheese & cream	Butter	Margarine & edible oil	Sauces, sugar, condiments	Chocolate, confectionary, snacks	Ice cream	Other grocery food	Non-alcoholic beverages	Carbonated soft drinks	Ready to eat food	Energy drinks
**Fruit**	-0.71	-0.09	0.00	0.00	-0.02	0.00	0.01	-0.03	0.37		-0.01	-0.09	-0.08	-0.04	-0.17	-0.02	0.06	-0.03	0.15	0.10	0.00		0.20
**Vegetables**	-0.10	-0.56	0.01	0.01	0.00	0.01	0.00	0.00	0.13		0.00	-0.02	-0.03	0.03	-0.04	-0.05	0.06	-0.02	-0.09	0.09	0.18		0.10
**Beef, lamb & hogget**	-0.02	0.00	-0.72	0.30	0.30	0.22	0.04	0.00	0.00		-0.03	0.09	-0.02	0.03	0.04	0.05	0.08	-0.07	-0.08	0.12	0.10		-0.08
**Poultry**	-0.02	-0.01	0.13	-0.66	0.03	-0.05	0.02	0.00	0.00		-0.03	0.02	-0.02	0.03	0.10	0.05	0.05	-0.07	-0.08	0.04	0.10		-0.08
**Pork**	-0.02	-0.01	0.26	0.00	-0.65	0.07	0.02	0.00	0.00		-0.03	0.00	-0.01	0.00	0.00	0.08	0.08	-0.03	-0.08	0.07	0.10		-0.08
**Prepared, preserved & processed meat**	-0.02	-0.01	0.05	-0.06	0.15	-0.62	-0.02	0.03	-0.13		0.00	0.00	0.10	0.01	-0.02	0.05	0.08	-0.03	-0.08	0.01	0.01		-0.17
**Fish & seafood**	0.00	-0.01	0.04	0.02	0.02	0.00	-0.61	0.08			0.05	-0.03	-0.01	-0.10	-0.05	0.01	0.05	-0.06		-0.03	0.00		0.00
**Bread & breakfast cereals**	-0.06	-0.02	-0.01	-0.01	-0.07	0.02	0.01	-0.52	-0.36		0.00	-0.01	0.01	0.05	-0.05	-0.05	0.47	-0.09	-0.19	0.24	0.18		-0.29
**Cakes & biscuits**	0.28	0.00	-0.01	-0.01	-0.01	-0.07		-0.11	-0.61		-0.13	-0.29	-0.14	-0.17		0.15	0.31	-0.11	-0.04	-0.10	-0.05		0.04
**Pastry cook products**																							
**Pasta & other cereal products**	-0.06	0.08	-0.04	-0.04	-0.04	-0.02	0.01	-0.04	-0.34		-0.60	-0.07	0.05	-0.12	-0.08	0.05	-0.14	0.03	0.14	-0.10	-0.20		-0.07
**Milk, yoghurt & eggs**	-0.14	-0.03	0.13	0.03	-0.01	0.03	0.00	0.00	-0.42		0.00	-0.59	0.34	-0.04	-0.13	-0.07	0.09	0.27	-0.08	0.05	0.00		-0.44
**Cheese & cream**	-0.09	-0.03	0.00	-0.01	0.00	0.07	0.03	0.05	-0.21		0.02	0.12	-0.50	0.02	0.04	0.06	0.09	0.19	-0.62	-0.02	0.09		0.00
**Butter**	-0.02	0.06	0.01	0.01	-0.04	0.00	-0.13	0.07	-0.78		-0.07	-0.02	-0.01	-0.56	0.19	0.04	0.22	-0.11	-0.10	-0.03	-0.10		-0.19
**Margarine & edible oil**	-0.09	-0.02	0.01	0.01	0.07	0.00	-0.10	0.01			-0.03	-0.07	0.09	0.01	-0.54	0.03	0.04	-0.01		0.00	0.02		0.02
**Sauces, sugar & condiments**	-0.01	0.04	0.01	0.01	0.02	0.00	0.01	0.01	0.04		-0.03	-0.01	0.12	0.01	0.01	-0.56	0.81	0.10	0.81	0.01	-0.08		0.29
**Chocolate, confectionary & snacks**	0.12	0.12	0.07	0.07	0.07	0.07		0.27	0.32		-0.29	0.08	0.12	0.09		0.25	-0.74	-0.27	-0.28	-0.07	0.15		0.00

**Ice cream**	-0.03	-0.02	-0.01	-0.05	-0.01	0.00	-0.04	-0.09	-0.13		0.02	0.22	0.28	-0.09	0.02	0.16	-0.18	-0.60	-0.36	-0.18	-0.12		-0.46
**Other grocery food**	-0.07	0.00	0.00	0.00	0.00	0.09		0.00	-0.17		0.00	-0.46	-0.46	0.00		0.00	0.00	-0.46	-0.87	0.00	0.00		0.00
**Non-alcoholic beverages**	0.06	0.15	0.06	0.05	0.07	0.00	-0.05	0.13	-0.17		-0.12	0.07	-0.02	-0.05	0.00	0.21	-0.13	-0.20	0.46	-0.80	0.35		-0.66
**Carbonated soft drinks**	0.01	0.14	0.04	0.04	0.04	0.00	0.00	0.07	-0.04		-0.22	0.01	0.07	-0.01	0.02	0.01	0.12	-0.07	0.29	0.17	-0.58		0.15
**Ready to eat food**																							
**Energy drinks**	0.13	0.13	-0.14	-0.14	-0.14	-0.17	0.00	-0.12	0.06		-0.08	-0.40	0.00	-0.06	0.02	0.45	0.00	-0.43	0.73	-0.56	0.26		-0.58

Appendix C: DISMOD II example for lung cancer

NZBDS Code	NZBDS Description	Finalised ICD10 Codes	Source of Prevalence (or Incidence) Data	Number of health states
C09	Lung	C33-C34	New Zealand Cancer Registry and NMDS (MORT)	4

1. Step 1: Data compilation and pre-processing of parameters:
    a. Incidence in 2011:
        i. Using lung cancer incidence regression model outputs for the year 2011 previously generated by BODE[3 46], we first weighted estimates by the New Zealand population within each area-level deprivation tertile for each age (5-year age groups from 25 years to 84 years), sex, and ethnic group (Māori and Non-Māori).
        ii. Then, to estimate incidence rates for the older ages, we used a simple linear extrapolation of rates for 85-89, 90-94 and 95-99 by using the trends in cancer registry data for 2008, 2009 and 2010.

http://www.health.govt.nz/publication/cancer-new-registrations-and-deaths-2010
http://www.health.govt.nz/publication/cancer-new-registrations-and-deaths-2009
http://www.health.govt.nz/publication/cancer-new-registrations-and-deaths-2008

1. Specifically, we calculated the rate ratio (pooled by sex and ethnicity) of the 85+ group over the rate for 80-84 year olds. Using this rate ratio, we generated rates for 5-year age groups to 99+ years using a linear extrapolation (see example
2. **Figure 15**; Excel spreadsheets available on request).

Figure 15: Example of linear extrapolation of incidence of lung cancer for older age groups

Maori Female							
Age group	Age	Rate	Person years (pop)		Age*PY	Estimates	Logic check
75-79	77.5	0.006432	3100			0.006432	
80-84	82.5	0.005873	1700			0.005873	
85-89	87.5		700		61250	0.005087	3.560812
90-94	92.5		225		20812.5	0.004301	0.967723
95-99	97.5		75		7312.5	0.003515	0.263633
					89375		4.792169
	77.5						
	82.5	0.005873					
85+	89.375	0.004792	1000			0.004792	
RR 85+ to 80-84 from NZCR			Slope	-0.00015718		Rate	0.004792
	0.816			↓		Check = 0	0
Purpose							
	To extrapolate rates to older ages, assuming log-linear increase in rates						

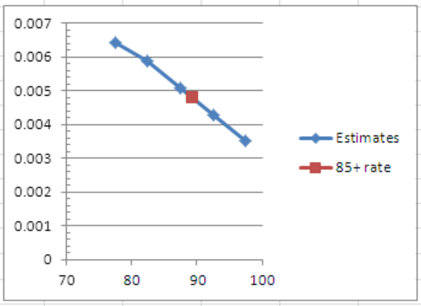

b. Prevalence

i. We compiled NZBDS data on prevalence from the year 2006 for 5-year age groups to 85+ years, with a five-year look back period. We opted to keep prevalence rates the same as 2006 rates, as 2011 prevalence is a function of all of changing population counts, changing incidence and changing survival. (NB: It would be possible to estimate 2011 prevalence using a similar equation to that specified in the main report for pYLDs, but prevalence itself is 'just' one input to DISMOD II whereas pYLDs are more directly inputted to the multi-state life-table model through the disability rates.) Due to this assumption, we down-weighted the importance of this parameter in DISMOD II (see example, Figure 16, page 90).

ii. To calculate a *starting* estimate of 5-year prevalence rates for ages over 84 years, we assumed that [incidence] × [duration].

-

c. Mortality

i. We compiled death counts by age, sex and ethnicity from the NZBDS, for the years 2005-2007.

ii. In order to update these counts to the year 2011, we used Cancer Mortality Projections from the Ministry of Health (Table 1 of ref [73]).

1. This document includes 10-year projections, so we manipulated these to get an annual percentage change.
2. Thus for males, the annual percentage change = -ln(1-0.26) = -2.74%
3. The formula for the rate in 2011 (5 years of change) = 2006mortrate × exp(annualpercentchange×5)

iii. Then, to generate estimates for 5-year age groups over 84 years, we used a linear extrapolation.

-

d. Case-fatality rate, Remission rate and Duration (time in years):

i. We compiled average background mortality rates (BMR) by age, sex and ethnicity using the Statistics New Zealand life-tables for 2010-2012.

ii. We compiled Relative Survival Rates (RSRs) for lung cancer at month 60 (5-years) from internal BODE[3] cancer excess mortality rate report. [74]

iii. Together with our estimates for incidence, mortality and prevalence, these *starting* parameters were generated, using the following formulas:

1. Crude duration = Prevalence/incidence
2. Case-fatality rate + Remission = (1/Duration) – BMR
3. Case-fatality rate = CFR + Remission *(1-RSR)
4. Remission = CFR + Remission * RSR

*To emphasize, these are just starting parameters to input to DISMOD II, not the parameters used in the MSLT model.

2. Step 2: Processing in DISMOD II software

Below are examples of the weighting schemes used for lung cancer parameters (Figure 16, page 90).

Figure 16: Example of parameter weighting in DISMOD II

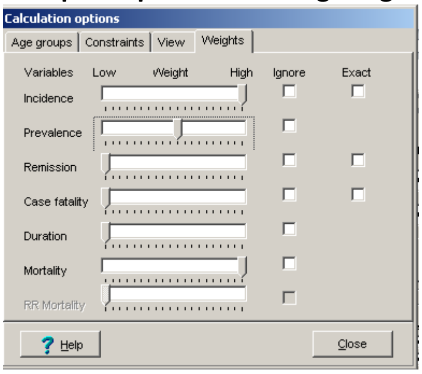

Note that there was sometimes considerable instability in the case-fatality rates at younger ages. This is a function of sparse data, and the case-fatality rate needing to 'move' to reconcile with the incidence and mortality inputs (and to a lesser extent prevalence). Once inputted to the BODE[3] DIET MSLT model, it does however balance out to ensure a target mortality rate (which largely drives the health loss/gain).

Appendix D: Parameterisation of 'DM as both a risk factor and disease'

Section 4.01. Baseline parametrization

4.01.1. Diabetes inputs to DISMOD: incidence, prevalence, mortality

We used VDR data, linked to New Zealand mortality data. The VDR is a register of all people (i.e. a list of unique personal identifiers) estimated to have diabetes on the 31 December for each year (1999 to 2015). The 'ascription' of DM is based on an algorithm of number of HbA1c tests, blood tests and medications. This includes all publicly funded hospitalization discharges between 1999 and 2015 that contain any diagnosis code of diabetes mellitus; events that contain specialist clinic (endocrinology) codes, diabetes education management codes, and diabetes fundus screening codes from National Non-Admitted Patient Collection and Personal Health Non Case Weight, between 2002 and 2015; individuals with two or more dispensings between 2014 and 2015 with certain chemical IDs from pharmaceutical data; laboratory claims with a visit date between 2014 and 2015 with four or more HbA1c lab tests and also having an ACR test (Microalbumin, early morning urine) in this period. This dataset excludes patients that only received diabetes education or attended specialist clinics, and women aged 12-45 (at dispensing) that only received metformin hydrochloride. However, it should be noted that we only have access to the VDR data from 2005 to 2014.

It must be noted that the VDR is getting 'better' or more comprehensive over time, meaning that if used to generate year-on-year incidence rates it will be spuriously high. (This may become less of a problem in future years once data systems and case definitions equilibrate.) However, it should be more accurate for prevalence, and if prevalence cases are also used to generate morbidity and costings, and mortality rates among this pool of prevalent cases, then there is coherence for these parameters excluding incidence.

(i) Incidence and prevalence rates

In principle:

- The DM prevalence is just that observed on 31 December 2011
- The DM incidence is the new cases observed each year. But note above, we expect it will be spuriously high using VDR data up to 2014 at least. The decision was therefore made to ignore incidence in DISMOD.

Regression on the VDR linked with mortality and core population files were used to estimate annual prevalence (logistic model; main effects of sex, age (categorical in five year age groups), and ethnicity; and interactions of main effects), using the predicted values for 2011.

Due to the artificially high estimates of incidence this parameter was ignored in DISMOD; details are provided below.

(ii) Mortality rates

Diabetes is a difficult disease to model due to itself being a risk factor for other diseases, and therefore having mortality rates dependent with other diseases (e.g. it is no longer viable to assume

independence of disease incidence and mortality when consider DM and CHD). It is important to keep in mind the BODE[3] DIET MSLT that is being parameterized, and its model structure. Namely:

- DM is treated as a disease state just as any of the other states are (e.g. CHD, stroke, lung cancer). However, it is also a risk factor in and of itself for CHD and stroke, meaning that changes in DM prevalence are linked through PIFs to changes in CHD and stroke incidence.
- A diagnosis of DM causes a non-ignorable increase in mortality for deaths coded with other than DM as the underlying cause of death. Some of this is causally due to DM, but some of it is due to confounding or correlated common causes (e.g. BMI as a risk factor for both DM and a range of cancer deaths and stroke and CHD).
    - For the purposes of the DIET MSLT, we classify CHD and stroke as causally related. We assume this is captured by the above link of changing DM prevalence to changing CHD and stroke incidence (through a PIF) that then flows onto change in mortality from CHD and stroke, per se.
    - DM-coded deaths – by definition – are causally due to DM. Changes in such DM-specific or DM-coded mortality in the DM state (due to changes in disease incidence from a given intervention) link to the main life-table, capturing mortality rate gains from interventions lowering DM incidence.
    - The non-causally related deaths (i.e. non-CHD, non-stroke and non-DM-coded, or simply 'other') are not captured as an effect of the intervention, and therefore do not link through to the main lifetable. However, they still matter as far as determining the prevalence. That is, if we do not allow for higher 'other' competing mortality among diabetics, the future simulated prevalence will be too high, leading to overestimated morbidity and health system cost impacts of interventions.

To satisfy all these requirements, the MSLT needs the following mortality rates:

1. DM-coded mortality by sex, age and ethnic group in 2011 (i.e. Mx[DM-coded]), operationalized as DM-coded CFR among diabetics (i.e. Mx[DM-coded | DM] = CFR[DM-coded] = Mx[DM-coded] / p, where p is the prevalence of diabetes in the given sex by age by ethnic group). Neither is problematic to calculate, being just that mortality rate in the sex by age by ethnic population coded as diabetes, and this same entity divided by the prevalence of DM to calculate the CFR.
2. Excess all-cause mortality among diabetics, being that excess to all-cause mortality without diabetes. It is this mortality rate that is used to 'kill people off' in the DM state so as to maintain the prevalence of people with DM at the 'correct' level. Call this CFR_{excess}[All-cause|DM]. It is first estimated directly from VDR data linked to mortality data (see method below), and then inputted to DISMOD to ensure coherence with prevalence data.
3. Because we assume different future trends in CHD, stroke and DM-coded CRFs within the MSLT, and also because we wish to allow for additional trends in CHD and stroke CFR among diabetics to match trends occurring in the CHD and stroke states themselves, we disaggregate the CFR_{excess}[All-cause|DM] into four components for all sex by age by ethnic groups in base-year 2011:
    a. CFR[DM-coded], i.e. as above

b. CFR_{excess}[CHD|DM]
c. CFR_{excess}[Stroke|DM]
d. CFR_{excess}[Other|DM], i.e. the excess CFR among diabetics for causes of death other than DM-coded, CHD and stroke.

(iii) Excess all-cause mortality among diabetics; CFR_{excess}[All-cause|DM]

This entity cannot be estimated directly, but rather requires calculation from the all-cause mortality in the whole population, all-cause mortality among diabetics, and prevalence of diabetes.

First:

$$\mathrm{Mx}[all\_cause|\overline{DM}] = \frac{(Mx - p \times Mx[all\_cause|DM])}{1-p} \tag{6}$$

where:

$\mathrm{Mx}[all\_cause|\overline{DM}]$ = all-cause mortality rate among people without diabetes

Mx = average or background mortality rate in total population

p = proportion of people with DM

$Mx[all\_cause|DM]$ = all-cause mortality rate among people with diabetes, or the crude (not yet excess) CFR among DM.

Then:

$$\mathrm{CFR}_{excess}[\mathrm{DM}] = Mx[all_{cause}|DM] - \mathrm{Mx}[all\_cause|\overline{DM}] \tag{7}$$

where:

$\mathrm{CFR}_{excess}[\text{All cause}|\text{DM}]$ = excess case fatality rate among people with DM (to be used as the equivalent of the CFR in DISMOD)

And:

$$\mathrm{Mx}_{excess}[\mathrm{DM}] = \mathrm{CFR}_{excess}[\text{All cause}|\text{DM}] \times \mathrm{p} \tag{8}$$

where:

$\mathrm{Mx}_{excess}[\mathrm{DM}]$ = population mortality rate attributable to DM.

For example, imagine that the prevalence of DM for a given sex by ethnic by age group was 0.2, the average population mortality rate was 250 per 100,000, and the all-cause mortality rate among diabetics was 450 per 100,000. Then:

$$\mathrm{Mx}[all\_cause|\overline{DM}] = \frac{(250 - 0.2 \times 450)}{1 - 0.2}$$

= 200 per 100,000

(9)

And:

$$\mathrm{CFR}_{excess}[\mathrm{DM}] = 450 - 200$$

= 250 per 100,000

(10)

And:

$$\mathrm{Mx}_{excess}[\mathrm{DM}] = 250 \times 0.2$$

= 50 per 100,000

(11)

(iv) DISMOD

For any given disease, the following parameters are mathematically related: incidence, duration, prevalence, case fatality. Therefore, if estimates of (some of) these parameter are estimated, they may not be mathematically coherent as a system. In our example, the VDR case definition of who was a diabetic may have some (differential over time) misclassification bias, meaning that the incidence rates are (somewhat) biased.

DISMOD II is an epidemiological tool[45] that takes in sets of these parameters, and outputs a coherent set of the same input parameters (plus those from the above list for which input data was missing). The input and output estimates should – of course – be close, acting as a check.

Treating diabetes as the disease of interest, we inputted the following parameters (for 2011):

- Prevalence (see above)
- Remission rate set at zero (i.e. assumption that once you have diabetes, you have diabetes forever)
- Case fatality (see above) or Excess DM mortality to that in general population = Mx[all-cause|DM] – Mx$[all_cause|\overline{DM}]$ =Excess all-cause mortality rate among diabetics
- Population mortality rate due to DM (see above)
- And, as is required, the all-cause mortality rate in the general population.

- Note: Incidence rate inputs were ignored in DISMOD

The outputs were assessed for coherence, and used as inputs to parametrizing the DM disease process.

(v) Excess CHD, stroke and 'other' CFR among diabetics; all-cause mortality among diabetics; CFR_{excess}[CHD|DM], CFR_{excess}[Stroke|DM] and CFR_{excess}[Other|DM]

To retain coherence with the above 'envelope' $\mathrm{CFR}_{excess}[\mathrm{DM}]$, and the directly estimated CFR[DM-coded], we simply proportionately disaggregate $(\mathrm{CFR}_{excess}[\mathrm{DM}] - \mathrm{CFR}[\mathrm{DM} - \mathrm{coded}])$ into that due to CHD, stroke and 'Other'.

To generate those proportions, we first directly calculate CFR_{excess}[CHD|DM], CFR_{excess}[Stroke|DM] and CFR_{excess}[Other|DM] in the same way as $\mathrm{CFR}_{excess}[\mathrm{DM}]$ was above – simply substituting $Mx[all\_cause|DM]$:

- Mx $[all\_cause|\overline{DM}]$ for population cause-specific mortality rates
- $\mathrm{Mx[all\_cause|DM]}$ for mortality rates of CHD, stroke and 'other' among diabetics (i.e. their respective crude CFRs).

Having calculated these three entities for each sex by age by ethnic group in 2011, the proportionate distribution across the three is calculated, and used to disaggregate $\mathrm{CFR}_{excess}[\mathrm{DM}] - \mathrm{CFR}[\mathrm{DM} - \mathrm{coded}]$.

4.01.2. Case fatalities operationalized in DM disease process

For clarity, the case fatality rates included in the DM disease process of the MSLT were:

- Mx[all-cause|DM] – Mx. Or the sum of 'O+C+S' in Figure 8 (page 42). This was used as the absorbing death state within the DM disease process.

- Mx[all-cause|DM] – Mx – (Mx[CHD|DM] ^{excess} + Mx[Stroke|DM] ^{excess}). Or 'O' in Figure 8 (page 42). This case fatality generated the mortality difference (between the BAU and intervention) in the DM that was then 'added to' the all-cause mortality rate in the main lifetable. CHD and stroke deaths were excluded from the mortality rate linked to the main lifetable from the DM state, as this mortality was captured in the CHD and stroke disease processes (with the DM state acting as a risk factor to change incidence inflow to the CHD and stroke states).

4.01.3. Preventing double-counting of BMI effects on DM and on CHD and stroke

BMI is a risk factor for diabetes, CHD and stroke. However, diabetes is itself also a risk factor for CHD and stroke. This is illustrated for CHD in Figure 17 (page 96).

Although diseases in the BODE[3] DIET MSLT model are assumed to be independent, we added a link between changing diabetes prevalence and CHD and stroke incidence, using relative risks from systematic reviews of cohort studies by Peters et al[75,76] to quantify the increased risk of CHD and

stroke in diabetics (Pathway B_2 in Figure 17: The relationship between BMI, diabetes and CHD, page 94)

To then prevent double-counting of CHD and stroke effects we determined the relative risks of CHD and stroke associated with changes in BMI that would not be mediated by diabetes (Pathway A in Figure 17). Since there are no published estimates of these RRs, we derived them using the GRG nonlinear method of optimisation in Excel, assuming that the fractions of the disease attributable to BMI directly (Pathway A in Figure 17) and indirectly via diabetes (Pathway B in Figure 17) must sum to the total attributable fraction (Pathway C in Figure 17).

Figure 17: The relationship between BMI, diabetes and CHD

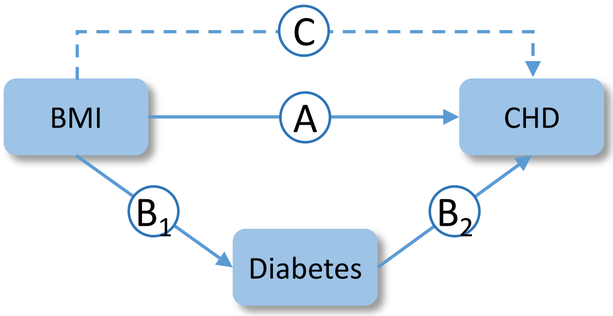

Appendix E: Relative risks of diet to disease associations

Table 19: RRs of BMI-related diseases (non-cancers) from the GBD study[38]* (RR per 5 BMI unit increase, TMREL: 21-23)

	Age-group	CHD	Ischaemic stroke	Haemorrhagic stroke	Type 2 diabetes	Osteoarthritis (knee and hip combined)
Male	25-29	2.274 (1.252 - 3.686)	2.472 (1.398 - 3.979)	3.066 (1.750 - 5.337)	3.546 (2.300 - 5.227)	1.570 (1.327 - 1.860)
	30-34	2.018 (1.291 - 3.107)	2.235 (1.444 - 3.333)	2.913 (1.857 - 4.398)	3.455 (2.500 - 4.692)	1.573 (1.308 - 1.890)
	35-39	1.724 (1.531 - 1.934)	1.979 (1.689 - 2.313)	2.598 (1.974 - 3.385)	3.349 (2.801 - 3.918)	1.573 (1.317 - 1.890)
	40-44	1.599 (1.417 - 1.785)	1.826 (1.599 - 2.076)	2.389 (1.869 - 3.001)	3.160 (2.689 - 3.700)	1.577 (1.313 - 1.878)
	45-49	1.567 (1.455 - 1.681)	1.733 (1.580 - 1.899)	2.199 (1.819 - 2.673)	2.864 (2.450 - 3.318)	1.575 (1.310 - 1.902)
	50-54	1.520 (1.416 - 1.631)	1.635 (1.479 - 1.797)	1.996 (1.625 - 2.420)	2.624 (2.222 - 3.038)	1.561 (1.295 - 1.888)
	55-59	1.466 (1.372 - 1.558)	1.543 (1.440 - 1.653)	1.805 (1.573 - 2.062)	2.417 (2.084 - 2.781)	1.562 (1.310 - 1.879)
	60-64	1.414 (1.324 - 1.505)	1.455 (1.345 - 1.566)	1.665 (1.437 - 1.932)	2.215 (1.866 - 2.611)	1.566 (1.306 - 1.866)
	65-69	1.364 (1.286 - 1.448)	1.380 (1.309 - 1.458)	1.523 (1.376 - 1.686)	2.046 (1.724 - 2.388)	1.566 (1.230 - 1.878)
	70-74	1.319 (1.241 - 1.400)	1.304 (1.233 - 1.377)	1.410 (1.263 - 1.571)	1.896 (1.596 - 2.229)	1.568 (1.307 - 1.899)
	75-79	1.274 (1.187 - 1.365)	1.228 (1.159 - 1.305)	1.295 (1.162 - 1.439)	1.740 (1.445 - 2.087)	1.571 (1.296 - 1.880)
	80+	1.170 (1.090 - 1.252)	1.068 (1.000 - 1.143)	1.071 (1.000 - 1.220)	1.461 (1.207 - 1.762)	1.571 (1.310 - 1.897)
Female	25-29	2.274 (1.252 - 3.686)	2.472 (1.398 - 3.979)	3.066 (1.75 - 5.337)	3.546 (2.300 - 5.227)	1.570 (1.327 - 1.860)
	30-34	2.018 (1.291 - 3.107)	2.235 (1.444 - 3.333)	2.913 (1.857 - 4.398)	3.455 (2.500 - 4.692)	1.573 (1.308 - 1.890)
	35-39	1.724 (1.531 - 1.934)	1.979 (1.689 - 2.313)	2.598 (1.974 - 3.385)	3.349 (2.801 - 3.918)	1.573 (1.317 - 1.890)
	40-44	1.599 (1.417 - 1.785)	1.826 (1.599 - 2.076)	2.389 (1.869 - 3.001)	3.160 (2.689 - 3.700)	1.577 (1.313 - 1.878)
	45-49	1.567 (1.455 - 1.681)	1.733 (1.580 - 1.899)	2.199 (1.819 - 2.673)	2.864 (2.450 - 3.318)	1.575 (1.310 - 1.902)
	50-54	1.520 (1.416 - 1.631)	1.635 (1.479 - 1.797)	1.996 (1.625 - 2.420)	2.624 (2.222 - 3.038)	1.561 (1.295 - 1.888)
	55-59	1.466 (1.372 - 1.558)	1.543 (1.440 - 1.653)	1.805 (1.573 - 2.062)	2.417 (2.084 - 2.781)	1.562 (1.310 - 1.879)
	60-64	1.414 (1.324 - 1.505)	1.455 (1.345 - 1.566)	1.665 (1.437 - 1.932)	2.215 (1.866 - 2.611)	1.566 (1.306 - 1.866)
	65-69	1.364 (1.286 - 1.448)	1.380 (1.309 - 1.458)	1.523 (1.376 - 1.686)	2.046 (1.724 - 2.388)	1.566 (1.300 - 1.878)
	70-74	1.319 (1.241 - 1.400)	1.304 (1.233 - 1.377)	1.410 (1.263 - 1.571)	1.896 (1.596 - 2.229)	1.568 (1.307 - 1.899)
	75-79	1.274 (1.187 - 1.365)	1.228 (1.159 - 1.305)	1.295 (1.162 - 1.439)	1.740 (1.445 - 2.087)	1.571 (1.300 - 1.880)
	80+	1.170 (1.090 - 1.252)	1.068 (1.000 - 1.143)	1.071 (1.000 - 1.220)	1.461 (1.207 - 1.762)	1.571 (1.310 - 1.897)

*RRs were the same in the GBD paper published in 2016[77]

Table 20: RRs of BMI-related cancers from the GBD study[38]* (RR per 5 BMI unit increase, TMREL: 21-23)

		Kidney cancer	Liver cancer	Oesophagus cancer	Pancreas cancer	Thyroid cancer	Colorectal cancer	Gallbladder cancer	Endometrial Cancer	Breast cancer	Ovarian cancer
Male	All ages	1.240 (1.171 - 1.313)	1.289 (1.108 - 1.492)	1.391 (1.075 - 1.763)	1.071 (1.000 - 1.154)	1.221 (1.067 - 1.384)	1.177 (1.145 - 1.208)	1.155 (1.033 - 1.282)	N/A	N/A	N/A
Female	All ages	1.320 (1.253 - 1.395)	1.176 (1.03 - 1.335)	1.351 (1.012 - 1.745)	1.092 (1.037 - 1.144)	1.136 (1.094 - 1.178)	1.059 (1.031 - 1.083)	1.344 (1.223 - 1.478)	1.613 (1.542 - 1.682)	1.023 (1.020 - 1.026)	1.038 (1.000 - 1.078)

*RRs were the same in the GBD paper published in 2016[77]

Table 21: RRs of diseases associated with low fruit intake from the GBD study[38]* (per 100 grams reduction in fruit intake, TMREL: 200-400g)

	Age-group	CHD	Ischaemic stroke	Haemorrhagic stroke	Head & neck cancer*	Lung cancer	Oesophagus cancer
Male	25-29	1.174 (1.075 - 1.270)	1.235 (1.123 - 1.355)	1.732 (1.309 - 2.294)	1.042 (1.000 - 1.092)	1.075 (1.028 - 1.124)	1.151 (1.031 - 1.286)
	30-34	1.164 (1.067 - 1.255)	1.223 (1.123 - 1.338)	1.683 (1.273 - 2.211)			
	35-39	1.155 (1.079 - 1.244)	1.205 (1.104 - 1.316)	1.629 (1.265 - 2.064)			
	40-44	1.143 (1.068 - 1.223)	1.194 (1.099 - 1.295)	1.577 (1.241 - 1.969)			
	45-49	1.129 (1.054 - 1.203)	1.177 (1.098 - 1.259)	1.516 (1.216 - 1.844)			
	50-54	1.117 (1.052 - 1.184)	1.161 (1.089 - 1.240)	1.471 (1.213 - 1.783)			
	55-59	1.107 (1.052 - 1.163)	1.146 (1.079 - 1.214)	1.425 (1.186 - 1.700)			
	60-64	1.098 (1.047 - 1.149)	1.132 (1.073 - 1.195)	1.375 (1.171 - 1.608)			
	65-69	1.088 (1.041 - 1.142)	1.117 (1.063 - 1.175)	1.332 (1.150 - 1.538)			
	70-74	1.077 (1.035 - 1.118)	1.103 (1.057 - 1.156)	1.286 (1.131 - 1.449)			
	75-79	1.066 (1.031 - 1.103)	1.089 (1.049 - 1.127)	1.245 (1.106 - 1.384)			
	80+	1.052 (1.040 - 1.062)	1.069 (1.057 - 1.082)	1.188 (1.145 - 1.233)			
Female	25-29	1.174 (1.075 - 1.270)	1.235 (1.123 - 1.355)	1.732 (1.309 - 2.294)	1.042 (1.000 - 1.092)	1.075 (1.028 - 1.124)	1.151 (1.031 - 1.286)
	30-34	1.164 (1.067 - 1.255)	1.223 (1.123 - 1.338)	1.683 (1.273 - 2.211)			
	35-39	1.155 (1.079 - 1.244)	1.205 (1.104 - 1.316)	1.629 (1.265 - 2.064)			
	40-44	1.143 (1.068 - 1.223)	1.194 (1.099 - 1.295)	1.577 (1.241 - 1.969)			
	45-49	1.129 (1.054 - 1.203)	1.177 (1.098 - 1.259)	1.516 (1.216 - 1.844)			
	50-54	1.117 (1.052 - 1.184)	1.161 (1.089 - 1.240)	1.471 (1.213 - 1.783)			
	55-59	1.107 (1.052 - 1.163)	1.146 (1.079 - 1.214)	1.425 (1.186 - 1.700)			
	60-64	1.098 (1.047 - 1.149)	1.132 (1.073 - 1.195)	1.375 (1.171 - 1.608)			
	65-69	1.088 (1.041 - 1.142)	1.117 (1.063 - 1.175)	1.332 (1.150 - 1.538)			
	70-74	1.077 (1.035 - 1.118)	1.103 (1.057 - 1.156)	1.286 (1.131 - 1.449)			
	75-79	1.066 (1.031 - 1.103)	1.089 (1.049 - 1.127)	1.245 (1.106 - 1.384)			
	80+	1.052 (1.040 - 1.062)	1.068 (1.056 - 1.081)	1.187 (1.145 - 1.231)			

*The RRs used here were an average of the RRs in the GBD for cancers of the larynx, nasopharynx and other pharynx and mouth.

*RRs were the same in the GBD paper published in 2016[77]

Table 22: RRs of diseases associated with low vegetable intake and high SSB intake from the GBDstudy[38]*

		Vegetables (decrease of 100 g/day, TMREL: 350-450g)			SSBs (increase of 226.8 g/day, TMREL: 0-64.3g)	
	**Age-group**	**CHD**	**Ischaemic stroke**	**Haemorrhagic stroke**	**Type 2 diabetes**	**Ovarian cancer**
Male	25-29	1.129 (1.068 - 1.190)	1.222 (1.047 - 1.429)	1.392 (1.084 - 1.764)	1.462 (1.222 - 1.751)	N/A
	30-34	1.117 (1.062 - 1.171)	1.206 (1.048 - 1.375)	1.353 (1.080 - 1.672)	1.426 (1.182 - 1.696)	
	35-39	1.111 (1.056 - 1.162)	1.193 (1.051 - 1.341)	1.344 (1.076 - 1.675)	1.392 (1.187 - 1.624)	
	40-44	1.103 (1.052 - 1.157)	1.178 (1.038 - 1.338)	1.310 (1.076 - 1.585)	1.360 (1.169 - 1.586)	
	45-49	1.096 (1.051 - 1.141)	1.163 (1.036 - 1.302)	1.289 (1.075 - 1.542)	1.332 (1.151 - 1.537)	
	50-54	1.086 (1.046 - 1.129)	1.148 (1.031 - 1.269)	1.257 (1.071 - 1.490)	1.297 (1.137 - 1.478)	
	55-59	1.079 (1.043 - 1.117)	1.132 (1.025 - 1.249)	1.235 (1.056 - 1.444)	1.271 (1.126 - 1.424)	
	60-64	1.073 (1.040 - 1.108)	1.124 (1.030 - 1.221)	1.212 (1.046 - 1.398)	1.238 (1.117 - 1.377)	
	65-69	1.064 (1.035 - 1.093)	1.109 (1.026 - 1.194)	1.187 (1.046 - 1.356)	1.214 (1.101 - 1.332)	
	70-74	1.056 (1.030 - 1.083)	1.097 (1.024 - 1.177)	1.166 (1.047 - 1.304)	1.188 (1.085 - 1.292)	
	75-79	1.049 (1.028 - 1.073)	1.083 (1.018 - 1.150)	1.140 (1.035 - 1.254)	1.160 (1.073 - 1.251)	
	80+	1.038 (1.031 - 1.045)	1.065 (1.045 - 1.085)	1.109 (1.073 - 1.144)	1.123 (1.095 - 1.151)	
Female	25-29	1.129 (1.068 - 1.190)	1.222 (1.047 - 1.429)	1.392 (1.084 - 1.764)	1.462 (1.222 - 1.751)	1.001 (1.000 - 1.002)
	30-34	1.117 (1.062 - 1.171)	1.206 (1.048 - 1.375)	1.353 (1.080 - 1.672)	1.426 (1.182 - 1.696)	1.001 (1.000 - 1.002)
	35-39	1.111 (1.056 - 1.162)	1.193 (1.051 - 1.341)	1.344 (1.076 - 1.675)	1.392 (1.187 - 1.624)	1.001 (1.000 - 1.003)
	40-44	1.103 (1.052 - 1.157)	1.178 (1.038 - 1.338)	1.310 (1.076 - 1.585)	1.360 (1.169 - 1.586)	1.001 (1.000 - 1.003)
	45-49	1.096 (1.051 - 1.141)	1.163 (1.036 - 1.302)	1.289 (1.075 - 1.542)	1.332 (1.151 - 1.537)	1.001 (1.000 - 1.003)
	50-54	1.086 (1.046 - 1.129)	1.148 (1.031 - 1.269)	1.257 (1.071 - 1.490)	1.297 (1.137 - 1.478)	1.001 (1.000 - 1.003)
	55-59	1.079 (1.043 - 1.117)	1.132 (1.025 - 1.249)	1.235 (1.056 - 1.444)	1.271 (1.126 - 1.424)	1.001 (1.000 - 1.003)
	60-64	1.073 (1.040 - 1.108)	1.124 (1.030 - 1.221)	1.212 (1.046 - 1.398)	1.238 (1.117 - 1.377)	1.001 (1.000 - 1.003)
	65-69	1.064 (1.035 - 1.093)	1.109 (1.026 - 1.194)	1.187 (1.046 - 1.356)	1.214 (1.101 - 1.332)	1.001 (1.000 - 1.003)
	70-74	1.056 (1.030 - 1.083)	1.097 (1.024 - 1.177)	1.166 (1.047 - 1.304)	1.188 (1.085 - 1.292)	1.001 (1.000 - 1.003)
	75-79	1.049 (1.028 - 1.073)	1.083 (1.018 - 1.150)	1.140 (1.035 - 1.254)	1.160 (1.073 - 1.251)	1.001 (1.000 - 1.003)
	80+	1.038 (1.031 - 1.045)	1.064 (1.044 - 1.086)	1.110 (1.074 - 1.146)	1.122 (1.096 - 1.149)	1.001 (1.000 - 1.003)

*RRs were the same in the GBD paper published in 2016[77]

Table 23: RRs of diseases associated with high sodium intake and low PUFA intakes from the GBD study[38]*

		Sodium intake (increase of 1 gram/day, TMREL 1-5g)				PUFA intake (decrease of 5% TE, TMREL: 10-15% TE)
	**Age-group**	**CHD**	**Ischaemic stroke**	**Haemorrhagic stroke**	**Stomach cancer**	**CHD**
Male	25-29	1.044 (1.009 - 1.091)	1.056 (1.014 - 1.106)	1.058 (1.016 - 1.113)	1.199 (1.000 - 1.444)	1.148 (1.059 - 1.241)
	30-34	1.054 (1.023 - 1.092)	1.074 (1.037 - 1.117)	1.079 (1.037 - 1.130)	1.205 (1.007 - 1.430)	1.140 (1.057 - 1.231)
	35-39	1.060 (1.034 - 1.093)	1.090 (1.053 - 1.126)	1.097 (1.055 - 1.147)	1.205 (1.000 - 1.462)	1.130 (1.050 - 1.214)
	40-44	1.067 (1.040 - 1.100)	1.103 (1.065 - 1.140)	1.112 (1.068 - 1.158)	1.202 (1.000 - 1.443)	1.120 (1.045 - 1.194)
	45-49	1.077 (1.047 - 1.110)	1.112 (1.075 - 1.149)	1.121 (1.077 - 1.175)	1.209 (1.000 - 1.448)	1.112 (1.044 - 1.180)
	50-54	1.084 (1.054 - 1.118)	1.118 (1.082 - 1.156)	1.127 (1.079 - 1.187)	1.198 (1.000 - 1.431)	1.101 (1.042 - 1.166)
	55-59	1.089 (1.060 - 1.124)	1.121 (1.086 - 1.157)	1.128 (1.081 - 1.184)	1.204 (1.006 - 1.430)	1.093 (1.035 - 1.155)
	60-64	1.091 (1.056 - 1.131)	1.120 (1.087 - 1.156)	1.126 (1.070 - 1.187)	1.200 (1.000 - 1.459)	1.084 (1.034 - 1.134)
	65-69	1.092 (1.059 - 1.130)	1.117 (1.083 - 1.152)	1.122 (1.072 - 1.175)	1.206 (1.003 - 1.432)	1.075 (1.030 - 1.123)
	70-74	1.083 (1.051 - 1.119)	1.100 (1.072 - 1.130)	1.103 (1.062 - 1.149)	1.210 (1.000 - 1.446)	1.066 (1.026 - 1.108)
	75-79	1.073 (1.033 - 1.114)	1.081 (1.056 - 1.109)	1.083 (1.036 - 1.144)	1.203 (1.000 - 1.435)	1.057 (1.023 - 1.094)
	80+	1.057 (1.021 - 1.098)	1.040 (1.021 - 1.063)	1.043 (1.000 - 1.097)	1.205 (1.000 - 1.460)	1.045 (1.034 - 1.056)
Female	25-29	1.040 (1.007 - 1.085)	1.051 (1.010 - 1.101)	1.053 (1.012 - 1.108)	1.199 (1.000 - 1.444)	1.148 (1.059 - 1.241)
	30-34	1.050(1.019 - 1.090)	1.068 (1.030 - 1.112)	1.072 (1.031 - 1.124)	1.205 (1.007 - 1.430)	1.140 (1.057 - 1.231)
	35-39	1.057 (1.029 - 1.091)	1.084 (1.046 - 1.124)	1.091 (1.048 - 1.141)	1.205 (1.000 - 1.462)	1.130 (1.050 - 1.214)
	40-44	1.063 (1.036 - 1.096)	1.097 (1.058 - 1.135)	1.106 (1.060 - 1.154)	1.202 (1.000 - 1.443)	1.120 (1.045 - 1.194)
	45-49	1.073 (1.044 - 1.108)	1.107 (1.069 - 1.146)	1.116 (1.071 - 1.171)	1.209 (1.000 - 1.448)	1.112 (1.044 - 1.180)
	50-54	1.082 (1.050 - 1.118)	1.114 (1.076 - 1.154)	1.123 (1.074 - 1.185)	1.198 (1.000 - 1.431)	1.101 (1.042 - 1.166)
	55-59	1.088 (1.057 - 1.123)	1.119 (1.083 - 1.156)	1.127 (1.079 - 1.181)	1.204 (1.006 - 1.430)	1.093 (1.035 - 1.155)
	60-64	1.091 (1.056 - 1.132)	1.121 (1.085 - 1.157)	1.126 (1.068 - 1.188)	1.200 (1.000 - 1.459)	1.084 (1.034 - 1.134)
	65-69	1.093 (1.059 - 1.130)	1.118 (1.083 - 1.152)	1.123 (1.075 - 1.180)	1.206 (1.003 - 1.432)	1.075 (1.030 - 1.123)
	70-74	1.084 (1.050 - 1.120)	1.101 (1.074 - 1.130)	1.105 (1.064 - 1.153)	1.210 (1.000 - 1.446)	1.066 (1.026 - 1.108)
	75-79	1.074 (1.034 - 1.117)	1.082 (1.058 - 1.110)	1.084 (1.037 - 1.145)	1.203 (1.000 - 1.435)	1.057 (1.023 - 1.094)
	80+	1.058 (1.022 - 1.098)	1.041 (1.022 - 1.064)	1.044 (1.000 - 1.097)	1.205 (1.000 - 1.460)	1.044 (1.033 - 1.055)

*RRs were the same in the GBD paper published in 2016[77]

Appendix F: Health system costs

This Appendix provides tables of the health system cost inputs into the BODE^3 DIET MSLT models (See section 1.1.14 for details of how these are calculated and what A, B, C, D and E costs mean). All costs are in NZ$ for the baseline year of 2011. The specific details and equations of how these costs are calculated are detailed in the following online Report: "Kvizhinadze G, Nghiem N, Atkinson J, Blakely T. Cost Off-Sets Used in BODE3 Multistate Lifetable Models Burden of Disease Epidemiology, Equity and Cost-Effectiveness Programme (BODE3). Technical Report: Number 15. Wellington: University of Otago, Wellington, 2016" (at: http://www.otago.ac.nz/wellington/otago619391.pdf).

Table 24: Population health system costs

		Population wide †		Population without diet-related disease	
Sex	Age group	Not the last six months of life	Last six months of life	A	B
Male	<1	$5,814	$91,146	$5,797	$88,383
	1-4	$1,449	$32,496	$1,443	$31,540
	5-9	$707	$30,726	$701	$30,440
	10-14	$684	$23,793	$671	$23,157
	15-19	$721	$15,886	$703	$15,495
	20-24	$762	$9,334	$739	$8,449
	25-29	$773	$14,157	$731	$13,156
	30-34	$851	$16,567	$789	$14,157
	35-39	$948	$15,514	$842	$13,091
	40-44	$1,142	$19,908	$931	$18,394
	45-49	$1,396	$21,775	$1,054	$20,302
	50-54	$1,737	$22,548	$1,170	$23,093
	55-59	$2,195	$24,272	$1,340	$24,516
	60-64	$2,813	$23,628	$1,617	$24,630
	65-69	$3,796	$26,128	$2,172	$26,936
	70-74	$4,789	$23,822	$2,720	$23,743
	75-79	$5,590	$22,807	$3,337	$21,893
	80-84	$6,150	$18,801	$4,025	$17,314
	85-89	$6,512	$17,325	$4,599	$15,142
	90-94	$6,388	$14,055	$4,831	$11,359
	95-99	$5,804	$10,973	$4,797	$8,424
Female	<1	$5,001	$82,445	$4,996	$79,821
	1-4	$1,251	$31,830	$1,246	$30,735
	5-9	$634	$24,762	$629	$25,150
	10-14	$611	$22,590	$598	$22,594
	15-19	$956	$18,796	$931	$17,351
	20-24	$1,267	$24,471	$1,231	$23,198
	25-29	$1,398	$22,308	$1,336	$20,752
	30-34	$1,585	$26,061	$1,486	$20,649

35-39	$1,502	$28,721	$1,355	$22,192
40-44	$1,357	$29,407	$1,125	$28,042
45-49	$1,520	$27,308	$1,151	$25,860
50-54	$1,761	$28,725	$1,241	$27,427
55-59	$2,076	$28,720	$1,345	$26,957
60-64	$2,572	$25,987	$1,569	$25,462
65-69	$3,379	$27,254	$2,027	$26,401
70-74	$4,084	$24,278	$2,436	$22,813
75-79	$4,836	$20,716	$2,968	$19,312
80-84	$5,401	$16,625	$3,584	$14,704
85-89	$5,732	$14,067	$4,115	$11,123
90-94	$5,632	$10,873	$4,462	$8,013
95-99	$4,808	$7,415	$3,973	$4,889

†Not actually used in the model, but used in calibration

Table 25: Disease costs for breast cancer

Sex	Age group	C	D	E
Females	<1	$38,556	$5,934	$38,207
	1-4	$38,556	$5,934	$38,207
	5-9	$38,556	$5,934	$38,207
	10-14	$38,556	$5,934	$38,207
	15-19	$38,556	$5,934	$38,207
	20-24	$38,556	$5,934	$38,207
	25-29	$38,556	$5,934	$38,207
	30-34	$38,556	$5,934	$38,207
	35-39	$38,556	$5,934	$38,207
	40-44	$38,556	$5,934	$38,207
	45-49	$24,212	$2,769	$29,496
	50-54	$24,212	$2,769	$29,496
	55-59	$24,212	$2,769	$29,496
	60-64	$24,212	$2,769	$29,496
	65-69	$15,293	$2,197	$25,749
	70-74	$15,293	$2,197	$25,749
	75-79	$11,297	$2,053	$20,786
	80-84	$11,297	$2,053	$20,786
	85-89	$5,950	$1,559	$11,024
	90-94	$5,950	$1,559	$11,024
	95-99	$5,950	$1,559	$11,024

Table 26: Disease costs for CHD

Sex	Age group	C	D	E
Male	<1	$18,330	$3,953	$11,729
	1-4	$18,330	$3,953	$11,729
	5-9	$18,330	$3,953	$11,729
	10-14	$18,330	$3,953	$11,729
	15-19	$18,330	$3,953	$11,729
	20-24	$18,330	$3,953	$11,729
	25-29	$18,330	$3,953	$11,729
	30-34	$18,330	$3,953	$11,729
	35-39	$18,330	$3,953	$11,729
	40-44	$18,330	$3,953	$11,729
	45-49	$15,394	$3,232	$10,222
	50-54	$15,394	$3,232	$10,222
	55-59	$15,394	$3,232	$10,222
	60-64	$15,394	$3,232	$10,222
	65-69	$12,883	$3,197	$14,709
	70-74	$12,883	$3,197	$14,709
	75-79	$9,152	$2,848	$14,291
	80-84	$9,152	$2,848	$14,291
	85-89	$5,620	$2,346	$11,084
	90-94	$5,620	$2,346	$11,084
	95-99	$5,620	$2,346	$11,084
Females	<1	$16,043	$4,114	$14,599
	1-4	$16,043	$4,114	$14,599
	5-9	$16,043	$4,114	$14,599
	10-14	$16,043	$4,114	$14,599
	15-19	$16,043	$4,114	$14,599
	20-24	$16,043	$4,114	$14,599
	25-29	$16,043	$4,114	$14,599
	30-34	$16,043	$4,114	$14,599
	35-39	$16,043	$4,114	$14,599
	40-44	$16,043	$4,114	$14,599
	45-49	$11,643	$4,076	$17,180
	50-54	$11,643	$4,076	$17,180
	55-59	$11,643	$4,076	$17,180
	60-64	$11,643	$4,076	$17,180
	65-69	$9,446	$3,432	$17,872
	70-74	$9,446	$3,432	$17,872
	75-79	$6,687	$2,896	$13,593
	80-84	$6,687	$2,896	$13,593
	85-89	$4,583	$2,073	$9,165
	90-94	$4,583	$2,073	$9,165
	95-99	$4,583	$2,073	$9,165

Table 27: Disease costs for colorectal cancer

Sex	Age group	C	D	E
Male	<1	$44,852	$4,290	$36,026
	1-4	$44,852	$4,290	$36,026
	5-9	$44,852	$4,290	$36,026
	10-14	$44,852	$4,290	$36,026
	15-19	$44,852	$4,290	$36,026
	20-24	$44,852	$4,290	$36,026
	25-29	$44,852	$4,290	$36,026
	30-34	$44,852	$4,290	$36,026
	35-39	$44,852	$4,290	$36,026
	40-44	$44,852	$4,290	$36,026
	45-49	$31,791	$3,757	$29,511
	50-54	$31,791	$3,757	$29,511
	55-59	$31,791	$3,757	$29,511
	60-64	$31,791	$3,757	$29,511
	65-69	$25,679	$3,372	$28,407
	70-74	$25,679	$3,372	$28,407
	75-79	$19,293	$2,689	$25,237
	80-84	$19,293	$2,689	$25,237
	85-89	$13,760	$1,874	$17,245
	90-94	$13,760	$1,874	$17,245
	95-99	$13,760	$1,874	$17,245
Females	<1	$44,078	$4,632	$31,628
	1-4	$44,078	$4,632	$31,628
	5-9	$44,078	$4,632	$31,628
	10-14	$44,078	$4,632	$31,628
	15-19	$44,078	$4,632	$31,628
	20-24	$44,078	$4,632	$31,628
	25-29	$44,078	$4,632	$31,628
	30-34	$44,078	$4,632	$31,628
	35-39	$44,078	$4,632	$31,628
	40-44	$44,078	$4,632	$31,628
	45-49	$29,909	$3,138	$27,424
	50-54	$29,909	$3,138	$27,424
	55-59	$29,909	$3,138	$27,424
	60-64	$29,909	$3,138	$27,424
	65-69	$23,024	$2,709	$27,976
	70-74	$23,024	$2,709	$27,976
	75-79	$17,326	$2,165	$22,796
	80-84	$17,326	$2,165	$22,796
	85-89	$12,417	$1,587	$14,958
	90-94	$12,417	$1,587	$14,958
	95-99	$12,417	$1,587	$14,958

Table 28: Disease costs for diabetes

Sex	Age group	C	D	E
Male	<1	$2,892	$3,021	$46,215
	1-4	$2,892	$3,021	$46,215
	5-9	$2,892	$3,021	$46,215
	10-14	$2,892	$3,021	$46,215
	15-19	$2,892	$3,021	$46,215
	20-24	$2,892	$3,021	$46,215
	25-29	$2,892	$3,021	$46,215
	30-34	$2,892	$3,021	$46,215
	35-39	$2,892	$3,021	$46,215
	40-44	$2,892	$3,021	$46,215
	45-49	$2,244	$3,309	$46,215
	50-54	$2,244	$3,309	$46,215
	55-59	$2,244	$3,309	$46,215
	60-64	$2,244	$3,309	$46,215
	65-69	$2,611	$3,294	$21,516
	70-74	$2,611	$3,294	$21,516
	75-79	$2,601	$2,832	$23,472
	80-84	$2,601	$2,832	$23,472
	85-89	$2,423	$2,262	$25,429
	90-94	$2,423	$2,262	$25,429
	95-99	$2,423	$2,262	$25,429
Females	<1	$4,328	$3,073	$48,748
	1-4	$4,328	$3,073	$48,748
	5-9	$4,328	$3,073	$48,748
	10-14	$4,328	$3,073	$48,748
	15-19	$4,328	$3,073	$48,748
	20-24	$4,328	$3,073	$48,748
	25-29	$4,328	$3,073	$48,748
	30-34	$4,328	$3,073	$48,748
	35-39	$4,328	$3,073	$48,748
	40-44	$4,328	$3,073	$48,748
	45-49	$2,118	$3,206	$48,748
	50-54	$2,118	$3,206	$48,748
	55-59	$2,118	$3,206	$48,748
	60-64	$2,118	$3,206	$48,748
	65-69	$2,154	$3,131	$18,880
	70-74	$2,154	$3,131	$18,880
	75-79	$2,224	$2,611	$20,597
	80-84	$2,224	$2,611	$20,597
	85-89	$2,441	$1,915	$22,313
	90-94	$2,441	$1,915	$22,313
	95-99	$2,441	$1,915	$22,313

Table 29: Disease costs for endometrial cancer

Sex	Age group	C	D	E
Females	<1	$19,614	$2,795	$32,731
	1-4	$19,614	$2,795	$32,731
	5-9	$19,614	$2,795	$32,731
	10-14	$19,614	$2,795	$32,731
	15-19	$19,614	$2,795	$32,731
	20-24	$19,614	$2,795	$32,731
	25-29	$19,614	$2,795	$32,731
	30-34	$19,614	$2,795	$32,731
	35-39	$19,614	$2,795	$32,731
	40-44	$19,614	$2,795	$32,731
	45-49	$16,881	$2,488	$32,731
	50-54	$16,881	$2,488	$32,731
	55-59	$16,881	$2,488	$32,731
	60-64	$16,881	$2,488	$32,731
	65-69	$15,949	$2,332	$20,420
	70-74	$15,949	$2,332	$20,420
	75-79	$14,389	$1,848	$22,277
	80-84	$14,389	$1,848	$22,277
	85-89	$9,946	$1,554	$24,133
	90-94	$9,946	$1,554	$24,133
	95-99	$9,946	$1,554	$24,133

Table 30: Disease costs for gallbladder cancer

Sex	Age group	C	D	E
Male	<1	$41,128	$5,177	$22,808
	1-4	$41,128	$5,177	$22,808
	5-9	$41,128	$5,177	$22,808
	10-14	$41,128	$5,177	$22,808
	15-19	$41,128	$5,177	$22,808
	20-24	$41,128	$5,177	$22,808
	25-29	$41,128	$5,177	$22,808
	30-34	$41,128	$5,177	$22,808
	35-39	$41,128	$5,177	$22,808
	40-44	$41,128	$5,177	$22,808
	45-49	$41,128	$5,177	$22,808
	50-54	$41,128	$5,177	$22,808
	55-59	$41,128	$5,177	$22,808
	60-64	$41,128	$5,177	$22,808
	65-69	$29,213	$6,579	$27,869
	70-74	$29,213	$6,579	$27,869
	75-79	$29,213	$6,579	$30,403
	80-84	$29,213	$6,579	$30,403
	85-89	$29,213	$6,579	$32,937
	90-94	$29,213	$6,579	$32,937
	95-99	$29,213	$6,579	$32,937
Females	<1	$33,906	$3,367	$35,386
	1-4	$33,906	$3,367	$35,386
	5-9	$33,906	$3,367	$35,386
	10-14	$33,906	$3,367	$35,386
	15-19	$33,906	$3,367	$35,386
	20-24	$33,906	$3,367	$35,386
	25-29	$33,906	$3,367	$35,386
	30-34	$33,906	$3,367	$35,386
	35-39	$33,906	$3,367	$35,386
	40-44	$33,906	$3,367	$35,386
	45-49	$33,906	$3,367	$35,386
	50-54	$33,906	$3,367	$35,386
	55-59	$33,906	$3,367	$35,386
	60-64	$33,906	$3,367	$35,386
	65-69	$22,458	$2,722	$20,307
	70-74	$22,458	$2,722	$20,307
	75-79	$22,458	$2,722	$22,153
	80-84	$22,458	$2,722	$22,153
	85-89	$22,458	$2,722	$23,999
	90-94	$22,458	$2,722	$23,999
	95-99	$22,458	$2,722	$23,999

Table 31: Disease costs for head and neck cancer

Sex	Age group	C	D	E
Male	<1	$47,726	$4,575	$43,161
	1-4	$47,726	$4,575	$43,161
	5-9	$47,726	$4,575	$43,161
	10-14	$47,726	$4,575	$43,161
	15-19	$47,726	$4,575	$43,161
	20-24	$47,726	$4,575	$43,161
	25-29	$47,726	$4,575	$43,161
	30-34	$47,726	$4,575	$43,161
	35-39	$47,726	$4,575	$43,161
	40-44	$47,726	$4,575	$43,161
	45-49	$39,670	$3,446	$33,375
	50-54	$39,670	$3,446	$33,375
	55-59	$39,670	$3,446	$33,375
	60-64	$39,670	$3,446	$33,375
	65-69	$31,150	$4,346	$32,183
	70-74	$31,150	$4,346	$32,183
	75-79	$24,022	$3,337	$31,372
	80-84	$24,022	$3,337	$31,372
	85-89	$13,572	$4,471	$21,579
	90-94	$13,572	$4,471	$21,579
	95-99	$13,572	$4,471	$21,579
Females	<1	$40,457	$2,664	$32,227
	1-4	$40,457	$2,664	$32,227
	5-9	$40,457	$2,664	$32,227
	10-14	$40,457	$2,664	$32,227
	15-19	$40,457	$2,664	$32,227
	20-24	$40,457	$2,664	$32,227
	25-29	$40,457	$2,664	$32,227
	30-34	$40,457	$2,664	$32,227
	35-39	$40,457	$2,664	$32,227
	40-44	$40,457	$2,664	$32,227
	45-49	$37,492	$3,669	$32,227
	50-54	$37,492	$3,669	$32,227
	55-59	$37,492	$3,669	$32,227
	60-64	$37,492	$3,669	$32,227
	65-69	$31,035	$4,093	$22,097
	70-74	$31,035	$4,093	$22,097
	75-79	$19,854	$2,902	$24,105
	80-84	$19,854	$2,902	$24,105
	85-89	$16,066	$2,483	$26,114
	90-94	$16,066	$2,483	$26,114
	95-99	$16,066	$2,483	$26,114

Table 32: Disease costs for kidney cancer

Sex	Age group	C	D	E
Male	<1	$33,047	$4,017	$29,879
	1-4	$33,047	$4,017	$29,879
	5-9	$33,047	$4,017	$29,879
	10-14	$33,047	$4,017	$29,879
	15-19	$33,047	$4,017	$29,879
	20-24	$33,047	$4,017	$29,879
	25-29	$33,047	$4,017	$29,879
	30-34	$33,047	$4,017	$29,879
	35-39	$33,047	$4,017	$29,879
	40-44	$33,047	$4,017	$29,879
	45-49	$17,692	$4,482	$29,879
	50-54	$17,692	$4,482	$29,879
	55-59	$17,692	$4,482	$29,879
	60-64	$17,692	$4,482	$29,879
	65-69	$16,358	$4,328	$26,027
	70-74	$16,358	$4,328	$26,027
	75-79	$13,519	$3,871	$28,393
	80-84	$13,519	$3,871	$28,393
	85-89	$8,397	$1,932	$30,759
	90-94	$8,397	$1,932	$30,759
	95-99	$8,397	$1,932	$30,759
Females	<1	$32,878	$3,357	$37,101
	1-4	$32,878	$3,357	$37,101
	5-9	$32,878	$3,357	$37,101
	10-14	$32,878	$3,357	$37,101
	15-19	$32,878	$3,357	$37,101
	20-24	$32,878	$3,357	$37,101
	25-29	$32,878	$3,357	$37,101
	30-34	$32,878	$3,357	$37,101
	35-39	$32,878	$3,357	$37,101
	40-44	$32,878	$3,357	$37,101
	45-49	$17,978	$3,729	$37,101
	50-54	$17,978	$3,729	$37,101
	55-59	$17,978	$3,729	$37,101
	60-64	$17,978	$3,729	$37,101
	65-69	$15,677	$4,203	$20,097
	70-74	$15,677	$4,203	$20,097
	75-79	$13,905	$3,222	$21,924
	80-84	$13,905	$3,222	$21,924
	85-89	$7,970	$2,758	$23,751
	90-94	$7,970	$2,758	$23,751
	95-99	$7,970	$2,758	$23,751

Table 33: Disease costs for liver cancer

Sex	Age group	C	D	E
Male	<1	$74,020	$17,247	$18,979
	1-4	$74,020	$17,247	$18,979
	5-9	$74,020	$17,247	$18,979
	10-14	$74,020	$17,247	$18,979
	15-19	$74,020	$17,247	$18,979
	20-24	$74,020	$17,247	$18,979
	25-29	$74,020	$17,247	$18,979
	30-34	$74,020	$17,247	$18,979
	35-39	$74,020	$17,247	$18,979
	40-44	$74,020	$17,247	$18,979
	45-49	$34,485	$10,857	$18,979
	50-54	$34,485	$10,857	$18,979
	55-59	$34,485	$10,857	$18,979
	60-64	$34,485	$10,857	$18,979
	65-69	$24,283	$7,849	$17,609
	70-74	$24,283	$7,849	$17,609
	75-79	$12,098	$3,322	$19,209
	80-84	$12,098	$3,322	$19,209
	85-89	$12,098	$3,322	$20,810
	90-94	$12,098	$3,322	$20,810
	95-99	$12,098	$3,322	$20,810
Females	<1	$45,588	$10,304	$23,063
	1-4	$45,588	$10,304	$23,063
	5-9	$45,588	$10,304	$23,063
	10-14	$45,588	$10,304	$23,063
	15-19	$45,588	$10,304	$23,063
	20-24	$45,588	$10,304	$23,063
	25-29	$45,588	$10,304	$23,063
	30-34	$45,588	$10,304	$23,063
	35-39	$45,588	$10,304	$23,063
	40-44	$45,588	$10,304	$23,063
	45-49	$45,588	$7,986	$23,063
	50-54	$45,588	$7,986	$23,063
	55-59	$45,588	$7,986	$23,063
	60-64	$45,588	$7,986	$23,063
	65-69	$18,900	$6,708	$17,855
	70-74	$18,900	$6,708	$17,855
	75-79	$18,900	$3,374	$19,479
	80-84	$18,900	$3,374	$19,479
	85-89	$18,900	$3,374	$21,102
	90-94	$18,900	$3,374	$21,102
	95-99	$18,900	$3,374	$21,102

Table 34: Disease costs for lung cancer

Sex	Age group	C	D	E
Male	<1	$40,959	$4,495	$34,606
	1-4	$40,959	$4,495	$34,606
	5-9	$40,959	$4,495	$34,606
	10-14	$40,959	$4,495	$34,606
	15-19	$40,959	$4,495	$34,606
	20-24	$40,959	$4,495	$34,606
	25-29	$40,959	$4,495	$34,606
	30-34	$40,959	$4,495	$34,606
	35-39	$40,959	$4,495	$34,606
	40-44	$40,959	$4,495	$34,606
	45-49	$30,729	$5,538	$26,759
	50-54	$30,729	$5,538	$26,759
	55-59	$30,729	$5,538	$26,759
	60-64	$30,729	$5,538	$26,759
	65-69	$23,260	$5,196	$24,685
	70-74	$23,260	$5,196	$24,685
	75-79	$14,128	$3,863	$19,714
	80-84	$14,128	$3,863	$19,714
	85-89	$7,609	$2,237	$14,611
	90-94	$7,609	$2,237	$14,611
	95-99	$7,609	$2,237	$14,611
Females	<1	$37,542	$11,686	$32,434
	1-4	$37,542	$11,686	$32,434
	5-9	$37,542	$11,686	$32,434
	10-14	$37,542	$11,686	$32,434
	15-19	$37,542	$11,686	$32,434
	20-24	$37,542	$11,686	$32,434
	25-29	$37,542	$11,686	$32,434
	30-34	$37,542	$11,686	$32,434
	35-39	$37,542	$11,686	$32,434
	40-44	$37,542	$11,686	$32,434
	45-49	$29,163	$6,346	$26,483
	50-54	$29,163	$6,346	$26,483
	55-59	$29,163	$6,346	$26,483
	60-64	$29,163	$6,346	$26,483
	65-69	$22,853	$4,846	$24,845
	70-74	$22,853	$4,846	$24,845
	75-79	$13,874	$3,864	$19,358
	80-84	$13,874	$3,864	$19,358
	85-89	$7,635	$1,399	$14,684
	90-94	$7,635	$1,399	$14,684
	95-99	$7,635	$1,399	$14,684

Table 35: Disease costs for oesophageal cancer

Sex	Age group	C	D	E
Male	<1	$44,411	$19,119	$30,879
	1-4	$44,411	$19,119	$30,879
	5-9	$44,411	$19,119	$30,879
	10-14	$44,411	$19,119	$30,879
	15-19	$44,411	$19,119	$30,879
	20-24	$44,411	$19,119	$30,879
	25-29	$44,411	$19,119	$30,879
	30-34	$44,411	$19,119	$30,879
	35-39	$44,411	$19,119	$30,879
	40-44	$44,411	$19,119	$30,879
	45-49	$44,411	$6,800	$30,879
	50-54	$44,411	$6,800	$30,879
	55-59	$44,411	$6,800	$30,879
	60-64	$44,411	$6,800	$30,879
	65-69	$26,343	$5,180	$23,205
	70-74	$26,343	$5,180	$23,205
	75-79	$26,343	$11,355	$25,315
	80-84	$26,343	$11,355	$25,315
	85-89	$26,343	$1,962	$27,424
	90-94	$26,343	$1,962	$27,424
	95-99	$26,343	$1,962	$27,424
Females	<1	$44,782	$6,945	$28,527
	1-4	$44,782	$6,945	$28,527
	5-9	$44,782	$6,945	$28,527
	10-14	$44,782	$6,945	$28,527
	15-19	$44,782	$6,945	$28,527
	20-24	$44,782	$6,945	$28,527
	25-29	$44,782	$6,945	$28,527
	30-34	$44,782	$6,945	$28,527
	35-39	$44,782	$6,945	$28,527
	40-44	$44,782	$6,945	$28,527
	45-49	$44,782	$6,945	$28,527
	50-54	$44,782	$6,945	$28,527
	55-59	$44,782	$6,945	$28,527
	60-64	$44,782	$6,945	$28,527
	65-69	$21,116	$4,233	$21,539
	70-74	$21,116	$4,233	$21,539
	75-79	$21,116	$4,233	$23,497
	80-84	$21,116	$4,233	$23,497
	85-89	$21,116	$4,233	$25,455
	90-94	$21,116	$4,233	$25,455
	95-99	$21,116	$4,233	$25,455

Table 36: Disease costs for osteoarthritis

Sex	Age group	C	D	E
Male	<1	$11,813	$1,717	$29,158
	1-4	$11,813	$1,717	$29,158
	5-9	$6,726	$1,080	$29,158
	10-14	$7,012	$1,226	$29,158
	15-19	$12,578	$2,020	$29,158
	20-24	$9,725	$1,669	$29,158
	25-29	$9,314	$1,591	$29,158
	30-34	$11,104	$1,689	$29,158
	35-39	$12,247	$1,696	$29,158
	40-44	$13,132	$1,731	$29,158
	45-49	$15,559	$2,643	$29,158
	50-54	$15,641	$2,632	$29,158
	55-59	$15,688	$2,628	$40,455
	60-64	$15,758	$2,630	$29,158
	65-69	$15,771	$3,072	$19,266
	70-74	$15,819	$3,063	$32,932
	75-79	$13,474	$2,666	$37,357
	80-84	$13,488	$2,658	$32,233
	85-89	$11,292	$2,239	$43,044
	90-94	$11,331	$2,242	$37,625
	95-99	$10,642	$2,240	$47,587
Females	<1	$15,333	$2,392	$12,556
	1-4	$15,333	$2,392	$12,556
	5-9	$15,333	$1,521	$12,556
	10-14	$14,613	$2,574	$12,556
	15-19	$14,611	$2,714	$12,556
	20-24	$13,414	$2,536	$12,556
	25-29	$14,655	$2,458	$12,556
	30-34	$15,761	$2,500	$12,556
	35-39	$15,597	$2,507	$12,556
	40-44	$15,671	$2,457	$12,556
	45-49	$16,445	$3,031	$12,556
	50-54	$16,543	$3,047	$4,406
	55-59	$16,678	$3,050	$7,298
	60-64	$16,847	$3,035	$14,798
	65-69	$15,379	$2,965	$16,766
	70-74	$15,425	$2,945	$15,663
	75-79	$13,154	$2,596	$13,301
	80-84	$13,117	$2,597	$17,012
	85-89	$9,982	$1,995	$17,151
	90-94	$9,637	$1,990	$21,020
	95-99	$9,476	$1,957	$19,444

Table 37: Disease costs for ovarian cancer

Sex	Age group	C	D	E
Females	<1	$32,610	$2,502	$30,810
	1-4	$32,610	$2,502	$30,810
	5-9	$32,610	$2,502	$30,810
	10-14	$32,610	$2,502	$30,810
	15-19	$32,610	$2,502	$30,810
	20-24	$32,610	$2,502	$30,810
	25-29	$32,610	$2,502	$30,810
	30-34	$32,610	$2,502	$30,810
	35-39	$32,610	$2,502	$30,810
	40-44	$32,610	$2,502	$30,810
	45-49	$25,784	$3,666	$29,785
	50-54	$25,784	$3,666	$29,785
	55-59	$25,784	$3,666	$29,785
	60-64	$25,784	$3,666	$29,785
	65-69	$23,273	$4,370	$29,321
	70-74	$23,273	$4,370	$29,321
	75-79	$18,834	$3,252	$20,103
	80-84	$18,834	$3,252	$20,103
	85-89	$8,810	$2,616	$13,946
	90-94	$8,810	$2,616	$13,946
	95-99	$8,810	$2,616	$13,946

Table 38: Disease costs for pancreatic cancer

Sex	Age group	C	D	E
Male	<1	$49,324	$16,362	$26,068
	1-4	$49,324	$16,362	$26,068
	5-9	$49,324	$16,362	$26,068
	10-14	$49,324	$16,362	$26,068
	15-19	$49,324	$16,362	$26,068
	20-24	$49,324	$16,362	$26,068
	25-29	$49,324	$16,362	$26,068
	30-34	$49,324	$16,362	$26,068
	35-39	$49,324	$16,362	$26,068
	40-44	$49,324	$16,362	$26,068
	45-49	$49,324	$9,372	$26,068
	50-54	$49,324	$9,372	$26,068
	55-59	$49,324	$9,372	$26,068
	60-64	$49,324	$9,372	$26,068
	65-69	$21,440	$6,973	$20,514
	70-74	$21,440	$6,973	$20,514
	75-79	$21,440	$3,234	$22,379
	80-84	$21,440	$3,234	$22,379
	85-89	$21,440	$3,234	$24,244
	90-94	$21,440	$3,234	$24,244
	95-99	$21,440	$3,234	$24,244
Females	<1	$44,011	$16,092	$27,459
	1-4	$44,011	$16,092	$27,459
	5-9	$44,011	$16,092	$27,459
	10-14	$44,011	$16,092	$27,459
	15-19	$44,011	$16,092	$27,459
	20-24	$44,011	$16,092	$27,459
	25-29	$44,011	$16,092	$27,459
	30-34	$44,011	$16,092	$27,459
	35-39	$44,011	$16,092	$27,459
	40-44	$44,011	$16,092	$27,459
	45-49	$42,123	$4,768	$27,459
	50-54	$42,123	$4,768	$27,459
	55-59	$42,123	$4,768	$27,459
	60-64	$42,123	$4,768	$27,459
	65-69	$33,612	$5,017	$18,239
	70-74	$33,612	$5,017	$18,239
	75-79	$18,862	$2,994	$19,898
	80-84	$18,862	$2,994	$19,898
	85-89	$7,284	$2,994	$21,556
	90-94	$7,284	$2,994	$21,556
	95-99	$7,284	$2,994	$21,556

Table 39: Disease costs for stomach cancer

Sex	Age group	C	D	E
Male	<1	$61,445	$2,995	$41,128
	1-4	$61,445	$2,995	$41,128
	5-9	$61,445	$2,995	$41,128
	10-14	$61,445	$2,995	$41,128
	15-19	$61,445	$2,995	$41,128
	20-24	$61,445	$2,995	$41,128
	25-29	$61,445	$2,995	$41,128
	30-34	$61,445	$2,995	$41,128
	35-39	$61,445	$2,995	$41,128
	40-44	$61,445	$2,995	$41,128
	45-49	$45,523	$10,144	$28,604
	50-54	$45,523	$10,144	$28,604
	55-59	$45,523	$10,144	$28,604
	60-64	$45,523	$10,144	$28,604
	65-69	$33,358	$5,509	$30,337
	70-74	$33,358	$5,509	$30,337
	75-79	$22,077	$2,684	$24,161
	80-84	$22,077	$2,684	$24,161
	85-89	$11,780	$1,667	$15,587
	90-94	$11,780	$1,667	$15,587
	95-99	$11,780	$1,667	$15,587
Females	<1	$64,016	$3,668	$41,994
	1-4	$64,016	$3,668	$41,994
	5-9	$64,016	$3,668	$41,994
	10-14	$64,016	$3,668	$41,994
	15-19	$64,016	$3,668	$41,994
	20-24	$64,016	$3,668	$41,994
	25-29	$64,016	$3,668	$41,994
	30-34	$64,016	$3,668	$41,994
	35-39	$64,016	$3,668	$41,994
	40-44	$64,016	$3,668	$41,994
	45-49	$44,627	$7,853	$32,534
	50-54	$44,627	$7,853	$32,534
	55-59	$44,627	$7,853	$32,534
	60-64	$44,627	$7,853	$32,534
	65-69	$30,316	$5,899	$26,579
	70-74	$30,316	$5,899	$26,579
	75-79	$21,720	$4,362	$22,510
	80-84	$21,720	$4,362	$22,510
	85-89	$7,169	$3,438	$12,667
	90-94	$7,169	$3,438	$12,667
	95-99	$7,169	$3,438	$12,667

Table 40: Disease costs for stroke

Sex	Age group	C	D	E
Male	<1	$20,614	$2,797	$18,613
	1-4	$20,614	$2,797	$18,613
	5-9	$20,614	$2,797	$18,613
	10-14	$20,614	$2,797	$18,613
	15-19	$20,614	$2,797	$18,613
	20-24	$20,614	$2,797	$18,613
	25-29	$20,614	$2,797	$18,613
	30-34	$20,614	$2,797	$18,613
	35-39	$20,614	$2,797	$18,613
	40-44	$20,614	$2,797	$18,613
	45-49	$12,689	$3,460	$16,887
	50-54	$12,689	$3,460	$16,887
	55-59	$12,689	$3,460	$16,887
	60-64	$12,689	$3,460	$16,887
	65-69	$9,869	$3,298	$16,394
	70-74	$9,869	$3,298	$16,394
	75-79	$7,733	$2,721	$11,488
	80-84	$7,733	$2,721	$11,488
	85-89	$6,580	$1,996	$9,865
	90-94	$6,580	$1,996	$9,865
	95-99	$6,580	$1,996	$9,865
Females	<1	$23,199	$3,838	$17,974
	1-4	$23,199	$3,838	$17,974
	5-9	$23,199	$3,838	$17,974
	10-14	$23,199	$3,838	$17,974
	15-19	$23,199	$3,838	$17,974
	20-24	$23,199	$3,838	$17,974
	25-29	$23,199	$3,838	$17,974
	30-34	$23,199	$3,838	$17,974
	35-39	$23,199	$3,838	$17,974
	40-44	$23,199	$3,838	$17,974
	45-49	$14,969	$3,547	$18,542
	50-54	$14,969	$3,547	$18,542
	55-59	$14,969	$3,547	$18,542
	60-64	$14,969	$3,547	$18,542
	65-69	$9,918	$3,114	$14,380
	70-74	$9,918	$3,114	$14,380
	75-79	$7,730	$2,596	$10,383
	80-84	$7,730	$2,596	$10,383
	85-89	$6,011	$1,712	$6,501
	90-94	$6,011	$1,712	$6,501
	95-99	$6,011	$1,712	$6,501

Table 41: Disease costs for thyroid cancer

Sex	Age group	C	D	E
Male	<1	$16,485	$3,008	$22,167
	1-4	$16,485	$3,008	$22,167
	5-9	$16,485	$3,008	$22,167
	10-14	$16,485	$3,008	$22,167
	15-19	$16,485	$3,008	$22,167
	20-24	$16,485	$3,008	$22,167
	25-29	$16,485	$3,008	$22,167
	30-34	$16,485	$3,008	$22,167
	35-39	$16,485	$3,008	$22,167
	40-44	$16,485	$3,008	$22,167
	45-49	$19,754	$3,620	$22,167
	50-54	$19,754	$3,620	$22,167
	55-59	$19,754	$3,620	$22,167
	60-64	$19,754	$3,620	$22,167
	65-69	$16,181	$5,140	$24,383
	70-74	$16,181	$5,140	$24,383
	75-79	$10,220	$3,063	$26,600
	80-84	$10,220	$3,063	$26,600
	85-89	$10,220	$3,063	$28,817
	90-94	$10,220	$3,063	$28,817
	95-99	$10,220	$3,063	$28,817
Females	<1	$13,702	$1,542	$29,136
	1-4	$13,702	$1,542	$29,136
	5-9	$13,702	$1,542	$29,136
	10-14	$13,702	$1,542	$29,136
	15-19	$13,702	$1,542	$29,136
	20-24	$13,702	$1,542	$29,136
	25-29	$13,702	$1,542	$29,136
	30-34	$13,702	$1,542	$29,136
	35-39	$13,702	$1,542	$29,136
	40-44	$13,702	$1,542	$29,136
	45-49	$10,760	$2,415	$29,136
	50-54	$10,760	$2,415	$29,136
	55-59	$10,760	$2,415	$29,136
	60-64	$10,760	$2,415	$29,136
	65-69	$11,709	$2,560	$32,050
	70-74	$11,709	$2,560	$32,050
	75-79	$11,022	$2,926	$34,963
	80-84	$11,022	$2,926	$34,963
	85-89	$11,022	$2,165	$37,877
	90-94	$11,022	$2,165	$37,877
	95-99	$11,022	$2,165	$37,877

Appendix G: Model rates vs. DISMOD rates, log graphs from Section 3.04.5 (page 49 to 54)

(i) Model outputs into the future, log scale: CHD

Figure 18: CHD disease rates: DIET model versus DISMOD outputs for non-Māori male, starting age 2 on a log scale	Figure 19: CHD disease rates: DIET model versus DISMOD outputs for Māori male, starting age 2 on a log scale
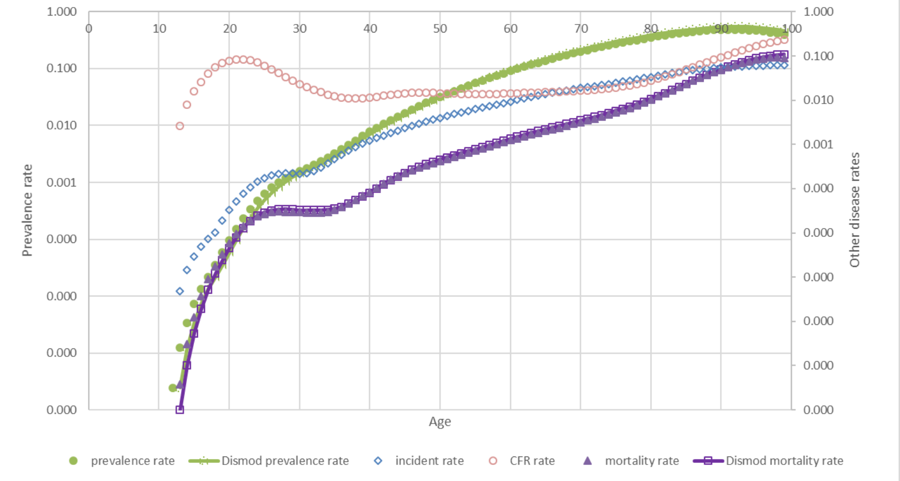	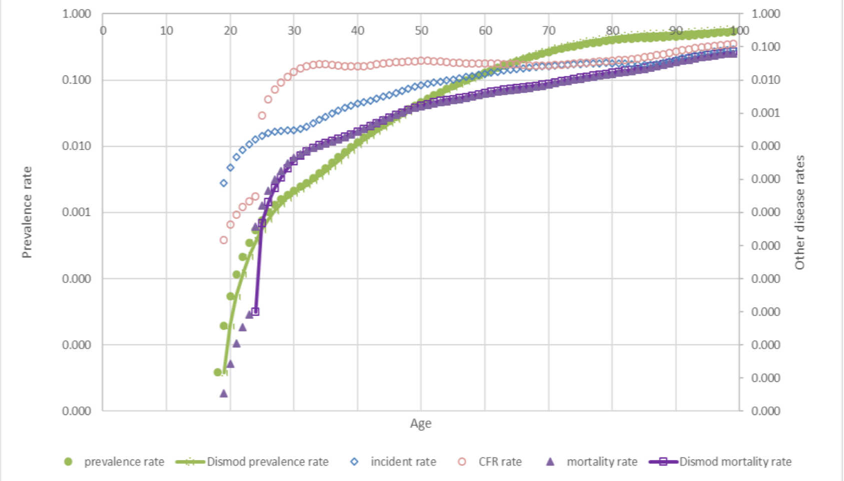

Figure 20: CHD disease rates: DIET model versus DISMOD outputs non-Māori female, starting age 2 on a log scale

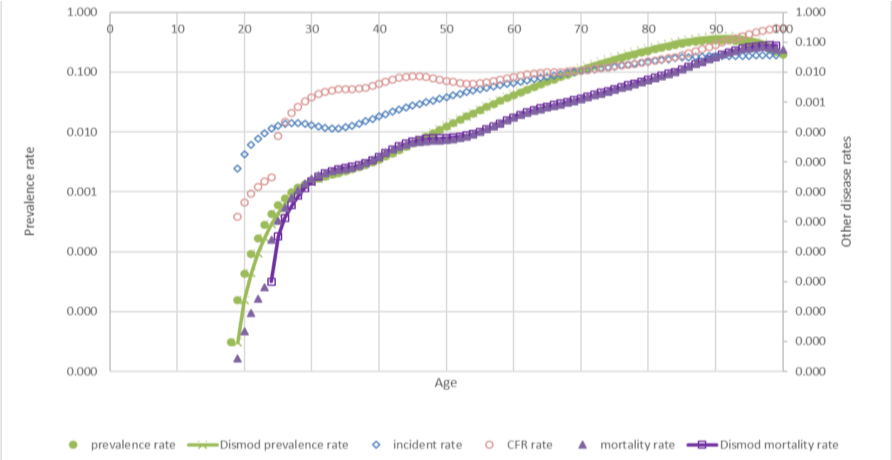

Figure 21:CHD disease rates: DIET model versus DISMOD outputs for Māori female, starting age 2 on a log scale

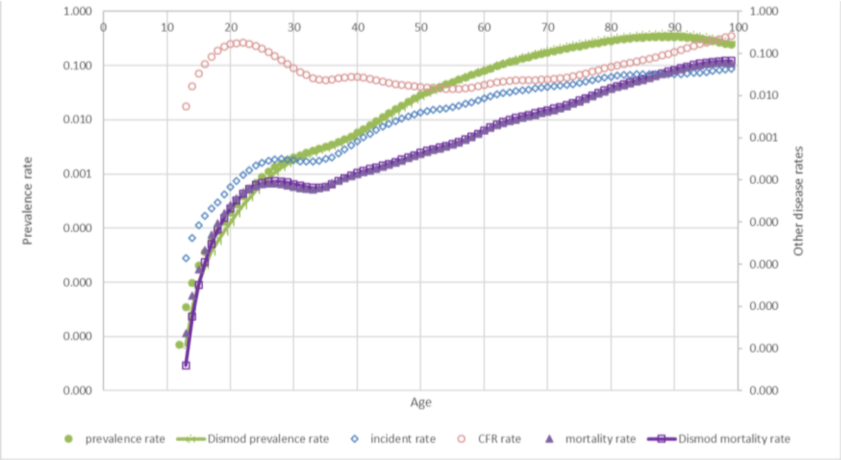

Figure 22: CHD disease rates: DIET model versus DISMOD outputs for non-Māori male, starting age 42 on a log scale

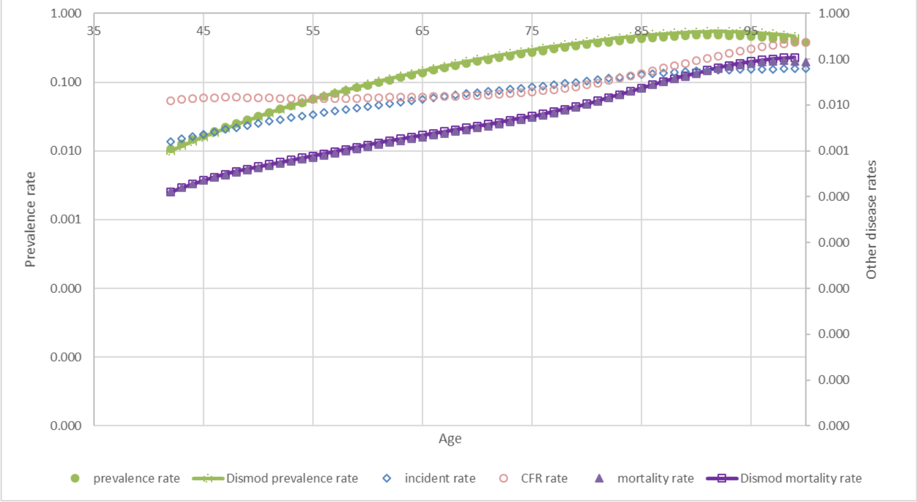

Figure 23: CHD disease rates: DIET model versus DISMOD outputs for non-Māori male, starting age 72 on a log scale

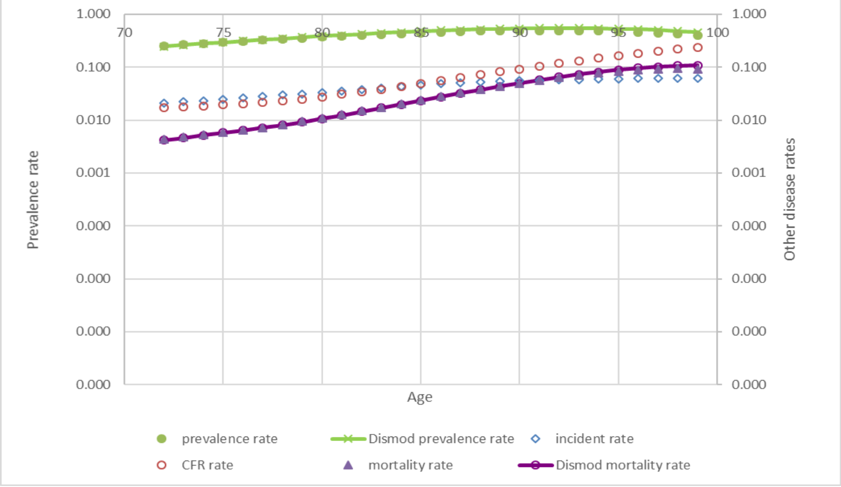

(ii) Model outputs into the future, log scale: stroke

Figure 24: Stroke disease rates: DIET model versus DISMOD outputs for non-Māori male, starting age 2 on a log scale

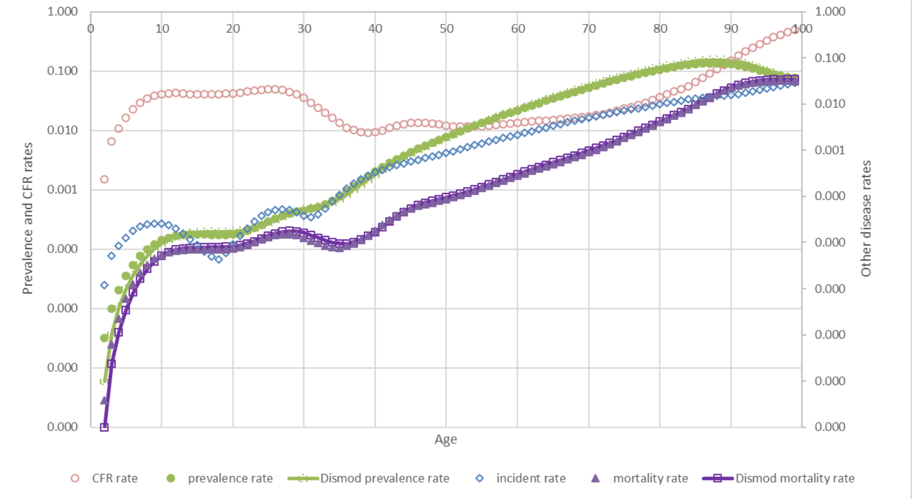

Figure 25: Stroke disease rates: DIET model versus DISMOD outputs for Māori male, starting age 2 on a log scale

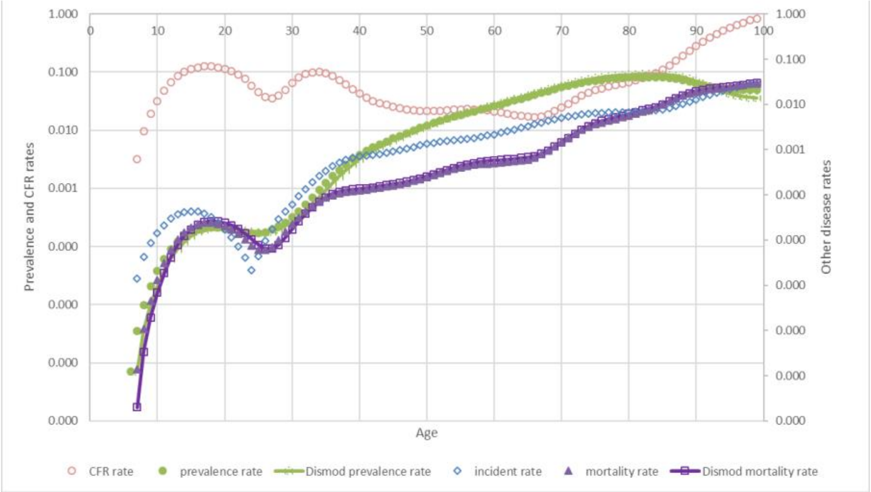

Figure 26: Stroke disease rates: DIET model versus DISMOD outputs non-Māori female, starting age 2 on a log scale

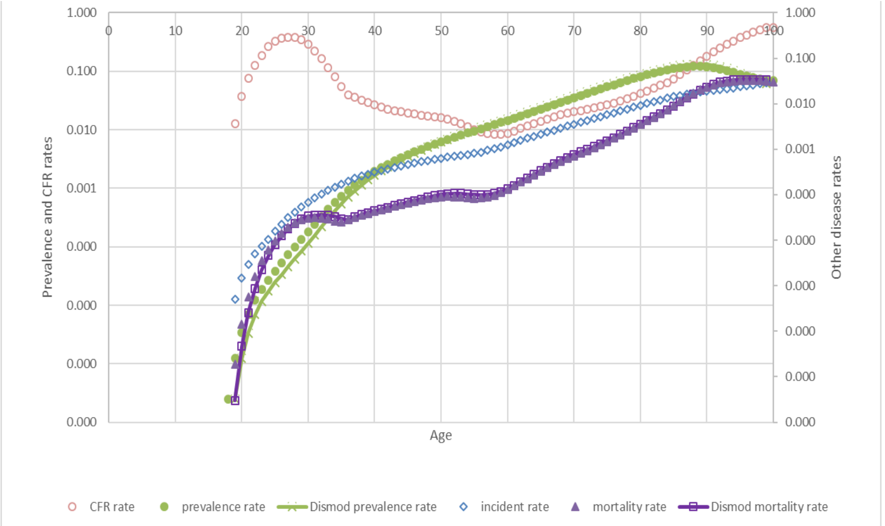

Figure 27: Stroke disease rates: DIET model versus DISMOD outputs for Māori female, starting age 2 on a log scale

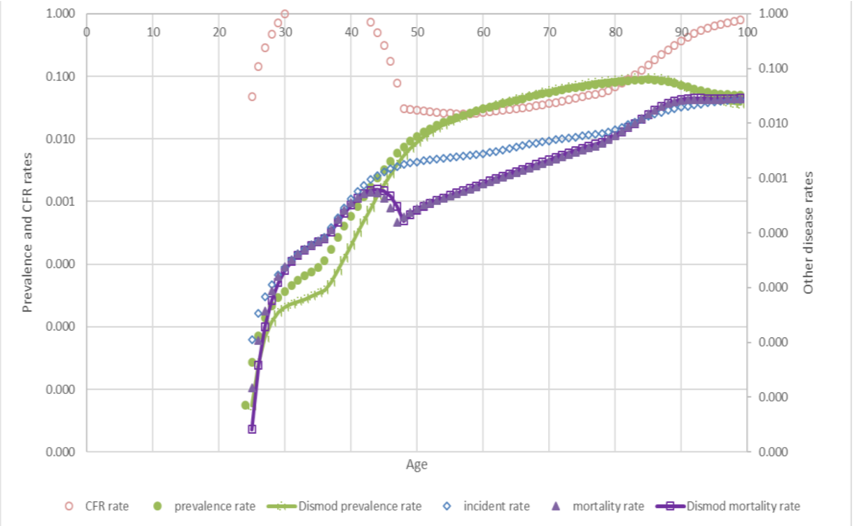

Figure 28: Stroke disease rates: DIET model versus DISMOD outputs for non-Māori male, starting age 42 on a log scale

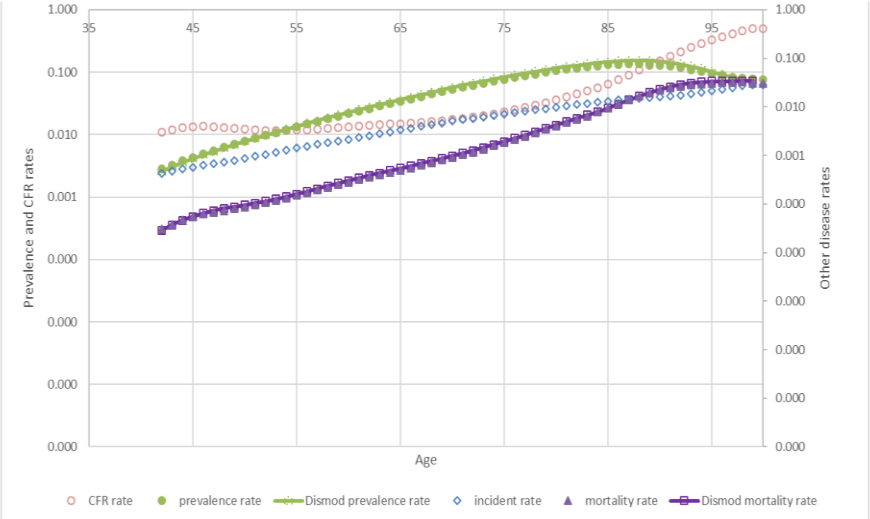

Figure 29: Stroke disease rates: DIET model versus DISMOD outputs for non-Māori male, starting age 72 on a log scale

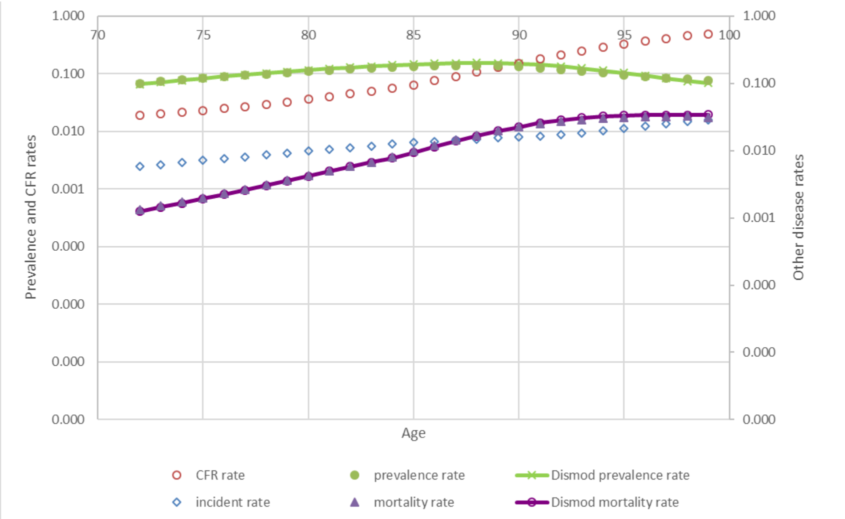

(iii) Model outputs into the future, log scale: diabetes

Figure 30: Diabetes disease rates: DIET model versus DISMOD outputs for non-Māori male, starting age 2 on a log scale	Figure 31: Diabetes disease rates: DIET model versus DISMOD outputs for Māori male, starting age 2 on a log scale
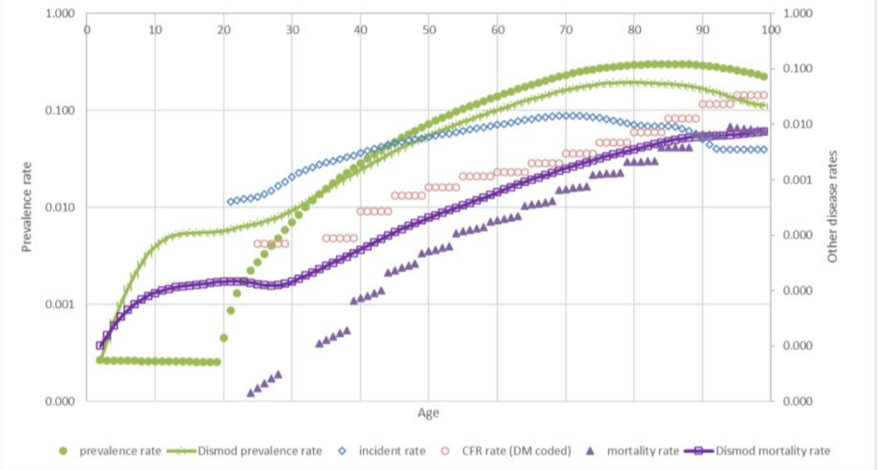	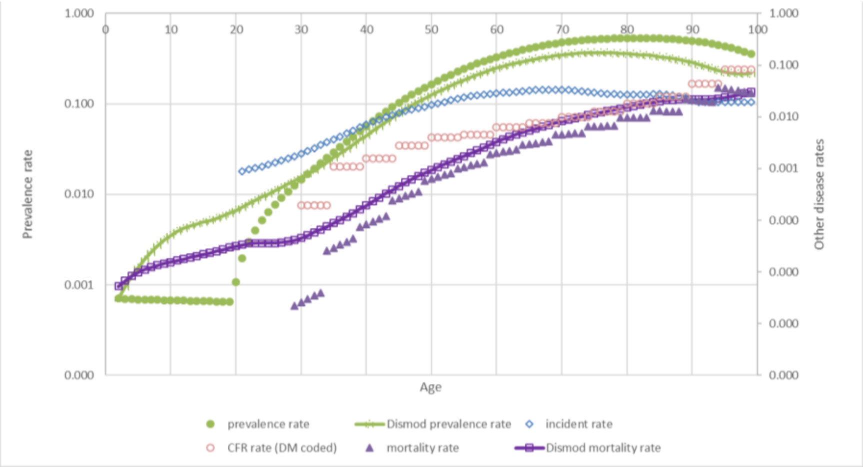

Figure 32: Diabetes disease rates: DIET model versus DISMOD outputs non-Māori female, starting age 2 on a log scale

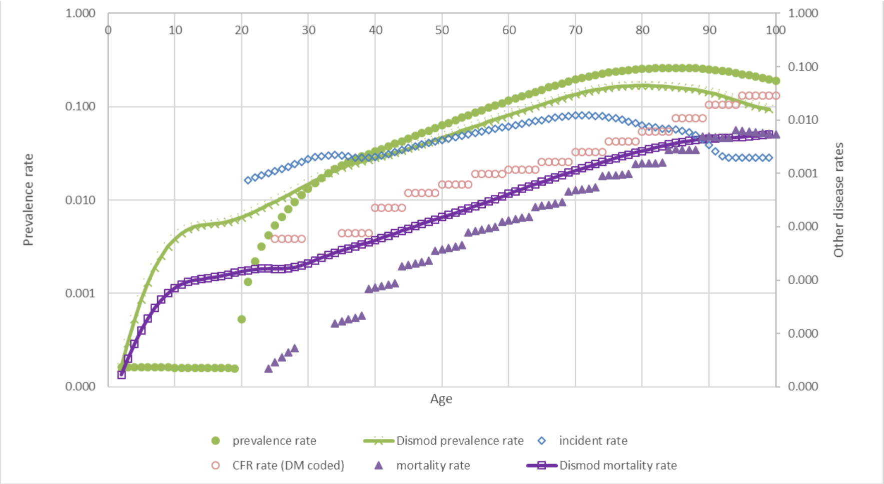

Figure 33: Diabetes disease rates: DIET model versus DISMOD outputs for Māori female, starting age 2 on a log scale

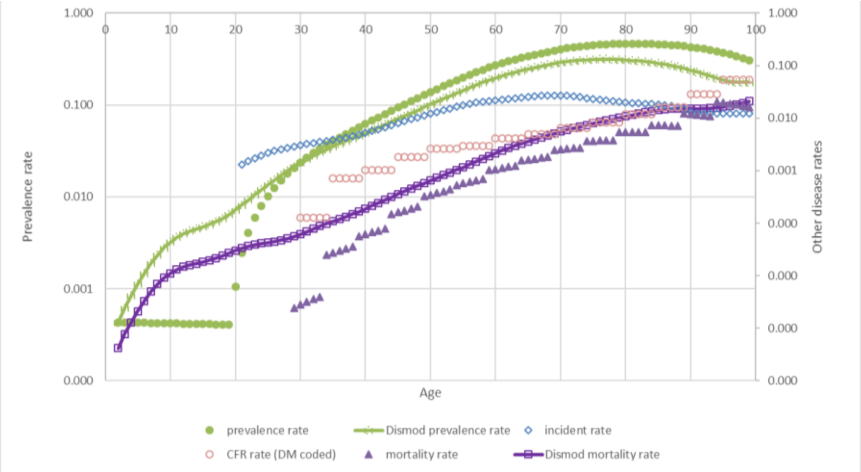

Figure 34: Diabetes disease rates: DIET model versus DISMOD outputs for non-Māori male, starting age 42 on a log scale

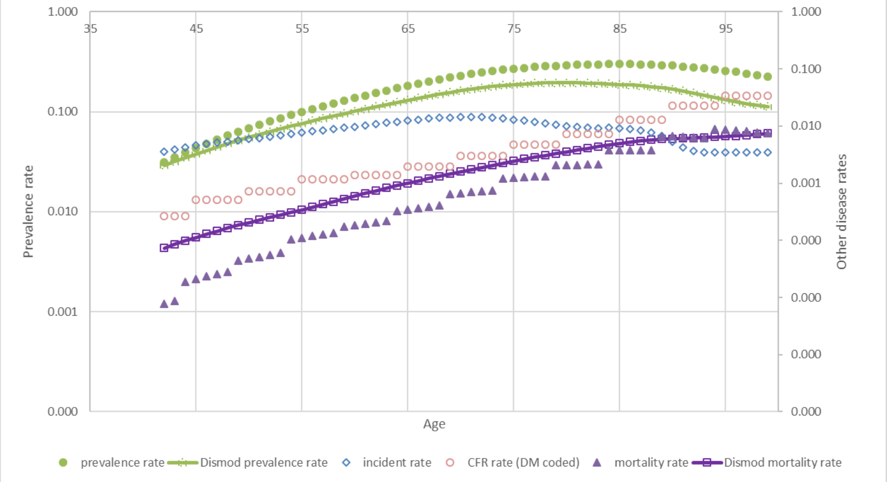

Figure 35: Diabetes disease rates: DIET model versus DISMOD outputs for non-Māori male, starting age 72 on a log scale

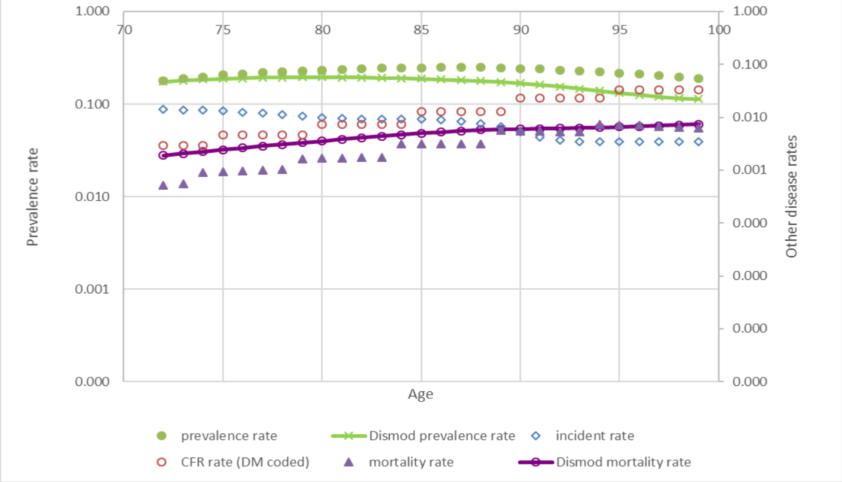

Supplement: Multimedia Appendix 1 [file mhealth_v7i1e11118_app1.pdf]
